# Supplementary material for: Non-calyceal inputs gate the timing of calyx of Held evoked MNTB output
Source: Commun Biol. 2026 May 22;9:697. doi: 10.1038/s42003-026-10321-w (PMC13197447; doi:10.1038/s42003-026-10321-w)
Supplement: Supplementary file 1 — Description of Additional Supplementary File [file 42003_2026_10321_MOESM1_ESM.pdf]

Description of Additional Supplementary File

File name: Supplementary data
Description: The Excel file contains all presented data listed according to the figures. Next to the data the file contains the details of the statistical analysis for each figure.

File name: Supplementary Code
Description: The original IgorPro ipf files contain custom written code for the analysis of electrophysiological data were converted into pdf files. Trains_AMPCharg: Analyses short EPSC trains. asynchronous: Analyses delayed release after stimulation trains. CalyxDC_dendriteSTIM: Analyses the timing and voltage deflections of train stimulation and the action potential generation in experiments of simultaneous dynamic clamp and synaptic stimulation. FitthefirstEPSCLong and FittheFirstEPSC analyses the EPSC kinetics and the long stimulation trains.
