# Supplementary material for: Non-calyceal inputs gate the timing of calyx of Held evoked MNTB output
Source: Commun Biol. 2026 May 22;9:697. doi: 10.1038/s42003-026-10321-w (PMC13197447; doi:10.1038/s42003-026-10321-w)
Supplement: Supplementary file 3 — Supplementary code [file 42003_2026_10321_MOESM3_ESM.pdf]

```
#pragma TextEncoding = "UTF-8"

#pragma rtGlobals=3				// Use modern global access method and strict
wave access

#pragma DefaultTab={3,20,4}		// Set default tab width in Igor Pro 9 and later




Menu "Macros"

	"Isolate the asyn. release", /Q, asyn_release()

	"Scale to zero", /Q,Scaling()

	"Prep waves", /Q,	Prepwaves()

	//"Average release", /Q, Average()

	//"RemoveNoise", /Q, RemoveNoise()

	"Find Peaks", /Q, AutoFindPeaksCustom()

	"Excel export 10 Hz", /Q,Exportmetoexcel10()

	"Excel export 50 Hz", /Q,Exportmetoexcel50()

	"Excel export 100 Hz", /Q,Exportmetoexcel100()

	"Excel export 200 Hz", /Q,Exportmetoexcel200()

	"Excel export 300 Hz", /Q,Exportmetoexcel300()

	"Excel export 400 Hz", /Q,Exportmetoexcel400()

End



macro asyn_release()


NewDataFolder Asynchron_release

// Set Folder to root//


//10 Hz//


Duplicate/R=[146858,171759] :Integration:'10_Graphs':platz_original_10_rep_0 release10_0

Duplicate/R=[146858,171759] :Integration:'10_Graphs':platz_original_10_rep_1 release10_1
```

Duplicate/R=[146858,171759] :Integration:'10_Graphs':platz_original_10_rep_2 release10_2

//50 Hz//

Duplicate/R=[30857,55759] :Integration:'50_Graphs':platz_original_50_rep_0 release50_0

Duplicate/R=[30857,55759] :Integration:'50_Graphs':platz_original_50_rep_1 release50_1

Duplicate/R=[30857,55759] :Integration:'50_Graphs':platz_original_50_rep_2 release50_2

//100 Hz//

Duplicate/R=[16346,41259] :Integration:'100_Graphs':platz_original_100_rep_0 release100_0

Duplicate/R=[16346,41259] :Integration:'100_Graphs':platz_original_100_rep_1 release100_1

Duplicate/R=[16346,41259] :Integration:'100_Graphs':platz_original_100_rep_2 release100_2

//200 Hz//

Duplicate/R=[9105,34009] :Integration:'200_Graphs':platz_original_200_rep_0 release200_0

Duplicate/R=[9105,34009] :Integration:'200_Graphs':platz_original_200_rep_1 release200_1

Duplicate/R=[9105,34009] :Integration:'200_Graphs':platz_original_200_rep_2 release200_2

//300 Hz//

Duplicate/R=[6627,31544] :Integration:'300_Graphs':platz_original_300_rep_0 release300_0

Duplicate/R=[6627,31544] :Integration:'300_Graphs':platz_original_300_rep_1 release300_1

Duplicate/R=[6627,31544] :Integration:'300_Graphs':platz_original_300_rep_2 release300_2

//400 Hz//

Duplicate/R=[5479,30384] :Integration:'400_Graphs':platz_original_400_rep_0 release400_0

Duplicate/R=[5479,30384] :Integration:'400_Graphs':platz_original_400_rep_1 release400_1

Duplicate/R=[5479,30384] :Integration:'400_Graphs':platz_original_400_rep_2 release400_2

```
//Bring everythin in structure
SetDataFolder Asynchron_release
NewDataFolder release10Hz
NewDataFolder release50Hz
NewDataFolder release100Hz
NewDataFolder release200Hz
NewDataFolder release300Hz
NewDataFolder release400Hz

MoveWave root:release10_0, :release10Hz:
MoveWave root:release10_1, :release10Hz:
MoveWave root:release10_2, :release10Hz:

MoveWave root:release50_0, :release50Hz:
MoveWave root:release50_1, :release50Hz:
MoveWave root:release50_2, :release50Hz:

MoveWave root:release100_0, :release100Hz:
MoveWave root:release100_1, :release100Hz:
MoveWave root:release100_2, :release100Hz:

MoveWave root:release200_0, :release200Hz:
MoveWave root:release200_1, :release200Hz:
MoveWave root:release200_2, :release200Hz:

MoveWave root:release300_0, :release300Hz:
MoveWave root:release300_1, :release300Hz:
MoveWave root:release300_2, :release300Hz:

MoveWave root:release400_0, :release400Hz:
MoveWave root:release400_1, :release400Hz:
MoveWave root:release400_2, :release400Hz:
```

```
end

// SET THE RLEASE_FREQ FOLDER ALS ROOT
////////////////////////////////////////////////////////////////////////////////

Function Scaling() //not relevant

String Original_Waves
Original_Waves=WaveList("release*",";",(""))
variable n

for(n=0;n<itemsinlist(Original_Waves);n+=1)
	SetScale/P x 0,2e-5, "s", $StringFromList(n,Original_Waves)


endfor


end

////////////////////////////////////////////////////////////////////////////////
Function Prepwaves()

String Original_Waves
Original_Waves=WaveList("release*",";",(""))
variable n

for(n=0;n<itemsinlist(Original_Waves);n+=1)
	Duplicate $StringFromList(n,Original_Waves), $"release"+num2str(n)
```

```
		//SetDataFolder Release

endfor

NewDataFolder/O Release

for(n=0;n<itemsinlist(Original_Waves);n+=1)
MoveWave $"release"+num2str(n), :Release:
//Display $"release"+num2str(n), :Release:
endfor

SetDataFolder Release
wave release0
wave release1
wave release2

release0*=(-1)
release1*=(-1)
release2*=(-1)

NewDataFolder/O Release0
NewDataFolder/O Release1
NewDataFolder/O Release2
MoveWave release0, :Release0:
MoveWave release1, :Release1:
MoveWave release2, :Release2:
end
//////////////////////////////////////////////
Function Average() //not relevant
String Original_Waves
Original_Waves=WaveList("release*",";",(""))
```

```
variable n

for(n=0;n<itemsinlist(Original_Waves);n+=1)
	Duplicate $StringFromList(n,Original_Waves), $"release"+num2str(n)
endfor

NewDataFolder/O AVG

for(n=0;n<itemsinlist(Original_Waves);n+=1)
MoveWave $"release"+num2str(n), :AVG:
endfor

SetDataFolder AVG
wave release0
wave release1
wave release2
fWaveAverage(WaveList("release*", ";", ""), "", 3, 3, "Release_Avg", "Release_sem")

KillWaves release0,release1,release2

wave Release_Avg, Release_Avg_Invert
Duplicate Release_Avg, Release_Avg_Invert
Release_Avg_Invert*=(-1)
end
/////////////////////////////////////////

Macro RemoveNoise()

Release-=3.3917e-11 // set value here
Release[][] = Release[p][q] < 0 ? 0 : Release[p][q]
endmacro
```

```
//////////////////

Macro Exportmetoexcel10()

SetDataFolder root:Asynchron_release:release10Hz:Release:

SetDataFolder Release0

Display release0

AppendToGraph WA_PeakCentersY vs WA_PeakCentersX

ModifyGraph
rgb(Release0)=(31868,40092,51914),mode(WA_PeakCentersY)=3,marker(WA_PeakCentersY)=1
9,rgb(WA_PeakCentersY)=(5397,17990,37265)

Edit W_AutoPeakInfo,WA_PeakCentersX,WA_PeakCentersY


SetDataFolder root:Asynchron_release:release10Hz:Release:

SetDataFolder Release1

Display release1

AppendToGraph WA_PeakCentersY vs WA_PeakCentersX

ModifyGraph
rgb(Release1)=(31868,40092,51914),mode(WA_PeakCentersY)=3,marker(WA_PeakCentersY)=1
9,rgb(WA_PeakCentersY)=(5397,17990,37265)

Edit W_AutoPeakInfo,WA_PeakCentersX,WA_PeakCentersY


SetDataFolder root:Asynchron_release:release10Hz:Release:

SetDataFolder Release2

Display release2

AppendToGraph WA_PeakCentersY vs WA_PeakCentersX

ModifyGraph
rgb(Release2)=(31868,40092,51914),mode(WA_PeakCentersY)=3,marker(WA_PeakCentersY)=1
9,rgb(WA_PeakCentersY)=(5397,17990,37265)

Edit W_AutoPeakInfo,WA_PeakCentersX,WA_PeakCentersY


SetDataFolder root:Asynchron_release:release10Hz:AVG:

Display Release_Avg_Invert

AppendToGraph WA_PeakCentersY vs WA_PeakCentersX

ModifyGraph
rgb(Release_Avg_Invert)=(31868,40092,51914),mode(WA_PeakCentersY)=3,marker(WA_PeakC
entersY)=19,rgb(WA_PeakCentersY)=(5397,17990,37265)
```

```
Edit W_AutoPeakInfo,WA_PeakCentersX,WA_PeakCentersY

endmacro


//////////////////

Macro Exportmetoexcel50()

SetDataFolder root:Asynchron_release:release50Hz:Release:

SetDataFolder Release0

Display release0

AppendToGraph WA_PeakCentersY vs WA_PeakCentersX

ModifyGraph
rgb(Release0)=(31868,40092,51914),mode(WA_PeakCentersY)=3,marker(WA_PeakCentersY)=1
9,rgb(WA_PeakCentersY)=(5397,17990,37265)

Edit W_AutoPeakInfo,WA_PeakCentersX,WA_PeakCentersY


SetDataFolder root:Asynchron_release:release50Hz:Release:

SetDataFolder Release1

Display release1

AppendToGraph WA_PeakCentersY vs WA_PeakCentersX

ModifyGraph
rgb(Release1)=(31868,40092,51914),mode(WA_PeakCentersY)=3,marker(WA_PeakCentersY)=1
9,rgb(WA_PeakCentersY)=(5397,17990,37265)

Edit W_AutoPeakInfo,WA_PeakCentersX,WA_PeakCentersY


SetDataFolder root:Asynchron_release:release50Hz:Release:

SetDataFolder Release2

Display release2

AppendToGraph WA_PeakCentersY vs WA_PeakCentersX

ModifyGraph
rgb(Release2)=(31868,40092,51914),mode(WA_PeakCentersY)=3,marker(WA_PeakCentersY)=1
9,rgb(WA_PeakCentersY)=(5397,17990,37265)

Edit W_AutoPeakInfo,WA_PeakCentersX,WA_PeakCentersY


SetDataFolder root:Asynchron_release:release50Hz:AVG:

Display Release_Avg_Invert
```

```
AppendToGraph WA_PeakCentersY vs WA_PeakCentersX

ModifyGraph
rgb(Release_Avg_Invert)=(31868,40092,51914),mode(WA_PeakCentersY)=3,marker(WA_PeakC
entersY)=19,rgb(WA_PeakCentersY)=(5397,17990,37265)

Edit W_AutoPeakInfo,WA_PeakCentersX,WA_PeakCentersY

endmacro


//////////////////

Macro Exportmetoexcel100()

SetDataFolder root:Asynchron_release:release100Hz:Release:

SetDataFolder Release0

Display release0

AppendToGraph WA_PeakCentersY vs WA_PeakCentersX

ModifyGraph
rgb(Release0)=(31868,40092,51914),mode(WA_PeakCentersY)=3,marker(WA_PeakCentersY)=1
9,rgb(WA_PeakCentersY)=(5397,17990,37265)

Edit W_AutoPeakInfo,WA_PeakCentersX,WA_PeakCentersY


SetDataFolder root:Asynchron_release:release100Hz:Release:

SetDataFolder Release1

Display release1

AppendToGraph WA_PeakCentersY vs WA_PeakCentersX

ModifyGraph
rgb(Release1)=(31868,40092,51914),mode(WA_PeakCentersY)=3,marker(WA_PeakCentersY)=1
9,rgb(WA_PeakCentersY)=(5397,17990,37265)

Edit W_AutoPeakInfo,WA_PeakCentersX,WA_PeakCentersY


SetDataFolder root:Asynchron_release:release100Hz:Release:

SetDataFolder Release2

Display release2

AppendToGraph WA_PeakCentersY vs WA_PeakCentersX

ModifyGraph
rgb(Release2)=(31868,40092,51914),mode(WA_PeakCentersY)=3,marker(WA_PeakCentersY)=1
9,rgb(WA_PeakCentersY)=(5397,17990,37265)

Edit W_AutoPeakInfo,WA_PeakCentersX,WA_PeakCentersY
```

```
SetDataFolder root:Asynchron_release:release100Hz:AVG:

Display Release_Avg_Invert

AppendToGraph WA_PeakCentersY vs WA_PeakCentersX

ModifyGraph
rgb(Release_Avg_Invert)=(31868,40092,51914),mode(WA_PeakCentersY)=3,marker(WA_PeakC
entersY)=19,rgb(WA_PeakCentersY)=(5397,17990,37265)

Edit W_AutoPeakInfo,WA_PeakCentersX,WA_PeakCentersY

endmacro

//////////////////

Macro Exportmetoexcel200()

SetDataFolder root:Asynchron_release:release200Hz:Release:

SetDataFolder Release0

Display release0

AppendToGraph WA_PeakCentersY vs WA_PeakCentersX

ModifyGraph
rgb(Release0)=(31868,40092,51914),mode(WA_PeakCentersY)=3,marker(WA_PeakCentersY)=1
9,rgb(WA_PeakCentersY)=(5397,17990,37265)

Edit W_AutoPeakInfo,WA_PeakCentersX,WA_PeakCentersY


SetDataFolder root:Asynchron_release:release200Hz:Release:

SetDataFolder Release1

Display release1

AppendToGraph WA_PeakCentersY vs WA_PeakCentersX

ModifyGraph
rgb(Release1)=(31868,40092,51914),mode(WA_PeakCentersY)=3,marker(WA_PeakCentersY)=1
9,rgb(WA_PeakCentersY)=(5397,17990,37265)

Edit W_AutoPeakInfo,WA_PeakCentersX,WA_PeakCentersY


SetDataFolder root:Asynchron_release:release200Hz:Release:

SetDataFolder Release2

Display release2

AppendToGraph WA_PeakCentersY vs WA_PeakCentersX
```

```
ModifyGraph
rgb(Release2)=(31868,40092,51914),mode(WA_PeakCentersY)=3,marker(WA_PeakCentersY)=1
9,rgb(WA_PeakCentersY)=(5397,17990,37265)

Edit W_AutoPeakInfo,WA_PeakCentersX,WA_PeakCentersY


SetDataFolder root:Asynchron_release:release200Hz:AVG:

Display Release_Avg_Invert

AppendToGraph WA_PeakCentersY vs WA_PeakCentersX

ModifyGraph
rgb(Release_Avg_Invert)=(31868,40092,51914),mode(WA_PeakCentersY)=3,marker(WA_PeakC
entersY)=19,rgb(WA_PeakCentersY)=(5397,17990,37265)

Edit W_AutoPeakInfo,WA_PeakCentersX,WA_PeakCentersY

endmacro


///////////////////

Macro Exportmetoexcel300()

SetDataFolder root:Asynchron_release:release300Hz:Release:

SetDataFolder Release0

Display release0

AppendToGraph WA_PeakCentersY vs WA_PeakCentersX

ModifyGraph
rgb(Release0)=(31868,40092,51914),mode(WA_PeakCentersY)=3,marker(WA_PeakCentersY)=1
9,rgb(WA_PeakCentersY)=(5397,17990,37265)

Edit W_AutoPeakInfo,WA_PeakCentersX,WA_PeakCentersY


SetDataFolder root:Asynchron_release:release300Hz:Release:

SetDataFolder Release1

Display release1

AppendToGraph WA_PeakCentersY vs WA_PeakCentersX

ModifyGraph
rgb(Release1)=(31868,40092,51914),mode(WA_PeakCentersY)=3,marker(WA_PeakCentersY)=1
9,rgb(WA_PeakCentersY)=(5397,17990,37265)

Edit W_AutoPeakInfo,WA_PeakCentersX,WA_PeakCentersY


SetDataFolder root:Asynchron_release:release300Hz:Release:
```

```
SetDataFolder Release2

Display release2

AppendToGraph WA_PeakCentersY vs WA_PeakCentersX

ModifyGraph
rgb(Release2)=(31868,40092,51914),mode(WA_PeakCentersY)=3,marker(WA_PeakCentersY)=1
9,rgb(WA_PeakCentersY)=(5397,17990,37265)

Edit W_AutoPeakInfo,WA_PeakCentersX,WA_PeakCentersY


SetDataFolder root:Asynchron_release:release300Hz:AVG:

Display Release_Avg_Invert

AppendToGraph WA_PeakCentersY vs WA_PeakCentersX

ModifyGraph
rgb(Release_Avg_Invert)=(31868,40092,51914),mode(WA_PeakCentersY)=3,marker(WA_PeakC
entersY)=19,rgb(WA_PeakCentersY)=(5397,17990,37265)

Edit W_AutoPeakInfo,WA_PeakCentersX,WA_PeakCentersY

endmacro


//////////////////

Macro Exportmetoexcel400()

SetDataFolder root:Asynchron_release:release400Hz:Release:

SetDataFolder Release0

Display release0

AppendToGraph WA_PeakCentersY vs WA_PeakCentersX

ModifyGraph
rgb(Release0)=(31868,40092,51914),mode(WA_PeakCentersY)=3,marker(WA_PeakCentersY)=1
9,rgb(WA_PeakCentersY)=(5397,17990,37265)

Edit W_AutoPeakInfo,WA_PeakCentersX,WA_PeakCentersY


SetDataFolder root:Asynchron_release:release400Hz:Release:

SetDataFolder Release1

Display release1

AppendToGraph WA_PeakCentersY vs WA_PeakCentersX

ModifyGraph
rgb(Release1)=(31868,40092,51914),mode(WA_PeakCentersY)=3,marker(WA_PeakCentersY)=1
9,rgb(WA_PeakCentersY)=(5397,17990,37265)
```

```
Edit W_AutoPeakInfo,WA_PeakCentersX,WA_PeakCentersY


SetDataFolder root:Asynchron_release:release400Hz:Release:

SetDataFolder Release2

Display release2

AppendToGraph WA_PeakCentersY vs WA_PeakCentersX

ModifyGraph
rgb(Release2)=(31868,40092,51914),mode(WA_PeakCentersY)=3,marker(WA_PeakCentersY)=1
9,rgb(WA_PeakCentersY)=(5397,17990,37265)

Edit W_AutoPeakInfo,WA_PeakCentersX,WA_PeakCentersY


SetDataFolder root:Asynchron_release:release400Hz:AVG:

Display Release_Avg_Invert

AppendToGraph WA_PeakCentersY vs WA_PeakCentersX

ModifyGraph
rgb(Release_Avg_Invert)=(31868,40092,51914),mode(WA_PeakCentersY)=3,marker(WA_PeakC
entersY)=19,rgb(WA_PeakCentersY)=(5397,17990,37265)

Edit W_AutoPeakInfo,WA_PeakCentersX,WA_PeakCentersY

endmacro

//////////////////////




Function AutoFindPeaksCustom()
                                        //hier alles was fürs Window gebraucht wird

                    String wname, xdata="_calculated_"

          Prompt wname, "Peak Wave", popup, WaveList("release*",";",(""))+"_none_;"              //hier
zeigt er mir all die waves an, die displayed sind

                    Prompt xdata, "X values", popup, "_calculated_"
          //hier zeigt er mir nur die calculated x data an

                    DoPrompt "Automatically Find Peaks Custom Parameters", wname, xdata //Das
der Text von dem Window das aufploppt

          if( V_Flag != 0 )

                    return 0
                                                            // user cancelled

          endif
```

```
	WAVE/Z w=$wname
	WAVE/Z wx=$xdata
	Variable pBegin=0, pEnd= numpnts(w)-1


	Variable maxPeaks=100, minPeakPercent=4.5
			//hier werden die automatischen eingefügten Werte festgelegt
	Variable/C estimates= EstPeakNoiseAndSmfact(w,pBegin, pEnd)
	//verweißt auf andere Funktion, in der das Noise estimated wird
	Variable noiselevel=real(estimates)
	Variable smoothingFactor=imag(estimates)
	Prompt maxPeaks, "Maximum Peaks"
	Prompt minPeakPercent, "Minimum Peak Amplitude (% max)"
	Prompt noiseLevel, "Noise level"
	Prompt smoothingFactor, "Smoothing Factor"
	DoPrompt "Automatically Find Peaks", maxPeaks, minPeakPercent, noiseLevel,
smoothingFactor
	if( V_Flag != 0 )
		return 0
							// user cancelled
	endif


	AutoFindPeaksWorker(w, wx, pBegin, pEnd, maxPeaks, minPeakPercent, noiseLevel,
smoothingFactor) //verweißt auf die Function, die mir dann die peaks findet
end


//	FINDING THE PEAKS //


Function AutoFindPeaksWorker(w, wx, pBegin, pEnd, maxPeaks, minPeakPercent, noiseLevel,
smoothingFactor)
	WAVE w
	WAVE/Z wx
	Variable pBegin, pEnd
	Variable maxPeaks, minPeakPercent, noiseLevel, smoothingFactor
```

```
	Variable peaksFound=
AutoFindPeaks(w,pBegin,pEnd,noiseLevel,smoothingFactor,maxPeaks)
	//verweißt auf die AutoFindPeak-Function, s.U.

	if( peaksFound > 0 )

		WAVE W_AutoPeakInfo

		// Remove too-small peaks

		peaksFound= TrimAmpAutoPeakInfo(W_AutoPeakInfo,minPeakPercent/100)
							//verweißt auf die AutoFindPeak-Function,
s.U.

		if( peaksFound > 0 )

			// Make waves to display in a graph

			// The x values in W_AutoPeakInfo are still actually points, not X

			Make/O/N=(peaksFound) WA_PeakCentersY = w[W_AutoPeakInfo[p][0]]

			AdjustAutoPeakInfoForX(W_AutoPeakInfo,w,wx)

			Make/O/N=(peaksFound) WA_PeakCentersX = W_AutoPeakInfo[p][0]


			// Show W_AutoPeakInfo in a table, with dimension labels

			SetDimLabel 1, 0, center, W_AutoPeakInfo

			SetDimLabel 1, 1, width, W_AutoPeakInfo

			SetDimLabel 1, 2, height, W_AutoPeakInfo

			CheckDisplayed/A W_AutoPeakInfo

			if( V_Flag == 0 )

				Edit W_AutoPeakInfo.ld

			endif


			DoWindow ShowPeaks

			if( V_Flag == 0 )

				if( WaveExists(wx) )

					Display/N=ShowPeaks w vs wx

				else

					Display/N=ShowPeaks w
```

```
				endif
				AppendToGraph/W=ShowPeaks WA_PeakCentersY vs WA_PeakCentersX
				ModifyGraph/W=ShowPeaks rgb(WA_PeakCentersY)=(5397,17990,37265)
				ModifyGraph/W=ShowPeaks rgb(Release0)=(31868,40092,51914)
				ModifyGraph/W=ShowPeaks rgb(Release1)=(31868,40092,51914)
				ModifyGraph/W=ShowPeaks rgb(Release2)=(31868,40092,51914)
				ModifyGraph/W=ShowPeaks mode(WA_PeakCentersY)=3
				ModifyGraph/W=ShowPeaks marker(WA_PeakCentersY)=19
			endif
		endif
	endif
	if( peaksFound < 1 )
		DoAlert 0, "No Peaks found!"
	endif
	return peaksFound

End



/////////////////////////////////////////////////////////////////////////////////////////////////////////////////////////////////////////////////////////////////////////////////////////////////
Function/C EstPeakNoiseAndSmfact(w,pBegin,pEnd)
	Wave w
	Variable pBegin,pEnd

	if (abs(pBegin-pEnd) < 21)			// 21 is pretty arbitrary; this is intended to avoid trying to apply this test to unreasonably small waves. Even a 10-point wave is probably a mistake: a fit coefficient wave was selected by mistake or something.
		return cmplx(0,0)
	endif
```

```
	if( pBegin > pEnd )
		Variable tmp = pBegin
		pBegin = pEnd
		pEnd = tmp
	endif

	pBegin = max(pBegin,0)
	pEnd = min(pEnd,numpnts(w)-1)

	if (pEnd - pBegin < 150)
		Variable resampleFactor = max(2, ceil(150/(pEnd - pBegin)))
		Duplicate/FREE w, resampledY
		Resample/UP=(resampleFactor)/WINF=None resampledY
		Wave w = resampledY
		pBegin *= resampleFactor
		pEnd *= resampleFactor
	endif

	// ST: prepare data folder and differentiated data waves.
	NewDataFolder/S/O AutoFindPeaksTemp
	Duplicate/O/R=[pBegin,pEnd] w, w_temp
	Differentiate w_temp
	Duplicate/O w_temp, w_temp_OrigDif

	// ST: estimate noise level and S/N ratio from data histogram and its cumulative
distribution function (CDF).
	Variable Histpnts = 999
	Make/O/N=(Histpnts+1) DataHistogram
	Histogram/B=1 w_temp, DataHistogram
	Duplicate/O DataHistogram, DataHistogram_Int
	Integrate DataHistogram_Int
```

```
	// ST: extract the width of the CDF around the center which should be an indication of the noise.
	FindLevel/Q DataHistogram_Int, (0.5 - 0.1) * DataHistogram_Int[Histpnts]
	Variable x0 = V_LevelX
	FindLevel/Q DataHistogram_Int, (0.5 + 0.1) * DataHistogram_Int[Histpnts]
	Variable x1 = V_LevelX
	Variable NoiseLevel			= abs(2*(x1-x0)*deltax(w))
	Variable SignalToNoise		= (pnt2x(DataHistogram_Int, Histpnts)-pnt2x(DataHistogram_Int, 0))/(x1-x0)

	// ST: the maximum useful smoothing factor depends on the size of the input wave.
	Variable maxSmoothFactor= max(2, (pEnd-pBegin+1)/20)

	// ST: create a wave with succesively increasing smoothing factors (not used).
	Variable nMaxSF=2* ceil(sqrt(maxSmoothFactor))
	Make/O/N=(nMaxSF) w_Smoothed_SNratio = 0, w_SmoothFactor = round((p/2)^2)

	// ST: another method to create a wave with succesively increasing smoothing factors. This overrides the ones above.
	Variable nLinFactors, nSpacedFactors

	if( maxSmoothFactor < 20 )	// ST: smoothing factors increase only linearily.
		nSpacedFactors = 0
		nLinFactors = maxSmoothFactor
	else					// ST: smoothing factors increase linearily for the first 10 point, then quadratic for the next 20 points.
		nLinFactors = 10
		nSpacedFactors = 20
	endif

	nMaxSF = nLinFactors + nSpacedFactors
	Make/O/N=(nMaxSF) w_Smoothed_SNratio = 0, w_SmoothFactor = p+1
```

```
	if( nSpacedFactors > 0 )		// ST: the quadratic part will be scaled to end at the maxSmoothFactor value.
		Variable accelerator = (maxSmoothFactor-nLinFactors)/nSpacedFactors^2
		w_SmoothFactor[nLinFactors,*]= ceil(nLinFactors + accelerator * (p - nLinFactors + 1)^2)
	endif


	// ST: successively increase the smoothing factor and observe how the S/N ratio changes as the noise (and then the signal) is suppressed. Want to find optimal compromise here.
	Variable i = 1,imax = min(nMaxSF, numpnts(w_SmoothFactor))
	w_Smoothed_SNratio[0] = SignalToNoise				// ST: the first (unsmoothed) value is already done above.
	do
		Duplicate/O w_temp_OrigDif, w_temp
		Smooth/E=2/B=3 2*w_SmoothFactor[i]+1, w_temp
		Histogram/B=1 w_temp, DataHistogram
		Duplicate/O DataHistogram, DataHistogram_Int
		Integrate DataHistogram_Int


		FindLevel/Q DataHistogram_Int, (0.5 - 0.1) * DataHistogram_Int[Histpnts]
		x0 = V_LevelX
		FindLevel/Q DataHistogram_Int, (0.5 + 0.1) * DataHistogram_Int[Histpnts]
		x1 = V_LevelX
		SignalToNoise = (pnt2x(DataHistogram_Int, Histpnts)-pnt2x(DataHistogram_Int, 0))/(x1-x0)
		w_Smoothed_SNratio[i]= SignalToNoise			// ST: next S/N ratio result
		i+=1
	while(i<imax)
	WaveTransform zapNaNs w_Smoothed_SNratio			// ST: 200806 - get rid of any NaN values
	WaveStats/Q/R=[2,] w_Smoothed_SNratio
	Variable SmoothFactor = maxSmoothFactor
	if (V_maxloc > -1)							// ST: 200806 - make sure there is any value at all
```

```
		SmoothFactor = w_SmoothFactor[V_maxloc]			// ST: maximal S/N ratio achieved here
	endif


	// added heuristics
	// Variable SmoothFactorwpd=0
	do
		if( w_Smoothed_SNratio[V_maxloc] < 100 )		// ST: S/N ratio somewhat too low => try to estimate the right smoothing factor to find a peak.
			i=0
			do
				Variable findPeaksReturn = AutoFindPeaks(w,pBegin,pEnd,noiselevel*10,w_SmoothFactor[i],1)
				//Variable findPeaksReturnOrig = AutoFindPeaksOriginal(w,pBegin,pEnd,noiselevel*10,w_SmoothFactor[i],1)
				//Variable findPeaksReturnNew = AutoFindPeaksNew(w,pBegin,pEnd,noiselevel*10,w_SmoothFactor[i],1)
				//print "Smooth Factor:", w_SmoothFactor[i], "Original:", findPeaksReturnOrig, "New:", findPeaksReturnNew


				//if( findPeaksReturnOrig > 0 )
				//if( findPeaksReturnNew > 0 )
				if( findPeaksReturn > 0 )				// ST: successfully found at least one peak at the current smoothing level.
					Wave wpd= W_AutoPeakInfo
					//SmoothFactorwpd = floor(wpd[0][1]/3)
					SmoothFactor = round(wpd[0][1]/3)	// ST: take around 1/3 of the width of the first found peak.
					//print "TRIAL FIND",wpd[0][1],SmoothFactor,i,w_SmoothFactor[i],w_Smoothed_SNratio[V_maxloc],w_Smoothed_SNratio[i]
					break;
				endif
				i+=1
			while(i<imax)
```

```
				if( findPeaksReturn > 0 )
					break
				endif
			endif
			// If really low snr and couldn't find a principal peak, force high smooth factors
			if( w_Smoothed_SNratio[V_maxloc] < 20 )
				SmoothFactor= maxSmoothFactor
				break
			endif
			if( w_Smoothed_SNratio[V_maxloc] < 30 )
				SmoothFactor= round(maxSmoothFactor/4)
				break
			endif
			if( w_Smoothed_SNratio[V_maxloc] < 50 )
				SmoothFactor= round(maxSmoothFactor/6)
				break
			endif
		while(0)

		if( SmoothFactor < 2 )
			SmoothFactor = 2
		endif
		//SmoothFactor = max(SmoothFactor, SmoothFactorwpd)

		KillDataFolder :
		//print noiselevel, SmoothFactor

		return cmplx(noiselevel,SmoothFactor)
end
//////////////////////////////////////////////////////////////////////////////////////////////////////////////////////////////////////////////////////
///////////////////////////////////////////////////////////////////////////
```

```
Function AutoFindPeaks(w,pBegin,pEnd,noiseEst,smFact,maxPeaks)
	Wave w
	Variable pBegin,pEnd
	Variable noiseEst,smFact
	Variable maxPeaks

	NVAR/Z doOriginal = root:V_PeakAutoFindClassic
	if ( (!NVAR_Exists(doOriginal)) || (doOriginal == 0) )
		return AutoFindPeaksNew(w,pBegin,pEnd,noiseEst,smFact,maxPeaks)
	else
		return AutoFindPeaksOriginal(w,pBegin,pEnd,noiseEst,smFact,maxPeaks)
	endif
end

static constant numPeakInfoColumns = 5			// JW 071031
Function AutoFindPeaksOriginal(w,pBegin,pEnd,noiseEst,smFact,maxPeaks)
	Wave w
	Variable pBegin,pEnd
	Variable noiseEst,smFact
	Variable maxPeaks

	if( pBegin > pEnd )
		Variable tmp= pBegin
		pBegin= pEnd
		pEnd= tmp
	endif

	Make/O/N=(0,numPeakInfoColumns) W_AutoPeakInfo

	NewDataFolder/S/O afpTemp2
	Duplicate/O/R=[pBegin,pEnd] w,wtmp1
```

```
	SetScale/P x,0,1,wtmp1				// we work in point numbers here

//	Duplicate/O wtmp1,wtmp2
	Smooth/B=3 smFact, wtmp1			// for peak amp determination

//Duplicate/O wtmp1, root:smooth1

	Duplicate/O wtmp1,wtmp2
//Duplicate/O wtmp1, root:debugDif

	Differentiate wtmp2
	Smooth/E=2/B=3 2*smFact, wtmp2
//Duplicate/O wtmp2, root:difsmooth2
	Differentiate wtmp2
	Smooth/E=2/B=3 2*smFact, wtmp2
//Duplicate/O wtmp2, root:dif2smooth3
	Duplicate/O wtmp2,wtmp3			// we mung one copy and need an unmunged version also

	Variable avgWidth=0			// for width not too far from average width criteria

	Variable i=0,peakNum=0,numBadPeaks=0
	do
		WaveStats/Q wtmp2
		Variable x0= V_minloc		// really, need to determine if + or - peaks and use right one

		if( V_min>=0 )
			break
		endif
```

```
		FindLevel/Q/R=[x0,] wtmp3,0
		Variable xr= V_LevelX
		if( V_Flag!=0 )
			xr= numpnts(wtmp3)-1
		endif
		FindLevel/Q/R=[x0,0] wtmp3,0			// note search is from right to left
		Variable xl= V_LevelX
		if( V_Flag!=0 )
			xl= 0
		endif
		wtmp2[xl-1,xr+1]=NaN						// don't find
this peak again

		Variable widthEst
		Variable rightWidthEst, leftWidthEst, leftWidthFraction		// JW 071031
		do
			if( (x0-xl) < 1 )
				widthEst= xr-x0					// if up against the
left edge, use right width
				break
			endif
			if( (xr-x0) < 1 )
				widthEst= x0-xl					// similar for right
edge
				break
			endif
			Variable ratio= (xr-x0)/ (x0-xl)	// right width/left width
			if( (ratio < 0.5) || (ratio>2) )
				widthEst=2* min(xr-x0, x0-xl)	// take smaller of widths if one is
much larger
				break
			endif
			widthEst= xr-xl
```

```
		while(0)

		rightWidthEst = xr-x0					// JW 071031
		leftWidthEst = x0-xl					// JW 071031
		leftWidthFraction = leftWidthEst/widthEst	// JW 071031

		if( !(widthEst>3) )					// this probably will neverhappen but if it did, we are probably out of real peaks
			break
		endif

		Variable impulseWidth= 2*(2*smFact+1)
		if( widthEst > 1.3*impulseWidth )
			widthEst= sqrt(widthEst^2 - impulseWidth^2)
		else
			widthEst= widthEst/2
		endif
		leftWidthEst = widthEst*leftWidthFraction
		rightWidthEst = widthEst - leftWidthEst

		Variable yl= wtmp1[xl], y0= wtmp1[x0], yr= wtmp1[xr]
		Variable bl0= ((yr-yl)/(xr-xl)*(x0-xl))+yl		// y at x0 for line between left and right inflection points

		Variable heightEst= 2*(y0-bl0)

		Variable avgNoiseEst= noiseEst/(1.35*sqrt(2*smFact+1))
		Variable minH= avgNoiseEst*8

		// throw in an additional penalty if width is far away from the average
		if( avgWidth>0 )
			minH *= sqrt( (widthEst/avgWidth)^2 + (avgWidth/widthEst)^2 )
```

```
			endif
			if( heightEst >  minH )
				Redimension/N=(peakNum+1,numPeakInfoColumns) W_AutoPeakInfo
				avgWidth= (avgWidth*peakNum+widthEst)/(peakNum+1)

	W_AutoPeakInfo[peakNum]={{x0+pBegin},{widthEst},{heightEst},{leftWidthEst},{rightWid
thEst}}
				peakNum+=1
			else
				if( peakNum == 0 )
					break					// if very first peak is bad,
then give up
				endif
				numBadPeaks += 1
				if( numBadPeaks > 3 )
					break
				endif
			endif
	while(peakNum<maxPeaks)
	KillDataFolder :

	return	peakNum
end
```

// The AutoFindPeaks functions make a temporary data folder and then kills it. This function depends on that to get rid of some temporary waves.

// Creates a wave "results" in the current data folder. That wave contains the following data for peaks in each row:

// col		data

// 0			mid-peak location. That is, the negative peak resulting from the minimum in 2nd deriv at the center of a peak.

// 1			Y value at mid-peak location.

// 2			left peak location. That is, the location of the positive peak in the 2nd deriv on the left side of the peak. If only partial peak, may be zero and inaccurate.

```
//	3				Y value at left peak location.
// 4			right peak location. That is, the location of the positive peak in the 2nd deriv on the right side of the peak. If only partial peak, may be numpnts(win)-1 and inaccurate.
//	5				Y value at right peak location.

/////////////////////////////////////////////////////////////////////////////////////////////////////////////////////////////////////////////////////////////////////////////////////////////////

Function findPeaksIn2ndDeriv(win)
	Wave win

	Make/N=(100,7)/O results
	Variable startP = 0
	Variable numPeak=0
	Variable lowBad = 0
	Variable highBad = 0

	findpeak/B=1/N/P/R=[startP,]/Q win
	results[numPeak][0]=V_PeakLoc
	results[numPeak][1]=V_PeakVal
	if (V_flag)
		// couldn't find even one low point???
		Redimension/N=0 results
		return -1
	endif
	findpeak/B=1/P/R=[V_PeakLoc,0]/Q win
	if (V_flag)
		results[numPeak][2]=0
		results[numPeak][3]=win[0]
//		lowBad = 1
```

```
else
	results[numPeak][2]=V_PeakLoc
	results[numPeak][3]=V_PeakVal
endif
findpeak/B=1/P/R=[results[numPeak][0],]/Q win
results[numPeak][4]=V_PeakLoc
results[numPeak][5]=V_PeakVal
if (V_flag)
	// Couldn't find the other side of the first peak
	Redimension/N=0 results
	return -1
endif
if (lowBad)
	startP = V_PeakLoc
endif

Variable leftPeakLoc, leftPeakVal
Variable midPeakLoc, midPeakVal
Variable rightPeakLoc, rightPeakVal
do
	findpeak/B=1/N/P/R=[startP,]/Q win
	midPeakLoc=V_PeakLoc
	midPeakVal=V_PeakVal
	if (V_flag)
		break;
	endif
	findpeak/B=1/P/R=[V_PeakLoc,0]/Q win
	leftPeakLoc=V_PeakLoc
	leftPeakVal=V_PeakVal
	if (V_flag)
		leftPeakLoc = 0
		leftPeakVal = win[0]
```

```
		endif
		findpeak/B=1/P/R=[midPeakLoc,]/Q win
		rightPeakLoc=V_PeakLoc
		rightPeakVal=V_PeakVal
		Variable rightPeakBad = 0
		if (V_flag)
			rightPeakLoc = numpnts(win)-1
			rightPeakVal = win[rightPeakLoc]
			rightPeakBad = 1
		endif

		results[numPeak][0]=midPeakLoc
		results[numPeak][1]=midPeakVal
		results[numPeak][2]=leftPeakLoc
		results[numPeak][3]=leftPeakVal
		results[numPeak][4]=rightPeakLoc
		results[numPeak][5]=rightPeakVal
		results[numPeak][6]=min(abs(midPeakVal - leftPeakVal), abs(midPeakVal - 
rightPeakVal))

		startP=V_PeakLoc
		numPeak += 1
		if (numPeak >= DimSize(results, 0))
			Redimension/N=(numPeak+100, -1) results
		endif
		if (rightPeakBad)
			break
		endif
	while(1)

	// JW 140401 renamed "sortwave" to "PeakAutoFindSortWave" to avoid a conflict with a 
function name in Neuromatic.
```

```
	if (numpeak == 0)
		Redimension/N=0 results
		return -1
	else
		Redimension/N=(numPeak, -1) results
		Make/O/N=(numPeak)/FREE PeakAutoFindSortWave
		PeakAutoFindSortWave = results[p][6]
		MakeIndex/R PeakAutoFindSortWave, PeakAutoFindSortWave
		Duplicate/O results, resultscopy
		//Duplicate/O results, root:resultscopy
		//Wave resultscopy = root:resultscopy
		results = resultscopy[PeakAutoFindSortWave[p]][q]
	endif
	//Duplicate/O results, root:resultsSorted
end


// for debugging
//Function PutLinesOnGraph(win, graph, numpeaks)
//	Wave win
//	String Graph
//	Variable numpeaks
//
//	Variable numRows = DimSize(win, 0)
//	Variable i
//
//	SetDrawLayer/W=$Graph/K progBack
//	for (i = 0; i < numpeaks; i += 1)
//		SetDrawEnv/W=$Graph
fillfgc=(50000,50000,50000),linethick=1,ycoord=prel,xcoord=bottom
//		DrawRect/W=$Graph win[i][2], 1-i*.01, win[i][4], 0+(numpeaks-i)*.01
//		SetDrawEnv/W=$Graph
linefgc=(30000,30000,30000),ycoord=prel,xcoord=bottom
```

```
//			DrawLine/W=$Graph win[i][0], 0, win[i][0], 1
//			SetDrawEnv/W=$Graph textxjust=1,ycoord=prel,xcoord=bottom
//			DrawText/W=$Graph win[i][0], 1-i*.01, num2str(i)
//		endfor
//end


/////////////////////////////////////////////////////////////////////////////////////////////////////////////////////////
////////////////////////////////////////////////////////////////


Function AutoFindPeaksNew(w,pBegin,pEnd,noiseEst,smFact,maxPeaks)
	Wave w
	Variable pBegin,pEnd
	Variable noiseEst,smFact
	Variable maxPeaks

	if( pBegin > pEnd )
		Variable tmp= pBegin
		pBegin= pEnd
		pEnd= tmp
	endif

	Variable resampleFactor = 1
	if (pEnd - pBegin < 150)
		// JW 150930 bail on a number of points that is unreasonably small. The up-sampling algorithm needs SOME information, and I think up-sampling
		// is already kind of heroic
		if (pEnd - pBegin < 21)
			Make/O/N=(0,numPeakInfoColumns) W_AutoPeakInfo = 0
			return 0
		endif
```

```
			resampleFactor = max(2, ceil(150/(pEnd - pBegin)))
			Duplicate/FREE w, resampledY
			Resample/UP=(resampleFactor)/WINF=None resampledY
			Wave w = resampledY
			pBegin *= resampleFactor
			pEnd *= resampleFactor
		endif

		Make/O/N=(0,numPeakInfoColumns) W_AutoPeakInfo

		NewDataFolder/S/O afpTemp2
		Duplicate/O/R=[pBegin,pEnd] w,wtmp1
		SetScale/P x,0,1,wtmp1						// we work in point numbers
here

		Smooth/B=3 smFact, wtmp1					// for peak amp determination

//Duplicate/O wtmp1, root:smooth1

		Duplicate/O wtmp1,wtmp2
//Duplicate/O wtmp1, root:debugDif

		Differentiate wtmp2
		Smooth/E=2/B=3 2*smFact, wtmp2
//Duplicate/O wtmp2, root:difsmooth2
		Differentiate wtmp2
		Smooth/E=2/B=3 2*smFact, wtmp2
//Duplicate/O wtmp2, root:dif2smooth3
		Duplicate/O wtmp2,wtmp3					// we mung one copy and need an
unmunged version also

		findPeaksIn2ndDeriv(wtmp2)
```

```
//PutLinesOnGraph(results, "Graph7", DimSize(results, 0))
	Wave Results
//print GetWavesDataFolder(results, 2)
	maxPeaks = min(maxPeaks, DimSize(results, 0))
	Variable nRows = DimSize(results, 0)

	Variable avgWidth=0				// for width not too far from average width criteria

	Variable i=0,peakNum=0,numBadPeaks=0
	for (i = 0; i < nRows; i += 1)
		Variable x0= Results[i][0]
		Variable xr= Results[i][4]
		Variable xl= Results[i][2]

		Variable widthEst
		Variable rightWidthEst, leftWidthEst, leftWidthFraction		// JW 071031
		do
			if( (x0-xl) < 2 )
				widthEst= xr-x0				// if up against the left edge, use right width
				break
			endif
			if( (xr-x0) < 2 )
				widthEst= x0-xl				// similar for right edge
				break
			endif
			Variable ratio= (xr-x0)/ (x0-xl)	// right width/left width
			if( (ratio < 0.5) || (ratio>2) )
				widthEst=2* min(xr-x0, x0-xl)	// take smaller of widths if one is much larger
				break
```

```
			endif
			widthEst= xr-xl
		while(0)

		rightWidthEst = xr-x0						// JW 071031
		leftWidthEst = x0-xl						// JW 071031
		leftWidthFraction = leftWidthEst/widthEst	// JW 071031

		if( !(widthEst>3) )						// this probably will neverhappen but if it did, we are probably out of real peaks
			break
		endif

		Variable impulseWidth= 2*(2*smFact+1)
		if( widthEst > 1.3*impulseWidth )
			widthEst= sqrt(widthEst^2 - impulseWidth^2)
		else
			widthEst= widthEst/2
		endif
		widthEst /= sqrt(6)
		leftWidthEst = widthEst*leftWidthFraction
		rightWidthEst = widthEst - leftWidthEst

		Variable yl= wtmp1[xl], y0= wtmp1[x0], yr= wtmp1[xr]
		Variable bl0 = min(yr, yl)

		Variable heightEst= 1.3*(y0-bl0)
		if (heightEst < 0)
			continue
		endif

		Variable avgNoiseEst= noiseEst/(1.35*sqrt(2*smFact+1))
```

```
		Variable minH= avgNoiseEst*8
		Variable saveMinH = minH

		// throw in an additional penalty if width is far away from the average
		if( avgWidth>0 )
			minH *= sqrt( (widthEst/avgWidth)^2 + (avgWidth/widthEst)^2 )
		endif
//print "i=",i,"; point=", x0, "; original minH=",saveMinH,"; minH=",minH,"; heightEst=",heightEst, "; widthEst=", widthEst
		if( heightEst >  minH )
			Redimension/N=(peakNum+1,numPeakInfoColumns) W_AutoPeakInfo
			avgWidth= (avgWidth*peakNum+widthEst)/(peakNum+1)
	W_AutoPeakInfo[peakNum]={{x0+pBegin},{widthEst},{heightEst},{leftWidthEst},{rightWidthEst}}
			peakNum+=1
		else
			if( peakNum == 0 )
				break					// if very first peak is bad, then give up
			endif
			numBadPeaks += 1
			if( numBadPeaks > 3 )
				break
			endif
		endif
		if(peakNum>=maxPeaks)
			break;
		endif
	endfor
	KillDataFolder :

	if (resampleFactor > 1)
```

```
		W_AutoPeakInfo[][0,1] = round(W_AutoPeakInfo[p][q]/resampleFactor)
		W_AutoPeakInfo[][3,4] = round(W_AutoPeakInfo[p][q]/resampleFactor)
	endif

	return	peakNum
end

////////////////////////////////////////////////////////////////////////////////////////////////////////////////////////////////
/////////////////////////////////////////////////////////////////

Function AdjustAutoPeakInfoForX(wpi,yData,xData)
	Wave wpi,yData
	WAVE/Z xData

	Variable imax= DimSize(wpi,0),i=0
	do
		Variable side1, side2, reversed
		if( WaveExists(xData) )
			reversed = xData[0] > xData[numpnts(xData)-1]
			Variable p0= wpi[i][0]
			Variable pw= wpi[i][1]/2
			wpi[i][0]=xData[p0]
			wpi[i][1]= abs(xData[p0+pw] - xData[p0-pw])
			Variable pLw = wpi[i][3]
			Variable lpoint = p0-pLw
			lpoint = min(max(0, lpoint), numpnts(xData)-1)
			side1= abs(xData[p0] - xData[lpoint])
			Variable pRw = wpi[i][4]
			Variable rpoint = min(max(0, p0-pRw), numpnts(xData)-1)
			side2= abs(xData[rpoint] - xData[p0])
		else
			reversed = deltaX(yData) < 0
```

```
				wpi[i][0]=pnt2x(yData,wpi[i][0])
				wpi[i][1]=  abs(wpi[i][1]*deltax(yData))
				// asymmetry info
				side1 = abs(wpi[i][3]*deltax(yData))
				side2 = abs(wpi[i][4]*deltax(yData))
			endif
			// JW 200401 since the peak picker works in points, not in X values, the
asymmetry
			// measure is reversed if the X scaling is negative
			if (reversed)
				wpi[i][4]=  side1
				wpi[i][3]=  side2
			else
				wpi[i][3]=  side1
				wpi[i][4]=  side2
			endif
			i+=1
		while(i<imax)
end

Function TrimAmpAutoPeakInfo(wpi,gMinPeakFraction)
		Wave wpi
		Variable gMinPeakFraction

		Variable i= DimSize(wpi,0)-1							// index of last row
		Variable ymin= wpi[0][2]*gMinPeakFraction	// user want peaks to be bigger than this

		do
			if( wpi[i][2] < ymin )
				DeletePoints i,i,wpi
			endif
			i -= 1
```

```
	while(i>0)

	return DimSize(wpi,0)
end

////////////////////////////////////////////////////////////////////////////////////////////////////////////////////////////////////////////////////////////////////

////////////////////////////////////////////////////////////////////////////////////////////////////////////////////////////////////////////////////////////////////
```

```
//Function AutomaticallyFindPeaks()

	String wname, xdata="_calculated_"
	Variable maxPeaks=30, minPeakPercent=12.5
	Prompt wname, "Peak Wave", popup,
WaveList("*",";","DIMS:1,TEXT:0,CMPLX:0")+"_none_;"
	Prompt xdata, "X values", popup, "_calculated_;"
	Prompt maxPeaks, "Maximum Peaks"
	Prompt minPeakPercent, "Minimum Peak Amplitude (% max)"
	DoPrompt "Automatically Find Peaks", wname, xdata, maxPeaks, minPeakPercent
	if( V_Flag != 0 )
		return 0		// user cancelled
	endif

	WAVE/Z w=$wname
	WAVE/Z wx=$xdata
	Variable pBegin=0, pEnd= numpnts(w)-1
	Variable/C estimates= EstPeakNoiseAndSmfact(w,pBegin, pEnd)
	Variable noiselevel=real(estimates)
	Variable smoothingFactor=imag(estimates)
```

```
	AutoFindPeaksWorker(w, wx, pBegin, pEnd, maxPeaks, minPeakPercent, noiseLevel, smoothingFactor)
end
```

//// Marcos and function to analyce isolated minis

```
Menu "Macros"
	Submenu "Isolate the release"
		"10 Hz",Isolate10()
		"50 Hz",Isolate50()
		"100 Hz",Isolate100()
		"200 Hz",Isolate200()
		"300 Hz",Isolate300()
		"400 Hz",Isolate400()
	End
	Submenu "Kill the empty ones"
	"Do it", Kill()
	End

	Submenu "Fit it"
	"Do it", Fitoftau()
	End
End
```

```
Function Isolate10()
Wave Release0,Release1,Release2

Display root:Asynchron_release:release10Hz:Release:Release2:release2
ModifyGraph rgb=(31868,40092,51914)
Display root:Asynchron_release:release10Hz:Release:Release1:release1
ModifyGraph rgb=(31868,40092,51914)
Display root:Asynchron_release:release10Hz:Release:Release0:release0
ModifyGraph rgb=(31868,40092,51914)

end

Function Isolate50()
Wave Release0,Release1,Release2

Display root:Asynchron_release:release50Hz:Release:Release2:release2
ModifyGraph rgb=(31868,40092,51914)
Display root:Asynchron_release:release50Hz:Release:Release1:release1
ModifyGraph rgb=(31868,40092,51914)
Display root:Asynchron_release:release50Hz:Release:Release0:release0
ModifyGraph rgb=(31868,40092,51914)

end

Function Isolate100()
Wave Release0,Release1,Release2

Display root:Asynchron_release:release100Hz:Release:Release2:release2
ModifyGraph rgb=(31868,40092,51914)
Display root:Asynchron_release:release100Hz:Release:Release1:release1
ModifyGraph rgb=(31868,40092,51914)
Display root:Asynchron_release:release100Hz:Release:Release0:release0
```

```
ModifyGraph rgb=(31868,40092,51914)

SetDataFolder root:Asynchron_release:release100Hz:
end

Function Isolate200()
Wave Release0,Release1,Release2

Display root:Asynchron_release:release200Hz:Release:Release2:release2
ModifyGraph rgb=(31868,40092,51914)
Display root:Asynchron_release:release200Hz:Release:Release1:release1
ModifyGraph rgb=(31868,40092,51914)
Display root:Asynchron_release:release200Hz:Release:Release0:release0
ModifyGraph rgb=(31868,40092,51914)

end

Function Isolate300()
Wave Release0,Release1,Release2

Display root:Asynchron_release:release300Hz:Release:Release2:release2
ModifyGraph rgb=(31868,40092,51914)
Display root:Asynchron_release:release300Hz:Release:Release1:release1
ModifyGraph rgb=(31868,40092,51914)
Display root:Asynchron_release:release300Hz:Release:Release0:release0
ModifyGraph rgb=(31868,40092,51914)

end

Function Isolate400()
Wave Release0,Release1,Release2
```

```
	Display root:Asynchron_release:release400Hz:Release:Release2:release2
	ModifyGraph rgb=(31868,40092,51914)
	Display root:Asynchron_release:release400Hz:Release:Release1:release1
	ModifyGraph rgb=(31868,40092,51914)
	Display root:Asynchron_release:release400Hz:Release:Release0:release0
	ModifyGraph rgb=(31868,40092,51914)

	end

//------------------------------------------------------------------------------------//

Menu "TracePopup"
  SubMenu "Extract the release"
    "Extract Rep0", /Q, GettheEPCSs_Rep1()
    "Extract Rep1", /Q, GettheEPCSs_Rep2()
    "Extract Rep2", /Q, GettheEPCSs_Rep3()
  End
End

Function GettheEPCSs_Rep1()
Variable k
Wave PeakPoints, WA_PeakCentersX
Variable Beginning, Ending
String Original_Wave
Original_Wave="release0"


//Turn the timepoints of peak into poitns
Duplicate WA_PeakCentersX, PeakPoints
PeakPoints/= 2e-05 // use the right sample rate here!!

for(k=0;k<numpnts(PeakPoints);k+=1)
```

```
	Beginning=PeakPoints[k]-5
	Ending=PeakPoints[k]+72
	Duplicate/R= [Beginning,Ending] $Original_Wave,$"peak"+num2str(k)
	Wave New_Wave=$"peak"+num2str(k)

endfor



end


Function GettheEPCSs_Rep2()
Variable k
Wave PeakPoints, WA_PeakCentersX
Variable Beginning, Ending
String Original_Wave
Original_Wave="release1"

//Turn the timepoints of peak into poitns
Duplicate WA_PeakCentersX, PeakPoints
PeakPoints/= 2e-05 // use the right sample rate here!!

for(k=0;k<numpnts(PeakPoints);k+=1)
	Beginning=PeakPoints[k]-5
	Ending=PeakPoints[k]+72
	Duplicate/R= [Beginning,Ending] $Original_Wave,$"peak"+num2str(k)
	Wave New_Wave=$"peak"+num2str(k)

endfor



end
```

```
Function GettheEPCSs_Rep3()
Variable k
Wave PeakPoints, WA_PeakCentersX
Variable Beginning, Ending
String Original_Wave
Original_Wave="release2"


//Turn the timepoints of peak into poitns
Duplicate WA_PeakCentersX, PeakPoints
PeakPoints/= 2e-05 // use the right sample rate here!!

for(k=0;k<numpnts(PeakPoints);k+=1)
	Beginning=PeakPoints[k]-5
	Ending=PeakPoints[k]+72
	Duplicate/R= [Beginning,Ending] $Original_Wave,$"peak"+num2str(k)
	Wave New_Wave=$"peak"+num2str(k)

endfor


end


//--------------------------------------------------------------------------------------------//



Function selectPSCs()

	variable n
	string my_list = wavelist("peak*", ";", (""))
```

```
        for (n=0; n < itemsinlist(my_list); n+=1)
                display $(stringfromlist(n,my_list))
        endfor
end

//-------------------------------------------------------------------------------------------//


Function Kill()
variable n
        string my_list = wavelist("peak*", ";", (""))

                for (n=0; n < itemsinlist(my_list); n+=1)
                if (numpnts($(stringfromlist(n,my_list)))==1)
                killwaves $(stringfromlist(n,my_list))
                endif

                endfor

        end


//-------------------------------------------------------------------------------------------//

Function Fitoftau()
variable n

string my_list
        //make/o/N=(0) nofit
```

```
	my_list= wavelist("peak*", ";", (""))
//variable fitend=V_maxloc
	make/o/n=(itemsinlist(my_list)) minitaus
	make/o/t/n=(itemsinlist(my_list)) mininames

		for (n=0; n < itemsinlist(my_list); n+=1)
			Wavestats $(stringfromlist(n,my_list))
			CurveFit exp_XOffset  $stringfromlist(n,my_list)
[V_maxRowLoc,V_endRow] /D
			wave W_Coef=W_Coef
			minitaus[n]=W_Coef[2]
			mininames[n]=stringfromlist(n,my_list)

		endfor
end



//-----------------------------------------------------------------------------------------//



//Function test()
variable n
	string my_list=wavelist("peak*", ";", (""))

		for (n=0; n < itemsinlist(my_list); n+=1)
		Display/B $(stringfromlist(n,my_list))
		SetAxis/A
		doalert 2, "keep_or_kill?"
			if (V_Flag==1)
			//if (numpnts($(stringfromlist(n,my_list)))==1)
```

```
				SetAxis/A
				killwaves $(stringfromlist(n,my_list))
				killwindow $(stringfromlist(n,my_list))
			elseif (V_Flag==2)
				killwindow $(stringfromlist(n,my_list))
			elseif (V_Flag==3)
				killwindow $(stringfromlist(n,my_list))
			abort
		endif

		endfor

end
```
