# Supplementary material for: Non-calyceal inputs gate the timing of calyx of Held evoked MNTB output
Source: Commun Biol. 2026 May 22;9:697. doi: 10.1038/s42003-026-10321-w (PMC13197447; doi:10.1038/s42003-026-10321-w)
Supplement: Supplementary file 4 — Supplementary code [file 42003_2026_10321_MOESM4_ESM.pdf]

```
#pragma TextEncoding = "Windows-1252"
#pragma rtGlobals=3		// Use modern global access method and strict wave access.

Menu "Basic Analysis"
	"VC_analysis", VC_step()
	"CC_analysis", CC_step()
	"IV", IV()
	"Ramp", Ramp()
	"Rheobase", Rheobase()
end

Menu "Train Analysis"
	"Trains",Trains()
	"Trains_last_20", Trains_last_20()
end
```

```
Function VC_step()

Variable sample_rate, start_stim, stim_volt

sample_rate=1e-5
start_stim=0.1
stim_volt=5

prompt start_stim,"Stimulus start at [ms]: "
prompt sample_rate,"Sample rate: "
prompt stim_volt,"VC step [mV]: "
Doprompt "VC stepper",start_stim,sample_rate,stim_volt
if ( V_Flag )
	return 0
endif
```

```
String Original_Waves

Original_Waves=WaveList("*",";",("WIN:"))

Variable n,ind


Make/O/N=7 VC_prop

Make/O/T/N=7 VC_prop_leg={"I_leak [pA]","Rin [MOhm]","C_weighted_5 [pF]","C_mono_3
[pF]","tau_fast [ms]","tau_slow [ms]","tau_weighted [ms]"}


//Make an average wave named w_avg

for(n=0;n<itemsinlist(Original_Waves);n+=1)

	if(n==0)

		Duplicate/O $StringFromList(n,Original_Waves), VC_avg

	else

		Wave new_wave=$StringFromList(n,Original_Waves)

		VC_avg+=new_wave

	endif

endfor


VC_avg/=itemsinlist(Original_Waves)


// 1. Calculate I_Leak before stimulus


Variable I_Leak

WaveStats/Q/R=(start_stim-0.001,start_stim) VC_avg

I_Leak=V_avg

VC_prop[0]=I_Leak*1e12


// 2. Calculate Rin


Variable max_pnt_R, Rin

// find maximum point between 10 and 15 ms after stimulus
```

```
WaveStats/Q/R=(start_stim+0.01,start_stim+0.015) VC_avg

max_pnt_R=V_min

// Calculate the average current 1ms before and after the maximum point

WaveStats/Q/R=(max_pnt_R-0.001,max_pnt_R+0.001) VC_avg

// Calculate Rin (Ohms Law)

Rin=stim_volt/(I_Leak-V_avg)/1000000000

VC_prop[1]=Rin


// 3. Copy wave and zero it before stimulus

Duplicate/O VC_avg, VC_avg_zeroed

VC_avg_zeroed-=I_Leak


Display

AppendtoGraph VC_avg_zeroed

Make/O/N=5 W_Coef

Make/O/N=3 tau


//Find minimum deflection

Variable min_time

WaveStats/Q/R=(start_stim,start_stim+0.001) VC_avg_zeroed

min_time=V_minloc


// Add bi-exponential fit on VC stepper

ModifyGraph axisEnab(left)={0,0.45}

CurveFit dblexp_XOffset
VC_avg_zeroed[min_time/sample_rate+0,(min_time+0.01)/sample_rate+0] /D


//weighted decay

Variable t_weighed

t_weighed=(W_coef[2]*(-1*W_coef[1])+W_coef[4]*(-1*W_coef[3]))/((-1*W_coef[1])+(-
1*W_coef[3]))

VC_prop[5]=W_coef[4]*1000
```

```
VC_prop[6]=t_weighed*1000

// Create a mono-exponential trace
Make/O/N=3 W_coef_mono
W_coef_mono[0]=W_coef[0]
W_coef_mono[1]=W_coef[1]+W_coef[3]
W_coef_mono[2]=W_coef[2] //tau_1
VC_prop[4]=W_coef_mono[2]*1000

Duplicate/O VC_avg_zeroed, VC_avg_mono

AppendtoGraph VC_avg_mono
VC_avg_mono[min_time/sample_rate+0,(min_time+0.01)/sample_rate+0]=W_coef_mono[0]+W_coef_mono[1]*exp(-(x-(min_time))/W_coef_mono[2])
ModifyGraph rgb(VC_avg_mono)=(1,16019,65535)

// Integrate both graphs (original and new)
Integrate VC_avg_zeroed/D=Original_Integration;DelayUpdate
Integrate VC_avg_mono/D=Mono_Integration;DelayUpdate

// Display Integrations
AppendToGraph/L=L_Int Original_Integration, Mono_Integration
ModifyGraph axisEnab(L_Int)={0.55,1}
ModifyGraph rgb(Mono_Integration)=(1,12815,52428)
ModifyGraph fSize=11,axThick=1.2,btLen=3
ModifyGraph freePos(L_Int)=0

//ModifyGraph width=200, height=400

// Calculate C
variable C_original_fast_5, C_mono_fast_3
```

C_original_fast_5=(Original_Integration(start_stim)-Original_Integration(min_time+5*VC_prop[4]/1000))*1e12/(stim_volt/1000)

C_mono_fast_3=(Mono_Integration(start_stim)-Mono_Integration(min_time+3*W_coef_mono[2]))*1e12/(stim_volt/1000)

VC_prop[2]=C_original_fast_5

VC_prop[3]=C_mono_fast_3

Make/O/N=1 pnt_orig_x, pnt_mono_x, pnt_orig_y, pnt_mono_y, pnt_trace_orig_y, pnt_trace_mono_y

pnt_orig_x[0]=min_time+5*W_coef_mono[2]

pnt_mono_x[0]=min_time+3*W_coef_mono[2]

pnt_orig_y[0]=Original_Integration(min_time+5*W_coef_mono[2])

pnt_mono_y[0]=Mono_Integration(min_time+3*W_coef_mono[2])

pnt_trace_orig_y[0]=VC_avg_zeroed(min_time+5*W_coef_mono[2])

pnt_trace_mono_y[0]=VC_avg_mono(min_time+3*W_coef_mono[2])

AppendToGraph/L=L_Int pnt_orig_y vs pnt_orig_x

ModifyGraph mode(pnt_orig_y)=3,marker(pnt_orig_y)=8,opaque(pnt_orig_y)=1,rgb(pnt_orig_y)=(39321,1,1)

AppendToGraph/L=L_Int pnt_mono_y vs pnt_mono_x

ModifyGraph mode(pnt_mono_y)=4,marker(pnt_mono_y)=8,opaque(pnt_mono_y)=1,rgb(pnt_mono_y)=(1,9611,39321)

AppendToGraph pnt_trace_orig_y vs pnt_orig_x

ModifyGraph mode(pnt_trace_orig_y)=4,marker(pnt_trace_orig_y)=8,opaque(pnt_trace_orig_y)=1,rgb(pnt_trace_orig_y)=(39321,1,1)

AppendToGraph pnt_trace_mono_y vs pnt_mono_x

```
ModifyGraph
mode(pnt_trace_mono_y)=4,marker(pnt_trace_mono_y)=8,opaque(pnt_trace_mono_y)=1,rgb(p
nt_trace_mono_y)=(1,9611,39321)

SetAxis bottom start_stim-0.001,start_stim+0.005
ModifyGraph lblPos(L_Int)=50

SetWindow kwTopWin//, sizeLimit={0,350,280,500}
MoveWindow 0, 0, 280, 350

Edit VC_prop_leg, VC_prop
SetWindow kwTopWin//, sizeLimit={0,200,700,150}
MoveWindow 300, 0, 550, 200

end
```

```
Function CC_step()

Variable sample_rate, start_stim, start_dur, stim_pA

sample_rate=1e-5
start_stim=0.05
start_dur=0.3
stim_pA=5

prompt start_stim,"Stimulus start at [ms]: "
prompt start_dur, "Stimulus duration [ms]: "
prompt sample_rate,"Sample rate: "
```

```
prompt stim_pA,"CC step [pA]: "
Doprompt "CC stepper", start_stim, start_dur, sample_rate, stim_pA
if ( V_Flag )
	return 0
endif

String Original_Waves
Original_Waves=WaveList("*",";",("WIN:"))
Variable n

Make/O/T/N=7 cc_descr={"Erest [mV]","Rin_on [mOhm]","Rin_stst [mOhm]","tau [ms]","Ceff [pF]","sag [mV]","sag_norm"}
Make/O/N=7 cc_pass_prop
Make/O/N=1 Vmin


//Make an average wave named cc_avg
for(n=0;n<itemsinlist(Original_Waves);n+=1)
	if(n==0)
		Duplicate/O $StringFromList(n,Original_Waves), cc_avg
	else
		Wave new_wave=$StringFromList(n,Original_Waves)
		cc_avg+=new_wave
	endif
endfor
cc_avg/=itemsinlist(Original_Waves)

Display
AppendtoGraph cc_avg
ModifyGraph rgb=(0,0,0)

//Find Erest
```

```
WaveStats/Q/R=(start_stim-0.01, start_stim-10*sample_rate) cc_avg
cc_pass_prop[0]=V_avg*1000 //Erest

WaveStats/Q/R=(start_stim+start_dur-0.01, start_stim+start_dur) cc_avg
Variable Diff_stst=V_avg
cc_pass_prop[2]=(Diff_stst*1000-cc_pass_prop[0])/-stim_pA*1000 //Rin stst

WaveStats/Q/R=(start_stim, start_stim+start_dur) cc_avg
Variable min_defl=V_minloc
WaveStats/Q/R=(min_defl-0.0005, min_defl+0.0005) cc_avg
Variable Diff_on=V_avg
cc_pass_prop[1]=(Diff_on*1000-cc_pass_prop[0])/-stim_pA*1000 //Rin onset

//Find tau
Make/O/N=3 W_coef
CurveFit/Q exp_XOffset cc_avg(start_stim,min_defl)/D
cc_pass_prop[3]=W_coef[2]*1000

//Find Ceff
cc_pass_prop[4]=cc_pass_prop[3]/cc_pass_prop[1]*1000

// sag
cc_pass_prop[5]=(Diff_on-Diff_stst)
cc_pass_prop[6]=cc_pass_prop[5]/Diff_on
cc_pass_prop[5,6]*=1000

ModifyGraph lsize(fit_cc_avg)=2
ModifyGraph fSize=11,axThick=1.2,btLen=3

SetWindow kwTopWin
MoveWindow 0, 0, 450, 200
```

```
Edit cc_descr, cc_pass_prop
SetWindow kwTopWin
MoveWindow 460, 0, 730, 200

end
```

```
Function IV()

Variable start_stim, stim_dur, start_current, step_current, Level

start_stim=0.05
stim_dur=0.3
start_current=525
step_current=50
Level=-0.02

prompt start_stim,"Stimulus start at [s]: "
prompt stim_dur,"Stimulus duration [s]: "
prompt start_current,"Highest current [pA]: "
prompt step_current,"Step current [pA]: "
prompt Level,"Find AP Level [V]: "

Doprompt "IC",start_stim,stim_dur,start_current,step_current, Level
if ( V_Flag )
	return 0
endif
```

```
String Original_Waves

Original_Waves=WaveList("*",";",("WIN:"))

Variable n, k, m, l, first_idx



// Differenatiation


//for(n=0;n<itemsinlist(Original_Waves);n+=1)

//	Wave new_wave=$StringFromList(n,Original_Waves)

//	Differentiate new_wave/D=$"new_wave_"+num2str(n);DelayUpdate

//	if(n==0)

//		Display $"new_wave_"+num2str(n)

//	else

//		AppendToGraph $"new_wave_"+num2str(n)

//	endif

//endfor


Make/O/N=(itemsinlist(Original_Waves)) curr_steps, spike_num, min_curr, st_st, IV_FSL


for(n=0;n<itemsinlist(Original_Waves);n+=1)

	// Calculate the current

	curr_steps[n]=start_current-n*step_current

	// Find number of spikes

	FindLevels/Q/D=spikes_found/R=(start_stim,start_stim+stim_dur)
$StringFromList(n,Original_Waves), Level

	spike_num[n]=V_LevelsFound/2


	Make/O/N=(numpnts(spikes_found)/2) max_AP


	for(m=1;m<numpnts(spikes_found);m+=2)

		WaveStats/Q/R=(spikes_found[m-1],spikes_found[m])
$StringFromList(n,Original_Waves)
```

```
			if(m==1)
				IV_FSL[n]=V_maxloc-start_stim
			endif

			for(l=0;l<numpnts(IV_FSL);l+=1)
				if(IV_FSL[l]==0)
					IV_FSL[l]=NaN
				endif
			endfor
			max_AP[(m-1)/2]=V_maxloc
			Make/O/N=(numpnts(max_AP)-1) ISI
			for(k=1;k<numpnts(max_AP);k+=1)
				ISI[k-1]=max_AP[k]-max_AP[k-1]
			endfor
			if(n<10)
				Duplicate/O ISI, $"ISI_0"+num2str(n)
			else
				Duplicate/O ISI, $"ISI_"+num2str(n)
			endif
		endfor

		String ISI_waves
		ISI_waves=WaveList("ISI_*",";",(""))

		Duplicate/O spikes_found, $"spikes_found_"+num2str(n)

		// Calculate minimum and steady state
		WaveStats/Q/R=(start_stim,start_stim+0.1) $StringFromList(n,Original_Waves)
		min_curr[n]=V_min
		WaveStats/Q/R=(start_stim+stim_dur-0.1,start_stim+stim_dur)
	$StringFromList(n,Original_Waves)
		st_st[n]=V_avg
```

```
endfor



Display /W=(0,0,600,300)
for(n=0;n<itemsinlist(Original_Waves);n+=1)
	AppendtoGraph/L=L_traces/B=B_traces $StringFromList(n,Original_Waves)
	if(spike_num[n]==0 && spike_num[n-1]!=0)
		ModifyGraph lsize($StringFromList(n-1,Original_Waves))=2,rgb($StringFromList(n-1,Original_Waves))=(0,0,0)
		ModifyGraph lsize($StringFromList(n,Original_Waves))=2,rgb($StringFromList(n,Original_Waves))=(34952,34952,34952)
	endif
endfor
ModifyGraph axisEnab(L_traces)={0.55,1},axisEnab(B_traces)={0,0.45}

AppendtoGraph/L=L_min_stst/B=B_min_stst min_curr,st_st vs curr_steps
ModifyGraph axisEnab(L_min_stst)={0.55,1},axisEnab(B_min_stst)={0.55,1}
ModifyGraph mode(min_curr)=4,marker(min_curr)=19,rgb(min_curr)=(1,12815,52428),mode(st_st)=4,marker(st_st)=19,rgb(st_st)=(65535,0,26214)
ModifyGraph zero(L_min_stst)=4,zero(B_min_stst)=4,gridEnab(L_min_stst)={0.55,1},gridEnab(B_min_stst)={0.55,1}



AppendtoGraph/L=L_spike_num/B=B_spike_num spike_num vs curr_steps
ModifyGraph axisEnab(L_spike_num)={0,0.45},axisEnab(B_spike_num)={0.55,1}
ModifyGraph mode(spike_num)=4,marker(spike_num)=19,rgb(spike_num)=(0,0,0)
AppendtoGraph/R=R_spikes/B=B_spike_num IV_FSL vs curr_steps
ModifyGraph axisEnab(R_spikes)={0,0.45}
ModifyGraph mode(IV_FSL)=4,marker(IV_FSL)=19,rgb(IV_FSL)=(3,52428,1)

Variable idx_color=1
```

```
// Displays ISI of 200 pA above threshold until max

for(n=itemsinlist(ISI_waves);n>250/step_current;n-=1)

	AppendtoGraph/L=L_ISI/B=B_ISI $StringFromList(n-250/step_current,ISI_waves)

	ModifyGraph rgb($StringFromList(n-250/step_current,ISI_waves))=(0,65535/idx_color,65535)

	idx_color*=1.5


	ModifyGraph axisEnab(L_ISI)={0,0.45},axisEnab(B_ISI)={0,0.45}

endfor

ModifyGraph nticks=3,fSize=11,axThick=1.2,btLen=3

Legend/C/N=text0/J "\\Z08\\s(min_curr) min\r\\s(st_st) st_st\r\\s(spike_num) # of spikes\r\\s(IV_FSL) FSL"

Legend/C/N=text0/J/F=0/B=1


//ModifyGraph axisEnab(L_traces)={0.55,1},axisEnab(B_traces)={0,0.45}


ModifyGraph noLabel(L_traces)=2,noLabel(B_traces)=2,axThick(L_traces)=0,axThick(B_traces)=0

ModifyGraph lblPos(L_min_stst)=35,freePos(L_min_stst)=-270;DelayUpdate

Label L_min_stst "\\u#2min / st_st [mV]"

Label B_spike_num "Current [pA]";DelayUpdate

ModifyGraph freePos(B_spike_num)=0

ModifyGraph freePos(B_min_stst)=-127.059


Label L_spike_num "# of spikes";DelayUpdate

ModifyGraph freePos(L_spike_num)=-520


ModifyGraph lblPos(R_spikes)=-10,freePos(R_spikes)=-241.412;DelayUpdate

Label R_spikes "\\u#2FSL [ms]";DelayUpdate

SetAxis R_spikes 0,*

ModifyGraph lblPos(B_spike_num)=30,lblLatPos=0


for(n=0;n<itemsinlist(ISI_waves);n+=1)
```

```
	if(numpnts($StringFromList(n,ISI_waves))>1)
		ModifyGraph lblPos(B_ISI)=30;DelayUpdate
		ModifyGraph lblPos(L_ISI)=25,freePos(L_ISI)=0;DelayUpdate
		ModifyGraph freePos(B_ISI)=-1
		Label L_ISI "\\u#2ISI [ms]"
		Label B_ISI "# of spikes"
	endif
endfor

ModifyGraph zero(B_spike_num)=4
ModifyGraph zero(B_min_stst)=0
ModifyGraph nticks(B_min_stst)=5
ModifyGraph nticks(B_spike_num)=5



Edit curr_steps, min_curr, st_st, spike_num, IV_FSL
SetWindow kwTopWin//, sizeLimit={0,250,650,1200}
MoveWindow 625, 0, 1150, 300

end
```

```
Function Ramp()

Variable start_stim, start_current, step_current, percent_before, Level

start_stim=0.05
start_current=0
step_current=50
percent_before=25
```

```
Level=-0.02

String PopupSelection = "Yes?"       // Default value
String PopupOptions = "Yes;No"   // List of options

prompt start_stim,"Stimulus start at [s]: "
prompt start_current,"Start current [pA]: "
prompt step_current,"Step current [pA]: "
prompt percent_before,"Percent before dV max [%]: "
prompt Level,"Find AP Level [V]: "
prompt PopupSelection, "Single dV max?", popup, PopupOptions

Doprompt "Ramp",start_stim,start_current,step_current,percent_before, Level, PopupSelection
if ( V_Flag )
	return 0
endif

Variable/G start_stim_user=start_stim
Variable/G start_current_user=start_current
Variable/G step_current_user=step_current
Variable/G percent_before_user=percent_before
Variable/G Level_user=Level

String Original_Waves
Original_Waves=WaveList("*",";",("WIN:"))
Variable n

Make/O/N=1 W_FindLevels
Make/O/N=(itemsinlist(Original_Waves)) spike_time_Peak, spike_time_Peak_time, FSL,
Curr_Thres
```

```
Make/O/T/N=10 Ramp_desc={"Threshold [pA]","max AP [mV]","Vthres [mV]","AP_size_Erest [mV]","AP_size_Vthres [mV]","half_Erest [ms]","half_Vthres [ms]","dV_max","dV_min","Latency [ms]"}

Make/O/N=10 Ramp_prop

Wave W_FindLevels


for(n=0;n<itemsinlist(Original_Waves);n+=1)

	FindLevels/Q/R=(start_stim-0.001,start_stim+0.01) $StringFromList(n,Original_Waves), Level //Fixed search period

	if(V_flag==1)

		WaveStats/Q/R=(W_FindLevels[0],W_FindLevels[1]) $StringFromList(n,Original_Waves)

		spike_time_Peak[n]=V_max

		spike_time_Peak_time[n]=V_maxloc

		FSL[n]=V_maxloc-start_stim

		Curr_Thres[n]=start_current+n*step_current

	else

		spike_time_Peak[n]=NaN

		FSL[n]=NaN

		Curr_Thres[n]=NaN

	endif

endfor


// First suprathreshold

for(n=1;n<numpnts(Curr_Thres);n+=1)

	if (numtype(Curr_Thres[n-1])==2 && numtype(Curr_Thres[n])!=2)

		Ramp_prop[0]=Curr_Thres[n]

		Ramp_prop[1]=spike_time_Peak[n]*1000

		Wave new_wave=$StringFromList(n,Original_Waves)

		WaveStats/Q/R=(start_stim-0.005,start_stim) new_wave

		variable Erest=V_avg // Erest

		print("Erest: "+num2str(Erest*1000)+" mV")
```

```
Display/L=L_supra/B=B_up new_wave
ModifyGraph rgb=(0,0,0)
Differentiate new_wave/D=new_wave_DIF;DelayUpdate
AppendToGraph/L=L_diff/B=B_up new_wave_DIF
ModifyGraph rgb=(0,0,0)
ModifyGraph axisEnab(L_supra)={0.35,0.65},axisEnab(L_diff)={0.7,1}
SetAxis B_up start_stim,2*spike_time_Peak_time[n]-start_stim
ModifyGraph freePos(B_up)=-165

// Duplicate the wave for the phase plot later
Duplicate/O $StringFromList(n,Original_Waves), first_supra
Duplicate/O new_wave_DIF, first_supra_DIFF

// find max and min of Diff
Variable diff_max, diff_maxloc, diff_min, diff_max_75, diff_max_75_loc,max_AP,
test1
WaveStats/Q new_wave_DIF
Ramp_prop[7]=V_max
test1=V_maxloc
Ramp_prop[8]=V_min

if (CmpStr(PopupSelection, "No") == 0)
	Differentiate new_wave_DIF/D=new_wave_DIF_DIF;DelayUpdate
	WaveStats/Q new_wave_DIF_DIF
	Variable dif_dif_max=V_max
	FindLevels/Q/D=levels_Dif_Dif new_wave_DIF_DIF, dif_dif_max/2
	WaveStats/Q/R=(levels_Dif_Dif[0],levels_Dif_Dif[1]) new_wave_DIF_DIF
	diff_maxloc=V_maxloc
	//diff_max_75=V_max*0.75
	diff_max_75=V_max*((100-percent_before)/100)
```

```
			FindLevel/Q/R=(diff_maxloc-0.01,diff_maxloc) new_wave_DIF_DIF, 
diff_max_75
			diff_max_75_loc=V_LevelX
			Ramp_prop[2]=new_wave(diff_max_75_loc)*1000


		else
			diff_maxloc=V_maxloc
			diff_max_75=V_max*((100-percent_before)/100)


			FindLevel/Q/R=(diff_maxloc,diff_maxloc-0.01) new_wave_DIF, 
diff_max_75
			diff_max_75_loc=V_LevelX
			Ramp_prop[2]=new_wave(diff_max_75_loc)*1000
		endif


		WaveStats/Q/R=(diff_max_75_loc,diff_max_75_loc+0.001) new_wave
		max_AP=V_max
		Ramp_prop[9]=FSL[n]*1000
		Ramp_prop[3]=abs(V_max-Erest)*1000 // Find Max_AP from Erest
		Ramp_prop[4]=abs(V_max-Ramp_prop[2]/1000)*1000 // Find Max_AP from 
Vthres
		tag/A=RC $StringFromList(n,Original_Waves), max_AP, "max_AP" //tag the max
		//print(max_AP)
		Tag/C/N=text0/X=40.00/Y=3.00


		FindLevels/Q/R=(start_stim,start_stim+0.005)/D=levels_found 
$StringFromList(n,Original_Waves), max_AP-((max_AP-Ramp_prop[2]/1000)/2)
		Ramp_prop[6]=(levels_found[1]-levels_found[0])*1000 //halfwidth from Vthres
		tag/A=RC $StringFromList(n,Original_Waves), levels_found[1], "halfwidth 
[Vthres]"
		Tag/C/N=text1/X=45.00/Y=3


		Make/O/N=2 half_Vthres_helper
```

```
		half_Vthres_helper[0,1]=max_AP-((max_AP-Ramp_prop[2]/1000)/2)
		AppendToGraph/L=L_supra/B=B_up half_Vthres_helper vs levels_found
		ModifyGraph rgb(half_Vthres_helper)=(1,4,52428)

		FindLevels/Q/R=(start_stim,start_stim+0.005)/D=levels_found_2 $StringFromList(n,Original_Waves), max_AP-((max_AP-Erest)/2)
		Ramp_prop[5]=(levels_found_2[1]-levels_found_2[0])*1000 //halfwidth from Erest
		tag/A=RC $StringFromList(n,Original_Waves), levels_found_2[1], "halfwidth [Erest]"
		Tag/C/N=text2/X=40.00/Y=5
		Make/O/N=2 half_Erest_helper
		half_Erest_helper[0,1]=max_AP-((max_AP-Erest)/2)
		AppendToGraph/L=L_supra/B=B_up half_Erest_helper vs levels_found_2

		Make/O/N=2 help_x_diff, help_y_diff
		help_x_diff[0,1]=diff_max_75_loc
		help_y_diff[0]=Ramp_prop[8]
		help_y_diff[1]=Ramp_prop[7]
		AppendToGraph/L=new/B=B_up help_y_diff vs help_x_diff
		ModifyGraph axisEnab(new)={0.35, 1}
		ModifyGraph lstyle(help_y_diff)=3,rgb(help_y_diff)=(34952,34952,34952)
		ModifyGraph nticks=3,fSize=11,axThick=1.2,btLen=3

		ModifyGraph nticks(L_supra)=5,nticks(L_diff)=5
		ModifyGraph freePos(L_supra)=0,freePos(L_diff)=0
		ModifyGraph lsize(half_Vthres_helper)=2,lsize(half_Erest_helper)=2

		ModifyGraph width=300, height=500

		// Last suprathreshold curve
		Make/O/T/N=6 sub_desc={"Peak [mV]","time to Peak [ms]", "decay 50% [ms]", "decay 40% [ms]", "decay 30% [ms]", "halfwidth @-1mV"}
```

```
Make/O/N=6 sub_prop

Wave new_wave_2=$StringFromList(n-1,Original_Waves)
AppendToGraph/L=L_sub/B=B_sub new_wave_2
ModifyGraph axisEnab(L_sub)={0, 0.3}
ModifyGraph freePos(L_sub)=0
ModifyGraph rgb($StringFromList(n-1,Original_Waves))=(0,0,65535)
ModifyGraph freePos(B_sub)=0

WaveStats/Q/R=(start_stim-0.005,start_stim) new_wave_2
variable sub_min=V_min
variable Erest_sub=V_avg // Erest

WaveStats/Q/R=(start_stim,start_stim+0.05) new_wave_2
sub_prop[0]=V_max // 0 --> Peak
sub_prop[1]=V_maxloc // 1 --> time to Peak
    SetAxis B_sub start_stim-0.001,sub_prop[1]+0.02
    ModifyGraph fSize=11,axThick=1.2,btLen=3
FindLevel/Q/R=(sub_prop[1], sub_prop[1]+0.05) new_wave_2, sub_prop[0]-((sub_prop[0]-Erest_sub)*0.5)
sub_prop[2]=V_levelX // decay time to 50%
FindLevel/Q/R=(sub_prop[1], sub_prop[1]+0.05) new_wave_2, sub_prop[0]-((sub_prop[0]-Erest_sub)*0.6)
sub_prop[3]=V_levelX // decay time to 40%
FindLevel/Q/R=(sub_prop[1], sub_prop[1]+0.05) new_wave_2, sub_prop[0]-((sub_prop[0]-Erest_sub)*0.7)
sub_prop[4]=V_levelX // decay time to 30%

// halfwidth @1 mV below max
Variable max_1mV=(sub_prop[0]-0.001)
FindLevels/Q/D=Level_1mV new_wave_2, max_1mV
sub_prop[5]=(Level_1mV[1]-Level_1mV[0])
```

```
Make/O/N=2 half1mV_helper_x, half1mV_helper_y
half1mV_helper_y=max_1mV
half1mV_helper_x[0]=Level_1mV[0]
half1mV_helper_x[1]=Level_1mV[1]
AppendToGraph/L=L_sub/B=B_sub half1mV_helper_y vs half1mV_helper_x
ModifyGraph lstyle(half1mV_helper_y)=0,lsize(half1mV_helper_y)=2,rgb(half1mV_helper_y)=(1,39321,39321)
ModifyGraph noLabel(new)=2,axThick(new)=0
ModifyGraph lblPos(L_supra)=40,lblLatPos=0
ModifyGraph lblPos(L_diff)=40,lblLatPos(L_diff)=0
ModifyGraph lblPos(L_sub)=40,lblLatPos(L_sub)=0
ModifyGraph lblPos(B_sub)=30,lblLatPos=0
ModifyGraph lblPos(B_up)=30,lblLatPos=0

Make/O/N=4 sub_helper_x_50, sub_helper_x_40, sub_helper_x_30, sub_helper_y_50, sub_helper_y_40, sub_helper_y_30
sub_helper_x_50[0,1]=sub_prop[1]
sub_helper_x_50[2,3]=sub_prop[2]

sub_helper_x_40[0,1]=sub_prop[1]
sub_helper_x_40[2,3]=sub_prop[3]

sub_helper_x_30[0,1]=sub_prop[1]
sub_helper_x_30[2,3]=sub_prop[4]

sub_helper_y_50[1,2]=sub_prop[0]-((sub_prop[0]-Erest_sub)*0.5)
sub_helper_y_50[0]=sub_min
sub_helper_y_50[3]=sub_min

sub_helper_y_40[1,2]=sub_prop[0]-((sub_prop[0]-Erest_sub)*0.6)
sub_helper_y_40[0]=sub_min
sub_helper_y_40[3]=sub_min
```

```
		sub_helper_y_30[1,2]=sub_prop[0]-((sub_prop[0]-Erest_sub)*0.7)
		sub_helper_y_30[0]=sub_min
		sub_helper_y_30[3]=sub_min

		AppendToGraph/L=L_sub/B=B_sub sub_helper_y_50 vs sub_helper_x_50
		AppendToGraph/L=L_sub/B=B_sub sub_helper_y_40 vs sub_helper_x_40
		AppendToGraph/L=L_sub/B=B_sub sub_helper_y_30 vs sub_helper_x_30

		ModifyGraph
lstyle(sub_helper_y_50)=3,lstyle(sub_helper_y_40)=3,lstyle(sub_helper_y_30)=3
		ModifyGraph
rgb(sub_helper_y_50)=(39321,1,15729),rgb(sub_helper_y_40)=(0,17409,26214),rgb(sub_helper_
y_30)=(1,26214,0)

		Make/O/N=2 sub_helper_x_max
		Make/O/N=1 sub_helper_y, sub_helper_x_start

		sub_helper_x_max[0,1]=sub_prop[1]

		sub_helper_x_start[0]=start_stim
		sub_helper_y[0]=new_wave_2(start_stim)

		AppendToGraph/L=L_sub/B=B_sub sub_helper_y vs sub_helper_x_max
		AppendToGraph/L=L_sub/B=B_sub sub_helper_y vs sub_helper_x_start
		ModifyGraph rgb(sub_helper_y)=(0,0,0)
		ModifyGraph
mode(sub_helper_y#1)=3,marker(sub_helper_y#1)=8,opaque(sub_helper_y#1)=1,rgb(sub_helpe
r_y#1)=(65535,0,0)
		break

	// Phase Plot
```

```
		endif

	endfor

	StartWorkflow()
	//DoWindow/K kwTopWin

	Duplicate/O sub_prop, sub_prop_2
	sub_prop_2*=1000

	sub_prop_2[2,4]-=sub_prop_2[1]
	sub_prop_2[1]-=start_stim*1000

end
```

```
// Find Phase Plot

Function StartWorkflow()
	Wave first_supra, first_supra_DIFF
	Display first_supra_DIFF vs first_supra
	MoveWindow 750, 0, 1300, 250
	ShowInfo
```

```
  // Open panel
  DoWindow/F UserInputPanel
  if (V_Flag == 0)
    NewPanel/N=UserInputPanel/W=(100,100,360,200)

    Variable/G UserNumber = 0

    SetVariable svVal,pos={20,20},size={200,20},title="Enter number:",value= root:UserNumber
    Button btnOK,pos={20,60},title="OK",proc=UserInputPanelBtn
  endif

  // Return immediately! No waiting here.
End
```

```
Function UserInputPanelBtn(ctrlName) : ButtonControl
  String ctrlName

  DoWindow/K UserInputPanel   // Close panel

  // Now continue workflow
  ContinueWorkflow()

  return 0
End
```

```
Function ContinueWorkflow()

	DoWindow/K kwTopWin


	Wave Ramp_prop

	Wave first_supra

	Wave Ramp_desc, Ramp_prop, sub_desc, sub_prop_2

	NVAR start_stim_user, start_current_user, step_current_user, percent_before_user, Level_user, UserNumber


	Make/O/T/N=10 Ramp_desc_phase={"Threshold [pA]","max AP [mV]","Vthres [mV]","AP_size_Erest [mV]","AP_size_Vthres [mV]","half_Erest [ms]","half_Vthres [ms]","dV_max","dV_min","Latency [ms]"}

	Make/O/N=10 Ramp_prop_phase


	Ramp_prop_phase[7]=Ramp_prop[7]

	Ramp_prop_phase[8]=Ramp_prop[8]

	Ramp_prop_phase[9]=Ramp_prop[9]


	FindLevels/Q/R=(start_stim_user-0.001,start_stim_user+0.01)/D=levels_phase first_supra, Level_user //Fixed search period

	WaveStats/Q/R=(levels_phase[1]-levels_phase[0]) first_supra


		Variable spike_time_Peak_phase=V_max				// max mV

		Variable spike_time_Peak_time_phase=V_maxloc // time of max mV

		Variable FSL_phase=V_maxloc-start_stim_user


	Ramp_prop_phase[0]=Ramp_prop[0]

	Ramp_prop_phase[1]=V_max

	Ramp_prop_phase[2]=first_supra[UserNumber]
```

```
WaveStats/Q/R=(start_stim_user-0.005,start_stim_user) first_supra
variable Erest=V_avg // Erest

Ramp_prop_phase[3]=Ramp_prop_phase[1]-Erest // Vmax from Erest
Ramp_prop_phase[4]=Ramp_prop_phase[1]-Ramp_prop_phase[2] // Vmax from Vthres

// halfwidth values

    Display first_supra
    MoveWindow 750, 0, 1300, 250

    FindLevels/Q/R=(start_stim_user,start_stim_user+0.005)/D=levels_found_3 first_supra, Ramp_prop_phase[1]-((Ramp_prop_phase[1]-Ramp_prop_phase[2])/2)
    Ramp_prop_phase[6]=(levels_found_3[1]-levels_found_3[0])*1000 //halfwidth from Vthres
    tag/A=RC first_supra, levels_found_3[1], "halfwidth [Vthres]"
    Tag/C/N=text0/X=45.00/Y=3

    Make/O/N=2 half_Vthres_helper_phase
    half_Vthres_helper_phase[0,1]=Ramp_prop_phase[1]-((Ramp_prop_phase[1]-Ramp_prop_phase[2])/2)
    AppendToGraph half_Vthres_helper_phase vs levels_found_3

    FindLevels/Q/R=(start_stim_user,start_stim_user+0.005)/D=levels_found_4 first_supra, Ramp_prop_phase[1]-((Ramp_prop_phase[1]-Erest)/2)
    Ramp_prop_phase[5]=(levels_found_4[1]-levels_found_4[0])*1000 //halfwidth from Erest
    tag/A=RC first_supra, levels_found_4[1], "halfwidth [Vthres]"
    Tag/C/N=text1/X=30.00/Y=3

    Make/O/N=2 half_Vthres_helper_phase_2
    half_Vthres_helper_phase_2[0,1]=Ramp_prop_phase[1]-((Ramp_prop_phase[1]-Erest)/2)
    AppendToGraph half_Vthres_helper_phase_2 vs levels_found_4
```

```
	SetAxis bottom start_stim_user, 2*spike_time_Peak_time_phase-start_stim_user
	ModifyGraph rgb(first_supra)=(0,0,0)
	ModifyGraph lsize(half_Vthres_helper_phase)=2,lsize(half_Vthres_helper_phase_2)=2,rgb(half_Vthres_helper_phase_2)=(1,16019,65535)
DoWindow/K kwTopWin

AppendToGraph/L=L_supra/B=B_up half_Vthres_helper_phase vs levels_found_3
ModifyGraph lsize(half_Vthres_helper_phase)=2,rgb(half_Vthres_helper_phase)=(16385,28398,65535)

Ramp_prop_phase[1,4]*=1000
Edit Ramp_desc, Ramp_prop, Ramp_prop_phase
AppendToTable
AppendToTable sub_desc, sub_prop_2
SetWindow kwTopWin, sizeLimit={0,350,650,1200}
MoveWindow 500, 0, 1000, 300

end
```

```
Function Rheobase()

Variable start_stim, stim_dur, SampleRate, Level
String Folder
Folder=""

start_stim=0.05
stim_dur=0.35
SampleRate=2e-05
```

```
Level=-0.02

prompt start_stim,"Stimulus start at [s]: "
prompt stim_dur,"Stimulus Duration [s]: "
prompt SampleRate,"Sample Rate: "
prompt Level,"Find AP Level [V]: "
prompt Folder,"Name of Folder: "

Doprompt "Rheobase",start_stim, stim_dur, SampleRate, Level, Folder
if ( V_Flag )
	return 0
endif

if (CmpStr(Folder, "") != 0)
	NewDataFolder $Folder
endif

String Original_Waves
Original_Waves=WaveList("*",";",("WIN:"))

Variable n, m, k, idx_success, idx_failure
idx_success=1
idx_failure=1
for(n=0;n<itemsinlist(Original_Waves);n+=1)
	Wave new_wave=$StringFromList(n,Original_Waves)
	FindLevel/Q/R=(start_stim,start_stim+stim_dur) new_wave, Level
	if(V_flag==0)
		if(idx_success<10)
			Duplicate/O new_wave, $"success_0"+num2str(idx_success)
			if (CmpStr(Folder, "") != 0)
				MoveWave $"success_0"+num2str(idx_success), :$(Folder):
			endif
```

```
                idx_success+=1
            else
                Duplicate/O new_wave, $"success_"+num2str(idx_success)
                if (CmpStr(Folder, "") != 0)
                    MoveWave $"success_"+num2str(idx_success), :$(Folder):
                endif
                idx_success+=1
            endif
        else
            if(idx_failure<10)
                Duplicate/O new_wave, $"failure_0"+num2str(idx_failure)
                if (CmpStr(Folder, "") != 0)
                    MoveWave $"failure_0"+num2str(idx_failure), :$(Folder):
                endif
                idx_failure+=1
            else
                Duplicate/O new_wave, $"failure_"+num2str(idx_failure)
                if (CmpStr(Folder, "") != 0)
                    MoveWave $"failure_"+num2str(idx_failure), :$(Folder):
                endif
                idx_failure+=1
            endif
        endif

endfor

if (CmpStr(Folder, "") != 0)
    SetDataFolder :$(Folder):
endif

Make/O/T/N=8 Rheo_desc_phase={"Latency [ms]","jitter [ms]","Vthres [mV]","Success %","Spike number","Top 1mV [ms]","Time to Peak [ms]", "halfwidth"}
```

```
Make/O/N=8 Rheo_prop_phase

String Success_Waves
Success_Waves=WaveList("success_*",";",(""))
String Failure_Waves
Failure_Waves=WaveList("failure_*",";",(""))

Rheo_prop_phase[3]=itemsinlist(Success_Waves)/(itemsinlist(Success_Waves)+itemsinlist(Failure_Waves))
Rheo_prop_phase[4]=itemsinlist(Success_Waves)

Display
for(m=0;m<itemsinlist(Success_Waves);m+=1)
	Wave new_wave_succ=$StringFromList(m,Success_Waves)
	AppendToGraph/L=L_success new_wave_succ
endfor
for(k=0;k<itemsinlist(Failure_Waves);k+=1)
	Wave new_wave_fail=$StringFromList(k,Failure_Waves)
	AppendToGraph/L=L_failure new_wave_fail
endfor

ModifyGraph axisEnab(L_success)={0.55,1}
ModifyGraph axisEnab(L_failure)={0,0.45}
ModifyGraph fSize=11,axThick=1.2,btLen=3

SetWindow kwTopWin
MoveWindow 50, 0, 400, 500
ModifyGraph freePos(L_failure)=0
ModifyGraph freePos(L_success)=0
ModifyGraph lblPos(L_success)=50,lblPos(L_failure)=50

// 1. Subthreshold
```

```
for(k=0;k<itemsinlist(Failure_Waves);k+=1)
	Wave new_failed=$StringFromList(k,Failure_Waves)
	if(k==0)
		Duplicate/O new_failed, fail_avg
	else
		fail_avg+=new_failed
	endif
endfor
fail_avg/=itemsinlist(Failure_Waves)
AppendToGraph/L=L_failure fail_avg
ModifyGraph lsize(fail_avg)=2,rgb(fail_avg)=(0,0,0)

WaveStats/Q/R=(start_stim-0.01,start_stim) fail_avg
Variable Erest=V_avg // Erest

WaveStats/Q/R=(start_stim,start_stim+stim_dur) fail_avg
Variable fail_max_mV=V_max
Variable fail_maxloc_mV=V_maxloc
Rheo_prop_phase[6]=fail_maxloc_mV-start_stim // time to peak

FindLevels/Q/R=(start_stim,start_stim+stim_dur)/D=half_levels fail_avg, fail_max_mV-
(fail_max_mV-Erest)/2
WaveStats/Q half_levels
Rheo_prop_phase[7]=half_levels[V_npnts-1]-half_levels[0] // halfwidth

Make/O/N=2 fail_half_helper_x, fail_half_helper_y
fail_half_helper_x[0]=half_levels[0]
fail_half_helper_x[1]=half_levels[V_npnts-1]
fail_half_helper_y=fail_max_mV-(fail_max_mV-Erest)/2

AppendToGraph/L=L_failure fail_half_helper_y vs fail_half_helper_x
```

```
ModifyGraph rgb(fail_half_helper_y)=(1,16019,65535), lsize(fail_half_helper_y)=2

FindLevels/Q/R=(start_stim,start_stim+stim_dur)/D=half_levels_top5 fail_avg, fail_max_mV-(fail_max_mV-Erest)*0.05
WaveStats/Q half_levels_top5
Variable Top5mV=half_levels_top5[V_npnts-1]-half_levels_top5[0] // top5

//Make/O/N=2 fail_top5_helper_x, fail_top5_helper_y
//fail_top5_helper_x[0]=half_levels_top5[0]
//fail_top5_helper_x[1]=half_levels_top5[V_npnts-1]
//fail_top5_helper_y=fail_max_mV-(fail_max_mV-Erest)*0.05

//AppendToGraph/L=L_failure fail_top5_helper_y vs fail_top5_helper_x
//ModifyGraph rgb(fail_top5_helper_y)=(3,52428,1), lsize(fail_top5_helper_y)=1

FindLevels/Q/R=(start_stim,start_stim+stim_dur)/D=half_levels_top2 fail_avg, fail_max_mV-(fail_max_mV-Erest)*0.02
WaveStats/Q half_levels_top2
Variable Top2mV=half_levels_top2[V_npnts-1]-half_levels_top2[0] // top2

FindLevels/Q/R=(start_stim,start_stim+stim_dur)/D=half_levels_Top1mV fail_avg, fail_max_mV-0.001
WaveStats/Q half_levels_Top1mV
Rheo_prop_phase[5]=half_levels_Top1mV[V_npnts-1]-half_levels_Top1mV[0] // top1mV

Make/O/N=2 fail_top1mV_helper_x, fail_top1mV_helper_y
fail_top1mV_helper_x[0]=half_levels_Top1mV[0]
fail_top1mV_helper_x[1]=half_levels_Top1mV[V_npnts-1]
fail_top1mV_helper_y=fail_max_mV-0.001

AppendToGraph/L=L_failure fail_top1mV_helper_y vs fail_top1mV_helper_x
ModifyGraph rgb(fail_top1mV_helper_y)=(3,52428,1), lsize(fail_top1mV_helper_y)=2
```

```
CurveFit/Q/NTHR =0 dblexp_XOffset fail_avg(start_stim,fail_maxloc_mV) /D

Wave fit_fail_avg

ModifyGraph rgb(fit_fail_avg)=(1,52428,52428), lsize(fit_fail_avg)=2


Wave W_coef

Variable tau1=W_coef[2]

Variable tau2=W_coef[4]

Variable frac_tau1=W_coef[1]/(W_coef[1]+W_coef[3])


// 2. Suprathreshold


Make/O/N=(itemsinlist(Success_Waves)), latency, latency_max, Vthres_rheo_time_75,
Vthres_rheo_75, dif_max_75_spikes


for(m=0;m<itemsinlist(Success_Waves);m+=1)

	Wave success_new=$StringFromList(m,Success_Waves)

	FindLevels/Q/R=(start_stim,start_stim+stim_dur)/D=levels_found_success
$StringFromList(m,Success_Waves), Level

	WaveStats/Q/R=(levels_found_success[0],levels_found_success[1]) success_new

	Variable max_spike=V_maxloc

	latency[m]=V_maxloc-start_stim

	latency_max[m]=V_max


	Differentiate success_new/D=Diff_success;DelayUpdate

	WaveStats/Q Diff_success

	Variable dif_max=V_max

	FindLevel/Q/R=(start_stim,start_stim+stim_dur) Diff_success, dif_max*0.75


	Vthres_rheo_time_75[m]=V_LevelX

	Vthres_rheo_75=success_new(V_LevelX)


	Make/O/N=2 y_spikes
```

```
	y_spikes=success_new(Vthres_rheo_time_75[m])
	Duplicate/O y_spikes, $"y_spikes_"+num2str(m)
	KillWaves y_spikes

	Duplicate/O/R=(max_spike-0.002,max_spike+0.002) Diff_success, $"Diff_single_spikes_"+num2str(m)
	SetScale/P x 0,SampleRate,"s", $"Diff_single_spikes_"+num2str(m)
	KillWaves Diff_success
	Duplicate/O/R=(max_spike-0.002,max_spike+0.002) success_new, $"single_spikes_"+num2str(m)
	SetScale/P x 0,SampleRate,"s", $"single_spikes_"+num2str(m)
endfor

Duplicate/O latency, latency_2
latency_2+=start_stim
AppendToGraph/L=L_success latency_max vs latency_2
ModifyGraph mode(latency_max)=3,marker(latency_max)=8,opaque(latency_max)=1,rgb(latency_max)=(0,0,0), msize(latency_max)=2

WaveStats/Q latency
Rheo_prop_phase[0]=V_avg  // latency
Rheo_prop_phase[1]=V_sdev // jitter

String Success_single_spikes
Success_single_spikes=WaveList("single_spikes_*",";",(""))

Make/O/N=2 x_spikes={0,4/1000}

//Display
for(m=0;m<itemsinlist(Success_single_spikes);m+=1)
	AppendToGraph/L=L_suc_spikes/B=B_spikes $StringFromList(m,Success_single_spikes)
	AppendToGraph/L=L_suc_phase/B=B_spikes $"Diff_single_spikes_"+num2str(m)
```

```
	ModifyGraph rgb($"Diff_single_spikes_"+num2str(m))=(0,0,0)
	AppendToGraph/L=L_suc_spikes/B=B_spikes $"y_spikes_"+num2str(m) vs x_spikes
	ModifyGraph 
lstyle($"y_spikes_"+num2str(m))=3,rgb($"y_spikes_"+num2str(m))=(34952,34952,34952)
endfor

ModifyGraph axisEnab(L_suc_spikes)={0.7,0.83},axisEnab(L_suc_phase)={0.87,1},
axisEnab(B_spikes)={0.7,1}
ModifyGraph nticks=3,fSize=11,axThick=1.2,btLen=3
ModifyGraph 
lblPos(L_suc_spikes)=50,lblPos(L_suc_phase)=50,freePos(L_suc_spikes)=0,freePos(L_suc_phas
e)=0
ModifyGraph freePos(L_suc_spikes)=-430,freePos(L_suc_phase)=-430
ModifyGraph fSize(L_suc_phase)=5
ModifyGraph fSize(L_suc_spikes)=5
ModifyGraph noLabel(B_spikes)=2,axThick(B_spikes)=0

WaveStats/Q Vthres_rheo_75
Rheo_prop_phase[2]=V_avg

Edit Rheo_desc_phase, Rheo_prop_phase
SetWindow kwTopWin
MoveWindow 450, 0, 700, 220

SetDataFolder root:

end
```

```
Function Trains()

// Change the EPSP protocol --> delete specifically
Variable stim_art_del=80 //Change here
Variable Find_Level_point=-0.0



Variable SampleRate=1e-5
Variable start_stim, start_current, step_current, percent_before, Level

String PopupOptions_Protocol = "CC+Stim"      // Default value
String PopupSelection_Protocol = "VC;EPSP;CC+Stim"   // List of options
prompt PopupOptions_Protocol, "Protocol:", popup, PopupSelection_Protocol

String PopupOptions_Freq = "100"      // Default value
String PopupSelection_Freq = "100;200;300;400"   // List of options
prompt PopupOptions_Freq, "Frequency:", popup, PopupSelection_Freq

Doprompt "MNTB calyx+dendrites", PopupOptions_Protocol, PopupOptions_Freq
if ( V_Flag )
	return 0
endif

Variable freq_sel, Prot_sel

if (CmpStr(PopupOptions_Freq, "100") == 0)
	freq_sel=1000
elseif (CmpStr(PopupOptions_Freq, "200") == 0)
	freq_sel=500
```

```
elseif (CmpStr(PopupOptions_Freq, "300") == 0)
	freq_sel=330
elseif (CmpStr(PopupOptions_Freq, "400") == 0)
	freq_sel=250
endif




String Original_Waves
Original_Waves=WaveList("*",";",("WIN:"))
Variable n,m, k, l

if (CmpStr(PopupOptions_Protocol, "VC") == 0)
	for(n=0;n<itemsinlist(Original_Waves);n+=1)
		Duplicate/O $StringFromList(n,Original_Waves),
$"VC_"+num2str(n)+"_"+PopupOptions_Freq+"_Hz"
	endfor

	Display
	String VC_Trains
	VC_Trains=WaveList("VC_*"+PopupOptions_Freq+"_Hz",";",(""))

	for(k=0;k<itemsinlist(VC_Trains);k+=1)
		Wave new_wave=$StringFromList(k,VC_Trains)
		WaveStats/Q/R=[150,250] new_wave
		new_wave-=V_avg

		new_wave[0,140]=NaN
		for(m=0;m<50;m+=1)
			new_wave[5000+freq_sel*m,5000+stim_art_del+freq_sel*m]=NaN
```

```
			endfor

			if(k==0)
				Duplicate/O new_wave, avg_VC_trains
			else
				avg_VC_trains+=new_wave
			endif

		AppendToGraph $StringFromList(k, VC_Trains)
		ModifyGraph rgb($StringFromList(k, VC_Trains))=(65535-k*5000,0,0)

		endfor
		avg_VC_trains/=itemsinlist(VC_Trains)
		Duplicate/O avg_VC_trains, $"avg_VC_trains_"+PopupOptions_Freq+"_Hz"
		KillWaves avg_VC_trains

		AppendToGraph $"avg_VC_trains_"+PopupOptions_Freq+"_Hz"
		ModifyGraph rgb($"avg_VC_trains_"+PopupOptions_Freq+"_Hz")=(0,0,0)
		ModifyGraph lsize($"avg_VC_trains_"+PopupOptions_Freq+"_Hz")=2

	elseif (CmpStr(PopupOptions_Protocol, "EPSP") == 0)
		Make/O/N=(itemsinlist(Original_Waves)) Volt_avg
		for(n=0;n<itemsinlist(Original_Waves);n+=1)
```

```
			Duplicate/O $StringFromList(n,Original_Waves),
$"EPSP_"+num2str(n)+"_"+PopupOptions_Freq+"_Hz"
			WaveStats/Q/R=(0,0.02) $"EPSP_"+num2str(n)+"_"+PopupOptions_Freq+"_Hz"
			Volt_avg[n]=V_avg
		endfor

		WaveStats/Q Volt_avg
		Variable Volt_avg_all=V_avg

		Display
		String EPSP_Trains
		EPSP_Trains=WaveList("EPSP_*"+PopupOptions_Freq+"_Hz",";",(""))

		for(k=0;k<itemsinlist(EPSP_Trains);k+=1)
			Wave new_wave=$StringFromList(k,EPSP_Trains)
			new_wave+=(Volt_avg_all-Volt_avg[k])

			new_wave[0,140]=NaN
			for(m=0;m<50;m+=1)
				new_wave[5000+freq_sel*m,5000+stim_art_del+freq_sel*m]=NaN
				WaveStats/Q/R=(0.005, 0.025) new_wave
				Variable EPSP_avg_start=V_avg
				WaveStats/Q new_wave
				Variable EPSP_max_value=V_max
				FindLevel/Q/R=[5000+freq_sel*m,5000+freq_sel*(m+1)] new_wave,
EPSP_max_value-((EPSP_max_value-EPSP_avg_start)/2)
				if(V_flag==0)
					new_wave[5000+stim_art_del+freq_sel*m,
5000+stim_art_del+freq_sel*m+freq_sel]=NaN
				endif
			endfor

			//if(k==0)
```

```
		//	Duplicate/O new_wave, avg_EPSP_trains
		//else
		//	avg_EPSP_trains+=new_wave
		//endif

	AppendToGraph $StringFromList(k, EPSP_Trains)
	ModifyGraph rgb($StringFromList(k, EPSP_Trains))=(65535-k*5000,0,0)

	endfor
	//avg_EPSP_trains/=itemsinlist(EPSP_Trains)
	//Duplicate/O avg_EPSP_trains, $"avg_EPSP_trains_"+PopupOptions_Freq+"_Hz"

	//AppendToGraph $"avg_EPSP_trains_"+PopupOptions_Freq+"_Hz"
	//ModifyGraph rgb($"avg_EPSP_trains_"+PopupOptions_Freq+"_Hz")=(0,0,0)
	//ModifyGraph lsize($"avg_EPSP_trains_"+PopupOptions_Freq+"_Hz")=2



else
	Make/O/N=1 EPSG_lat_all, dend_lat_all
	Variable EPSG_lat_idx=0
	Variable dend_lat_idx=0
	Make/O/N=50 max_Diff_EPSG_avg, max_Diff_Dend_avg, Level_wave_EPSG_avg,
Level_wave_dend_avg, idx_EPSG, idx_dend
	for(n=0;n<itemsinlist(Original_Waves);n+=1)
	Make/O/N=50 max_Diff_EPSG, max_Diff_dend, Level_wave_EPSG, Level_wave_dend
	if(n==0)
		Display
	endif
		//Calyx
		Duplicate/O/R=(0.05,0.05+51*freq_sel*0.00001)
$StringFromList(n,Original_Waves), $"EPSG_"+PopupOptions_Freq+"_"+num2str(n)
```

```
			SetScale/P x 0,SampleRate,"s", $"EPSG_"+PopupOptions_Freq+"_"+num2str(n)
			//Differentiate
$"EPSG_"+PopupOptions_Freq+"_"+num2str(n)/D=$"Dif_EPSG_"+PopupOptions_Freq+"_"+num
2str(n);DelayUpdate


			for(m=0;m<50;m+=1)
				Duplicate/O/R=(0.05+m*freq_sel*0.00001,0.05+(m+1)*freq_sel*0.00001)
$StringFromList(n,Original_Waves),
$"s_EPSG_"+PopupOptions_Freq+"_"+num2str(n)+"_"+num2str(m)
				SetScale/P x 0,SampleRate,"s",
$"s_EPSG_"+PopupOptions_Freq+"_"+num2str(n)+"_"+num2str(m)
				AppendToGraph/L=Spikes
$"s_EPSG_"+PopupOptions_Freq+"_"+num2str(n)+"_"+num2str(m)
				//ModifyGraph
rgb($"s_EPSG_"+PopupOptions_Freq+"_"+num2str(n)+"_"+num2str(m))=(0,0,0)


				//Differentiate
$"s_EPSG_"+PopupOptions_Freq+"_"+num2str(n)+"_"+num2str(m)/D=$"Diff_s_EPSG_"+Popup
Options_Freq+"_"+num2str(n)+"_"+num2str(m);DelayUpdate
				//AppendToGraph/L=Diff
$"Diff_s_EPSG_"+PopupOptions_Freq+"_"+num2str(n)+"_"+num2str(m)



				FindLevel/Q/R=(0.0005,0.0025)
$"s_EPSG_"+PopupOptions_Freq+"_"+num2str(n)+"_"+num2str(m), Find_Level_point
				if(V_flag==0)
					Level_wave_EPSG[m]=V_LevelX
					EPSG_lat_all[EPSG_lat_idx]=V_LevelX
					InsertPoints EPSG_lat_idx+1,1, EPSG_lat_all
					EPSG_lat_idx+=1
				else
					if(n==0)
						Level_wave_EPSG[m]=0
					else
						Level_wave_EPSG[m]=NaN
					endif
```

```
				endif

				//WaveStats/Q/R=(0.001,0.0025)
$"Diff_s_EPSG_"+PopupOptions_Freq+"_"+num2str(n)+"_"+num2str(m)
				//max_Diff_EPSG[m]=V_maxloc



			endfor
			//max_Diff_EPSG_avg+=max_Diff_EPSG

			//Duplicate/O max_Diff_EPSG, $"max_Diff_EPSG_"+num2str(n)
			//KillWaves max_Diff_EPSG

			Variable idx_0

			for(idx_0=0;idx_0<50;idx_0+=1)
				if(numtype(Level_wave_EPSG[idx_0])!=2)
					Level_wave_EPSG_avg[idx_0]+=Level_wave_EPSG[idx_0]
					idx_EPSG[idx_0]+=1
				endif
			endfor



			Duplicate/O Level_wave_EPSG, $"Level_wave_EPSG_"+num2str(n)
			KillWaves Level_wave_EPSG



			//Calyx+dendrite stimulation

		Duplicate/O/R=(0.05+50*freq_sel*0.00001+0.5,0.05+50*freq_sel*0.00001+0.5+51*freq
_sel*0.00001) $StringFromList(n,Original_Waves),
$"DenStim_CC_"+PopupOptions_Freq+"_"+num2str(n)
```

```
SetScale/P x 0,SampleRate,"s", $"DenStim_CC_"+PopupOptions_Freq+"_"+num2str(n)
Wave new_dend=$"DenStim_CC_"+PopupOptions_Freq+"_"+num2str(n)
for(m=0;m<50;m+=1)
	new_dend[0+freq_sel*m,70+freq_sel*m]=NaN
endfor
//Differentiate $"DenStim_CC_"+PopupOptions_Freq+"_"+num2str(n)/D=$"Dif_DenStim_CC_"+PopupOptions_Freq+"_"+num2str(n);DelayUpdate


for(m=0;m<50;m+=1)


Duplicate/O/R=(0.05+50*freq_sel*0.00001+0.5+m*freq_sel*0.00001,0.05+50*freq_sel*0.00001+0.5+(m+1)*freq_sel*0.00001) $StringFromList(n,Original_Waves), $"s_Dend_"+PopupOptions_Freq+"_"+num2str(n)+"_"+num2str(m)
	SetScale/P x 0,SampleRate,"s", $"s_Dend_"+PopupOptions_Freq+"_"+num2str(n)+"_"+num2str(m)
	Wave zw=$"s_Dend_"+PopupOptions_Freq+"_"+num2str(n)+"_"+num2str(m)
	zw[0,50]=NaN
	AppendToGraph/L=Spikes $"s_Dend_"+PopupOptions_Freq+"_"+num2str(n)+"_"+num2str(m)
	ModifyGraph rgb($"s_Dend_"+PopupOptions_Freq+"_"+num2str(n)+"_"+num2str(m))=(0,0,0)


	// Find Levels
	FindLevel/Q/R=(0.0005,0.0025) $"s_Dend_"+PopupOptions_Freq+"_"+num2str(n)+"_"+num2str(m), Find_Level_point
	if(V_flag==0)
		Level_wave_dend[m]=V_LevelX
		dend_lat_all[dend_lat_idx]=V_LevelX
		InsertPoints dend_lat_idx+1,1, dend_lat_all
		dend_lat_idx+=1
	else
		if(n==0)
			Level_wave_dend[m]=0
```

```
			else
				Level_wave_dend[m]=NaN
			endif
		endif



	//	Differentiate
$"s_Dend_"+PopupOptions_Freq+"_"+num2str(n)+"_"+num2str(m)/D=$"Diff_s_Dend_"+PopupO
ptions_Freq+"_"+num2str(n)+"_"+num2str(m);DelayUpdate
		//AppendToGraph/L=Diff
$"Diff_s_Dend_"+PopupOptions_Freq+"_"+num2str(n)+"_"+num2str(m)
		////ModifyGraph
rgb($"Diff_s_Dend_"+PopupOptions_Freq+"_"+num2str(n)+"_"+num2str(m))=(0,0,0)

	//	WaveStats/Q/R=(0.001,0.002)
$"Diff_s_Dend_"+PopupOptions_Freq+"_"+num2str(n)+"_"+num2str(m)
	//	max_Diff_dend[m]=V_maxloc



	endfor

	//max_Diff_Dend_avg+=max_Diff_dend
	//Duplicate/O max_Diff_dend, $"max_Diff_dend_"+num2str(n)
	//KillWaves max_Diff_dend

	Variable idx_1

	for(idx_1=0;idx_1<50;idx_1+=1)
		if(numtype(Level_wave_dend[idx_1])!=2)
			Level_wave_dend_avg[idx_1]+=Level_wave_dend[idx_1]
			idx_dend[idx_1]+=1
		endif
	endfor
```

```
			Duplicate/O Level_wave_dend, $"Level_wave_dend_"+num2str(n)
			KillWaves Level_wave_dend



		//Display
		//AppendToGraph $"EPSG_"+PopupOptions_Freq+"_"+num2str(n)
		//AppendToGraph $"DenStim_CC_"+PopupOptions_Freq+"_"+num2str(n)
		//ModifyGraph rgb($"DenStim_CC_"+PopupOptions_Freq+"_"+num2str(n))=(0,0,0)

		endfor

	DeletePoints EPSG_lat_idx,1, EPSG_lat_all
	DeletePoints dend_lat_idx,1, dend_lat_all

	Level_wave_EPSG_avg/=idx_EPSG
	Level_wave_dend_avg/=idx_dend

	KillWaves idx_EPSG, idx_dend

	ModifyGraph fSize=11,axThick=1.2,btLen=3
	//ModifyGraph axisEnab(Spikes)={0.55,1},axisEnab(Diff)={0,0.45}
	SetAxis bottom *,0.003

	// Level_wave_EPSG_avg, Level_wave_dend_avg

	//MatrixOp/O Level_wave_EPSG_avg = mean(wave1, wave2, wave3)

	//max_Diff_EPSG_avg/=itemsinlist(Original_Waves)
	//max_Diff_Dend_avg/=itemsinlist(Original_Waves)

	Duplicate/O max_Diff_EPSG_avg, $"max_Diff_EPSG_avg_"+PopupOptions_Freq+"_Hz"
```

```
Duplicate/O max_Diff_Dend_avg, $"max_Diff_Dend_avg_"+PopupOptions_Freq+"_Hz"

KillWaves max_Diff_EPSG_avg, max_Diff_Dend_avg

//Level_wave_EPSG_avg/=itemsinlist(Original_Waves)
//Level_wave_dend_avg/=itemsinlist(Original_Waves)

Duplicate/O Level_wave_EPSG_avg, $"Level_wave_EPSG_avg_"+PopupOptions_Freq+"_Hz"
Duplicate/O Level_wave_dend_avg, $"Level_wave_dend_avg_"+PopupOptions_Freq+"_Hz"

KillWaves Level_wave_EPSG_avg, Level_wave_dend_avg

//Edit $"max_Diff_EPSG_avg_"+PopupOptions_Freq+"_Hz",
$"max_Diff_Dend_avg_"+PopupOptions_Freq+"_Hz"

//Display $"max_Diff_EPSG_avg_"+PopupOptions_Freq+"_Hz"
//AppendToGraph $"max_Diff_Dend_avg_"+PopupOptions_Freq+"_Hz"
//ModifyGraph rgb($"max_Diff_Dend_avg_"+PopupOptions_Freq+"_Hz")=(0,0,0)

//ModifyGraph fSize=11,axThick=1.2,btLen=3
//ModifyGraph mode=4,marker=19

Edit $"Level_wave_EPSG_avg_"+PopupOptions_Freq+"_Hz",
$"Level_wave_dend_avg_"+PopupOptions_Freq+"_Hz"

Display $"Level_wave_EPSG_avg_"+PopupOptions_Freq+"_Hz"
AppendToGraph $"Level_wave_dend_avg_"+PopupOptions_Freq+"_Hz"
ModifyGraph rgb($"Level_wave_dend_avg_"+PopupOptions_Freq+"_Hz")=(0,0,0)
```

```
ModifyGraph fSize=11,axThick=1.2,btLen=3
ModifyGraph mode=4,marker=19

Duplicate/O	EPSG_lat_all, $"EPSG_lat_all_"+PopupOptions_Freq+"_Hz"
Duplicate/O	dend_lat_all, $"dend_lat_all_"+PopupOptions_Freq+"_Hz"

WaveStats/Q $"EPSG_lat_all_"+PopupOptions_Freq+"_Hz"
Make/O/N=(V_npnts) EPSG_lat_all_x
EPSG_lat_all_x=1

WaveStats/Q $"dend_lat_all_"+PopupOptions_Freq+"_Hz"
Make/O/N=(V_npnts) dend_lat_all_x
dend_lat_all_x=2

Display;AppendViolinPlot $"EPSG_lat_all_"+PopupOptions_Freq+"_Hz" vs EPSG_lat_all_x
SetAxis bottom 0.5,2.5

AppendViolinPlot $"dend_lat_all_"+PopupOptions_Freq+"_Hz" vs dend_lat_all_x
ModifyViolinPlot
trace=$"EPSG_lat_all_"+PopupOptions_Freq+"_Hz",BoxWidth=100;DelayUpdate
ModifyViolinPlot trace=$"dend_lat_all_"+PopupOptions_Freq+"_Hz",BoxWidth=100

ModifyViolinPlot
trace=$"EPSG_lat_all_"+PopupOptions_Freq+"_Hz",LineThickness=0;DelayUpdate
ModifyViolinPlot trace=$"dend_lat_all_"+PopupOptions_Freq+"_Hz",LineThickness=0

ModifyGraph nticks=3,fSize=11,axThick=1.2,btLen=3

ModifyViolinPlot
trace=$"EPSG_lat_all_"+PopupOptions_Freq+"_Hz",ShowMean,MeanMarker=19,MeanMarkerCo
lor=(0,0,0);DelayUpdate
ModifyViolinPlot
trace=$"dend_lat_all_"+PopupOptions_Freq+"_Hz",ShowMean,MeanMarker=19,MeanMarkerCol
or=(0,0,0)
```

```
ModifyGraph nticks(left)=10

StatsTTest
$"EPSG_lat_all_"+PopupOptions_Freq+"_Hz",$"dend_lat_all_"+PopupOptions_Freq+"_Hz"

endif



end
```

```
Function AvgMultipleWavesNaN(waveList, outWaveName)
  // waveList: semicolon-separated wave names, e.g. "w1;w2;w3"
  // outWaveName: name of resulting wave
  String waveList
  String outWaveName

  Variable nWaves = ItemsInList(waveList)
  Variable i, p

  if (nWaves < 1)
    Abort "No waves provided!"
  endif

  // Use first wave to get length
  Wave w0 = $(StringFromList(0, waveList))
  Variable nPts = numpnts(w0)

  // Create output wave
  Make/O/N=(nPts) $outWaveName
```

```
    Wave wAvg = $outWaveName

    // Temporary variables
    Variable sum, count

    // Loop over points
    for (p = 0; p < nPts; p += 1)
        sum = 0
        count = 0

        // Loop over all waves
        for (i = 0; i < nWaves; i += 1)
            Wave w = $(StringFromList(i, waveList))
            if (numtype(w[p]) == 0)  // numeric
                sum += w[p]
                count += 1
            endif
        endfor

        // Assign average
        if (count > 0)
            wAvg[p] = sum / count
        else
            wAvg[p] = NaN
        endif
    endfor
End


// Bring all graphs in the average in the beginning

function helpme()
```

```
Wave EPSP_100_Hz_avg, EPSP_200_Hz_avg, EPSP_300_Hz_avg, EPSP_400_Hz_avg
Variable n
String Average_Waves
Average_Waves=WaveList("*",";",("WIN:"))

Make/O/N=(itemsinlist(Average_Waves)) avg_start_help
for(n=0;n<itemsinlist(Average_Waves);n+=1)
	Wave help_wave= $StringFromList(n,Average_Waves)
	SetScale/P x 0,1e-05,"s", help_wave
	WaveStats/Q/R=(0.01,0.02) help_wave
	avg_start_help[n]=V_avg
endfor

WaveStats/Q avg_start_help
Variable platz=V_avg

for(n=0;n<itemsinlist(Average_Waves);n+=1)
	Wave help_wave= $StringFromList(n,Average_Waves)
	help_wave-=(avg_start_help[n]-platz)
endfor

Display EPSP_100_Hz_avg
AppendToGraph/B=B_200 EPSP_200_Hz_avg
ModifyGraph freePos(B_200)=0
AppendToGraph/B=B_300 EPSP_300_Hz_avg
ModifyGraph freePos(B_300)=0
AppendToGraph/B=B_400 EPSP_400_Hz_avg
ModifyGraph freePos(B_400)=0

ModifyGraph nticks=3,fSize=11,axThick=1.2,btLen=3
ModifyGraph nticks(left)=5
```

Label left "\\u#2mV"

SetAxis B_200 0.0235,*

SetAxis B_300 0.032,*

SetAxis B_400 0.034,*

ModifyGraph noLabel(bottom)=2,noLabel(B_200)=2,noLabel(B_300)=2,noLabel(B_400)=2,axThick(bottom)=0,axThick(B_200)=0,axThick(B_300)=0,axThick(B_400)=0

ModifyGraph rgb(EPSP_200_Hz_avg)=(0,0,65535),rgb(EPSP_300_Hz_avg)=(0,65535,0),rgb(EPSP_400_Hz_avg)=(0,0,0)

•Legend/C/N=text0/J/F=0/A=MC "\\s(EPSP_100_Hz_avg) 100 Hz\r\\s(EPSP_200_Hz_avg) 200 Hz\r\\s(EPSP_300_Hz_avg) 300 Hz\r\\s(EPSP_400_Hz_avg) 400 Hz"

•Legend/C/N=text0/J/B=1

end

Function Trains_last_20()

// Change the EPSP protocol --> delete specifically

Variable stim_art_del=143 //Change here

Variable Find_Level_point=-0.02

Variable SampleRate=1e-5

Variable start_stim, start_current, step_current, percent_before, Level

String PopupOptions_Protocol = "CC+Stim"   // Default value

String PopupSelection_Protocol = "VC;EPSP;CC+Stim"   // List of options

```
prompt PopupOptions_Protocol, "Protocol:", popup, PopupSelection_Protocol

String PopupOptions_Freq = "100"       // Default value
String PopupSelection_Freq = "100;200;300;400"   // List of options
prompt PopupOptions_Freq, "Frequency:", popup, PopupSelection_Freq

Doprompt "MNTB calyx+dendrites", PopupOptions_Protocol, PopupOptions_Freq
if ( V_Flag )
	return 0
endif

Variable freq_sel, Prot_sel
Variable start_last_20

if (CmpStr(PopupOptions_Freq, "100") == 0)
	freq_sel=1000
	start_last_20=0.35
elseif (CmpStr(PopupOptions_Freq, "200") == 0)
	freq_sel=500
	start_last_20=0.2
elseif (CmpStr(PopupOptions_Freq, "300") == 0)
	freq_sel=330
	start_last_20=0.149
elseif (CmpStr(PopupOptions_Freq, "400") == 0)
	freq_sel=250
	start_last_20=0.1255
endif
```

```
String Original_Waves
Original_Waves=WaveList("*",";",("WIN:"))
Variable n,m, k, l

if (CmpStr(PopupOptions_Protocol, "VC") == 0)
	for(n=0;n<itemsinlist(Original_Waves);n+=1)
		Duplicate/O $StringFromList(n,Original_Waves),
$"VC_"+num2str(n)+"_"+PopupOptions_Freq+"_Hz"
	endfor

	Display
	String VC_Trains
	VC_Trains=WaveList("VC_*"+PopupOptions_Freq+"_Hz",";",(""))

	for(k=0;k<itemsinlist(VC_Trains);k+=1)
		Wave new_wave=$StringFromList(k,VC_Trains)
		WaveStats/Q/R=[150,250] new_wave
		new_wave-=V_avg

		new_wave[0,140]=NaN
		for(m=0;m<50;m+=1)
			new_wave[5000+freq_sel*m,5000+stim_art_del+freq_sel*m]=NaN
		endfor

		if(k==0)
			Duplicate/O new_wave, avg_VC_trains
		else
			avg_VC_trains+=new_wave
		endif

	AppendToGraph $StringFromList(k, VC_Trains)
	ModifyGraph rgb($StringFromList(k, VC_Trains))=(65535-k*5000,0,0)
```

```
	endfor
	avg_VC_trains/=itemsinlist(VC_Trains)
	Duplicate/O avg_VC_trains, $"avg_VC_trains_"+PopupOptions_Freq+"_Hz"
	KillWaves avg_VC_trains

	AppendToGraph $"avg_VC_trains_"+PopupOptions_Freq+"_Hz"
	ModifyGraph rgb($"avg_VC_trains_"+PopupOptions_Freq+"_Hz")=(0,0,0)
	ModifyGraph lsize($"avg_VC_trains_"+PopupOptions_Freq+"_Hz")=2

elseif (CmpStr(PopupOptions_Protocol, "EPSP") == 0)
	Make/O/N=(itemsinlist(Original_Waves)) Volt_avg
	for(n=0;n<itemsinlist(Original_Waves);n+=1)
		Duplicate/O $StringFromList(n,Original_Waves),
$"EPSP_"+num2str(n)+"_"+PopupOptions_Freq+"_Hz"
		WaveStats/Q/R=(0,0.02) $"EPSP_"+num2str(n)+"_"+PopupOptions_Freq+"_Hz"
		Volt_avg[n]=V_avg
	endfor

	WaveStats/Q Volt_avg
	Variable Volt_avg_all=V_avg

	Display
	String EPSP_Trains
```

```
	EPSP_Trains=WaveList("EPSP_*"+PopupOptions_Freq+"_Hz",";",(""))


	for(k=0;k<itemsinlist(EPSP_Trains);k+=1)
		Wave new_wave=$StringFromList(k,EPSP_Trains)
		new_wave+=(Volt_avg_all-Volt_avg[k])


		new_wave[0,140]=NaN
		for(m=0;m<20;m+=1)
			new_wave[5000+freq_sel*m,5000+stim_art_del+freq_sel*m]=NaN
			WaveStats/Q/R=(0.005, 0.025) new_wave
			Variable EPSP_avg_start=V_avg
			WaveStats/Q new_wave
			Variable EPSP_max_value=V_max
			FindLevel/Q/R=[35000+freq_sel*m,35000+freq_sel*(m+1)] new_wave,
EPSP_max_value-((EPSP_max_value-EPSP_avg_start)/2)
			if(V_flag==0)
				new_wave[35000+stim_art_del+freq_sel*m,
35000+stim_art_del+freq_sel*m+freq_sel]=NaN
			endif
		endfor


		//if(k==0)
		//	Duplicate/O new_wave, avg_EPSP_trains
		//else
		//	avg_EPSP_trains+=new_wave
		//endif


	AppendToGraph $StringFromList(k, EPSP_Trains)
	ModifyGraph rgb($StringFromList(k, EPSP_Trains))=(65535-k*5000,0,0)


	endfor
	//avg_EPSP_trains/=itemsinlist(EPSP_Trains)
```

```
//Duplicate/O avg_EPSP_trains, $"avg_EPSP_trains_"+PopupOptions_Freq+"_Hz"


//AppendToGraph $"avg_EPSP_trains_"+PopupOptions_Freq+"_Hz"

//ModifyGraph rgb($"avg_EPSP_trains_"+PopupOptions_Freq+"_Hz")=(0,0,0)

//ModifyGraph lsize($"avg_EPSP_trains_"+PopupOptions_Freq+"_Hz")=2





else

Make/O/N=1 EPSG_lat_all, dend_lat_all

Variable EPSG_lat_idx=0

Variable dend_lat_idx=0

Make/O/N=20 max_Diff_EPSG_avg, max_Diff_Dend_avg, Level_wave_EPSG_avg,
Level_wave_dend_avg, idx_EPSG, idx_dend

for(n=0;n<itemsinlist(Original_Waves);n+=1)

Make/O/N=20 max_Diff_EPSG, max_Diff_dend, Level_wave_EPSG, Level_wave_dend

if(n==0)

Display

endif

//Calyx

Duplicate/O/R=(start_last_20,start_last_20+20*freq_sel*0.00001)
$StringFromList(n,Original_Waves), $"EPSG_"+PopupOptions_Freq+"_"+num2str(n)

SetScale/P x 0,SampleRate,"s", $"EPSG_"+PopupOptions_Freq+"_"+num2str(n)

//Differentiate
$"EPSG_"+PopupOptions_Freq+"_"+num2str(n)/D=$"Dif_EPSG_"+PopupOptions_Freq+"_"+num
2str(n);DelayUpdate


for(m=0;m<20;m+=1)


Duplicate/O/R=(start_last_20+m*freq_sel*0.00001,start_last_20+(m+1)*freq_sel*0.000
01) $StringFromList(n,Original_Waves),
$"s_EPSG_"+PopupOptions_Freq+"_"+num2str(n)+"_"+num2str(m)

SetScale/P x 0,SampleRate,"s",
$"s_EPSG_"+PopupOptions_Freq+"_"+num2str(n)+"_"+num2str(m)
```

```
			AppendToGraph/L=Spikes
$"s_EPSG_"+PopupOptions_Freq+"_"+num2str(n)+"_"+num2str(m)
			//ModifyGraph
rgb($"s_EPSG_"+PopupOptions_Freq+"_"+num2str(n)+"_"+num2str(m))=(0,0,0)


			//Differentiate
$"s_EPSG_"+PopupOptions_Freq+"_"+num2str(n)+"_"+num2str(m)/D=$"Diff_s_EPSG_"+Popup
Options_Freq+"_"+num2str(n)+"_"+num2str(m);DelayUpdate
			//AppendToGraph/L=Diff
$"Diff_s_EPSG_"+PopupOptions_Freq+"_"+num2str(n)+"_"+num2str(m)



			FindLevel/Q/R=(0.0005,0.0025)
$"s_EPSG_"+PopupOptions_Freq+"_"+num2str(n)+"_"+num2str(m), Find_Level_point
			if(V_flag==0)
				Level_wave_EPSG[m]=V_LevelX
				EPSG_lat_all[EPSG_lat_idx]=V_LevelX
				InsertPoints EPSG_lat_idx+1,1, EPSG_lat_all
				EPSG_lat_idx+=1
			else
				if(n==0)
					Level_wave_EPSG[m]=0
				else
					Level_wave_EPSG[m]=NaN
				endif
			endif


			//WaveStats/Q/R=(0.001,0.0025)
$"Diff_s_EPSG_"+PopupOptions_Freq+"_"+num2str(n)+"_"+num2str(m)
			//max_Diff_EPSG[m]=V_maxloc



		endfor
		//max_Diff_EPSG_avg+=max_Diff_EPSG
```

```
//Duplicate/O max_Diff_EPSG, $"max_Diff_EPSG_"+num2str(n)
//KillWaves max_Diff_EPSG

Variable idx_0

for(idx_0=0;idx_0<20;idx_0+=1)
	if(numtype(Level_wave_EPSG[idx_0])!=2)
		Level_wave_EPSG_avg[idx_0]+=Level_wave_EPSG[idx_0]
		idx_EPSG[idx_0]+=1
	endif
endfor


Duplicate/O Level_wave_EPSG, $"Level_wave_EPSG_"+num2str(n)
KillWaves Level_wave_EPSG


//Calyx+dendrite stimulation

Duplicate/O/R=(start_last_20+20*freq_sel*0.00001+0.5,start_last_20+20*freq_sel*0.00
001+0.5+20*freq_sel*0.00001) $StringFromList(n,Original_Waves),
$"DenStim_CC_"+PopupOptions_Freq+"_"+num2str(n)
SetScale/P x 0,SampleRate,"s",
$"DenStim_CC_"+PopupOptions_Freq+"_"+num2str(n)
Wave new_dend=$"DenStim_CC_"+PopupOptions_Freq+"_"+num2str(n)
for(m=0;m<20;m+=1)
	new_dend[0+freq_sel*m,70+freq_sel*m]=NaN
endfor
//Differentiate
$"DenStim_CC_"+PopupOptions_Freq+"_"+num2str(n)/D=$"Dif_DenStim_CC_"+PopupOptions
_Freq+"_"+num2str(n);DelayUpdate

for(m=0;m<20;m+=1)
```

```
	Duplicate/O/R=(start_last_20+20*freq_sel*0.00001+0.5+m*freq_sel*0.00001+30*freq_s
el*0.00001 ,
start_last_20+20*freq_sel*0.00001+30*freq_sel*0.00001+0.5+(m+1)*freq_sel*0.00001)
$StringFromList(n,Original_Waves),
$"s_Dend_"+PopupOptions_Freq+"_"+num2str(n)+"_"+num2str(m)

				SetScale/P x 0,SampleRate,"s",
$"s_Dend_"+PopupOptions_Freq+"_"+num2str(n)+"_"+num2str(m)

				if (CmpStr(PopupOptions_Freq, "400") == 0)

					Wave
Hz_400_NaN=$"s_Dend_"+PopupOptions_Freq+"_"+num2str(n)+"_"+num2str(m)

					Hz_400_NaN[200,inf]=NaN

				endif

				Wave
zw=$"s_Dend_"+PopupOptions_Freq+"_"+num2str(n)+"_"+num2str(m)

				zw[0,50]=NaN

				AppendToGraph/L=Spikes
$"s_Dend_"+PopupOptions_Freq+"_"+num2str(n)+"_"+num2str(m)

				ModifyGraph
rgb($"s_Dend_"+PopupOptions_Freq+"_"+num2str(n)+"_"+num2str(m))=(0,0,0)


				// Find Levels

				FindLevel/Q/R=(0.0005,0.002)
$"s_Dend_"+PopupOptions_Freq+"_"+num2str(n)+"_"+num2str(m), Find_Level_point

				if(V_flag==0)

					Level_wave_dend[m]=V_LevelX

					dend_lat_all[dend_lat_idx]=V_LevelX

					InsertPoints dend_lat_idx+1,1, dend_lat_all

					dend_lat_idx+=1

				else

					if(n==0)

						Level_wave_dend[m]=0

					else

						Level_wave_dend[m]=NaN

					endif

				endif
```

```
			//	Differentiate
$"s_Dend_"+PopupOptions_Freq+"_"+num2str(n)+"_"+num2str(m)/D=$"Diff_s_Dend_"+PopupOptions_Freq+"_"+num2str(n)+"_"+num2str(m);DelayUpdate
				//AppendToGraph/L=Diff
$"Diff_s_Dend_"+PopupOptions_Freq+"_"+num2str(n)+"_"+num2str(m)
				////ModifyGraph
rgb($"Diff_s_Dend_"+PopupOptions_Freq+"_"+num2str(n)+"_"+num2str(m))=(0,0,0)


			//	WaveStats/Q/R=(0.001,0.002)
$"Diff_s_Dend_"+PopupOptions_Freq+"_"+num2str(n)+"_"+num2str(m)
			//	max_Diff_dend[m]=V_maxloc



			endfor


			//max_Diff_Dend_avg+=max_Diff_dend
			//Duplicate/O max_Diff_dend, $"max_Diff_dend_"+num2str(n)
			//KillWaves max_Diff_dend


			Variable idx_1


			for(idx_1=0;idx_1<20;idx_1+=1)
				if(numtype(Level_wave_dend[idx_1])!=2)
					Level_wave_dend_avg[idx_1]+=Level_wave_dend[idx_1]
					idx_dend[idx_1]+=1
				endif
			endfor


			Duplicate/O Level_wave_dend, $"Level_wave_dend_"+num2str(n)
			KillWaves Level_wave_dend
```

```
	//Display
	//AppendToGraph $"EPSG_"+PopupOptions_Freq+"_"+num2str(n)
	//AppendToGraph $"DenStim_CC_"+PopupOptions_Freq+"_"+num2str(n)
	//ModifyGraph rgb($"DenStim_CC_"+PopupOptions_Freq+"_"+num2str(n))=(0,0,0)

	endfor

DeletePoints EPSG_lat_idx,1, EPSG_lat_all
DeletePoints dend_lat_idx,1, dend_lat_all

Level_wave_EPSG_avg/=idx_EPSG
Level_wave_dend_avg/=idx_dend

KillWaves idx_EPSG, idx_dend

ModifyGraph fSize=11,axThick=1.2,btLen=3
//ModifyGraph axisEnab(Spikes)={0.55,1},axisEnab(Diff)={0,0.45}
SetAxis bottom *,0.003

// Level_wave_EPSG_avg, Level_wave_dend_avg

//MatrixOp/O Level_wave_EPSG_avg = mean(wave1, wave2, wave3)

//max_Diff_EPSG_avg/=itemsinlist(Original_Waves)
//max_Diff_Dend_avg/=itemsinlist(Original_Waves)

Duplicate/O max_Diff_EPSG_avg, $"max_Diff_EPSG_avg_"+PopupOptions_Freq+"_Hz"
Duplicate/O max_Diff_Dend_avg, $"max_Diff_Dend_avg_"+PopupOptions_Freq+"_Hz"

KillWaves max_Diff_EPSG_avg, max_Diff_Dend_avg
```

```
//Level_wave_EPSG_avg/=itemsinlist(Original_Waves)

//Level_wave_dend_avg/=itemsinlist(Original_Waves)


Duplicate/O Level_wave_EPSG_avg, $"Level_wave_EPSG_avg_"+PopupOptions_Freq+"_Hz"

Duplicate/O Level_wave_dend_avg, $"Level_wave_dend_avg_"+PopupOptions_Freq+"_Hz"


KillWaves Level_wave_EPSG_avg, Level_wave_dend_avg


//Edit $"max_Diff_EPSG_avg_"+PopupOptions_Freq+"_Hz",
$"max_Diff_Dend_avg_"+PopupOptions_Freq+"_Hz"


//Display $"max_Diff_EPSG_avg_"+PopupOptions_Freq+"_Hz"

//AppendToGraph $"max_Diff_Dend_avg_"+PopupOptions_Freq+"_Hz"

//ModifyGraph rgb($"max_Diff_Dend_avg_"+PopupOptions_Freq+"_Hz")=(0,0,0)



//ModifyGraph fSize=11,axThick=1.2,btLen=3

//ModifyGraph mode=4,marker=19



Edit $"Level_wave_EPSG_avg_"+PopupOptions_Freq+"_Hz",
$"Level_wave_dend_avg_"+PopupOptions_Freq+"_Hz"


Display $"Level_wave_EPSG_avg_"+PopupOptions_Freq+"_Hz"

AppendToGraph $"Level_wave_dend_avg_"+PopupOptions_Freq+"_Hz"

ModifyGraph rgb($"Level_wave_dend_avg_"+PopupOptions_Freq+"_Hz")=(0,0,0)



ModifyGraph fSize=11,axThick=1.2,btLen=3

ModifyGraph mode=4,marker=19


Duplicate/O     EPSG_lat_all, $"EPSG_lat_all_"+PopupOptions_Freq+"_Hz"
```

```
Duplicate/O     dend_lat_all, $"dend_lat_all_"+PopupOptions_Freq+"_Hz"

WaveStats/Q $"EPSG_lat_all_"+PopupOptions_Freq+"_Hz"
Make/O/N=(V_npnts) EPSG_lat_all_x
EPSG_lat_all_x=1

WaveStats/Q $"dend_lat_all_"+PopupOptions_Freq+"_Hz"
Make/O/N=(V_npnts) dend_lat_all_x
dend_lat_all_x=2

Display;AppendViolinPlot $"EPSG_lat_all_"+PopupOptions_Freq+"_Hz" vs EPSG_lat_all_x
SetAxis bottom 0.5,2.5

AppendViolinPlot $"dend_lat_all_"+PopupOptions_Freq+"_Hz" vs dend_lat_all_x
ModifyViolinPlot
trace=$"EPSG_lat_all_"+PopupOptions_Freq+"_Hz",BoxWidth=100;DelayUpdate
ModifyViolinPlot trace=$"dend_lat_all_"+PopupOptions_Freq+"_Hz",BoxWidth=100

ModifyViolinPlot
trace=$"EPSG_lat_all_"+PopupOptions_Freq+"_Hz",LineThickness=0;DelayUpdate
ModifyViolinPlot trace=$"dend_lat_all_"+PopupOptions_Freq+"_Hz",LineThickness=0

ModifyGraph nticks=3,fSize=11,axThick=1.2,btLen=3

ModifyViolinPlot
trace=$"EPSG_lat_all_"+PopupOptions_Freq+"_Hz",ShowMean,MeanMarker=19,MeanMarkerCo
lor=(0,0,0);DelayUpdate
ModifyViolinPlot
trace=$"dend_lat_all_"+PopupOptions_Freq+"_Hz",ShowMean,MeanMarker=19,MeanMarkerCol
or=(0,0,0)

ModifyGraph nticks(left)=10
```

```
StatsTTest
$"EPSG_lat_all_"+PopupOptions_Freq+"_Hz",$"dend_lat_all_"+PopupOptions_Freq+"_Hz"

endif

end
```

```
function maxVm()

Variable start_stim_100=141
Variable start_stim_200=141
Variable start_stim_300=141
Variable start_stim_400=141

Variable n
Wave EPSP_100_Hz_avg, EPSP_200_Hz_avg, EPSP_300_Hz_avg, EPSP_400_Hz_avg

WaveStats/Q/R=[500, 4500] EPSP_100_Hz_avg
	Variable start_avg_100=V_avg
WaveStats/Q/R=[500, 4500] EPSP_200_Hz_avg
	Variable start_avg_200=V_avg
WaveStats/Q/R=[500, 4500] EPSP_300_Hz_avg
	Variable start_avg_300=V_avg
WaveStats/Q/R=[500, 4500] EPSP_400_Hz_avg
	Variable start_avg_400=V_avg
```

```
Make/O/N=50 S_100_max, S_200_max, S_300_max, S_400_max
Make/O/N=50 S_100_avg, S_200_avg, S_300_avg, S_400_avg

for(n=0;n<50;n+=1)
	WaveStats/Q/R=[5000+start_stim_100+n*1000 , 5999+n*1000] EPSP_100_Hz_avg
		S_100_max[n]=V_max-start_avg_100
		S_100_avg[n]=V_avg-start_avg_100
	WaveStats/Q/R=[5000+start_stim_100+n*500 , 5499+n*500] EPSP_200_Hz_avg
		S_200_max[n]=V_max-start_avg_200
		S_200_avg[n]=V_avg-start_avg_200
	WaveStats/Q/R=[5000+start_stim_100+n*330 , 5329+n*330] EPSP_300_Hz_avg
		S_300_max[n]=V_max-start_avg_300
		S_300_avg[n]=V_avg-start_avg_300
	WaveStats/Q/R=[5000+start_stim_100+n*250 , 5249+n*250] EPSP_400_Hz_avg
		S_400_max[n]=V_max-start_avg_400
		S_400_avg[n]=V_avg-start_avg_400

endfor

Display S_100_max;AppendToGraph S_200_max, S_300_max, S_400_max

ModifyGraph rgb(S_200_max)=(1,16019,65535)
ModifyGraph rgb(S_300_max)=(3,52428,1)
ModifyGraph rgb(S_400_max)=(0,0,0)
ModifyGraph mode=4,marker=19

Display S_100_avg;AppendToGraph S_200_avg, S_300_avg, S_400_avg
ModifyGraph rgb(S_200_avg)=(1,16019,65535)
ModifyGraph rgb(S_300_avg)=(3,52428,1)
ModifyGraph rgb(S_400_avg)=(0,0,0)
ModifyGraph mode=4,marker=19
```

end
