# Supplementary material for: Non-calyceal inputs gate the timing of calyx of Held evoked MNTB output
Source: Commun Biol. 2026 May 22;9:697. doi: 10.1038/s42003-026-10321-w (PMC13197447; doi:10.1038/s42003-026-10321-w)
Supplement: Supplementary file 5 — Supplementary code [file 42003_2026_10321_MOESM5_ESM.pdf]

```
#pragma TextEncoding = "UTF-8"
#pragma rtGlobals=3				// Use modern global access method and strict wave access
#pragma DefaultTab={3,20,4}		// Set default tab width in Igor Pro 9 and later

Menu "Macros"
  SubMenu "Fit the first EPSC"
    "10Hz", /Q, GettheEPCSs_10Hz()
    "50Hz", /Q, GettheEPCSs_50Hz()
    "100Hz", /Q, GettheEPCSs_100Hz()
    "200Hz", /Q, GettheEPCSs_200Hz()
    "300Hz", /Q, GettheEPCSs_300Hz()
    "400Hz", /Q, GettheEPCSs_400Hz()

  End
End
/////////------------------------------------------------------------------------------------///////////
Function GettheEPCSs_10Hz()
Rep1_10()
Rep2_10()
Rep3_10()
end

function Rep1_10()
Wave platz_NaN_10_rep_0,platz_NaN_10_rep_1,platz_NaN_10_rep_rep_2,W_Coef, FitsEPSC_platz_NaN_10Hz_1_rep_0,fit_EPSC1st_platz_NaN_10Hz_1_rep_0, fitmono_EPSC1st_platz_NaN_10Hz_1_rep_0
Variable Beginning, Ending

Make/O/N=5 W_Coef

Beginning=1839
Ending=5753
```

```
Duplicate/R= [Beginning,Ending] platz_NaN_10_rep_0,EPSC1st_platz_NaN_10Hz_1_rep_0

Display EPSC1st_platz_NaN_10Hz_1_rep_0

make/o/n=7 FitsEPSC_platz_NaN_10Hz_1_rep_0

Wavestats EPSC1st_platz_NaN_10Hz_1_rep_0

CurveFit exp_XOffset EPSC1st_platz_NaN_10Hz_1_rep_0 [V_minRowLoc,V_minRowLoc+200] /D

FitsEPSC_platz_NaN_10Hz_1_rep_0[0]=W_Coef[1]

FitsEPSC_platz_NaN_10Hz_1_rep_0[1]=W_Coef[2]

wave fit_EPSC1st_platz_NaN_10Hz_1_rep_0, fitmono_EPSC1st_platz_NaN_10Hz_1_rep_0

Duplicate/O fit_EPSC1st_platz_NaN_10Hz_1_rep_0,
'fitmono_EPSC1st_platz_NaN_10Hz_1_rep_0'


CurveFit dblexp_XOffset EPSC1st_platz_NaN_10Hz_1_rep_0 [V_minRowLoc,V_minRowLoc+200]
/D

FitsEPSC_platz_NaN_10Hz_1_rep_0[2]=W_Coef[1]

FitsEPSC_platz_NaN_10Hz_1_rep_0[3]=W_Coef[2]

FitsEPSC_platz_NaN_10Hz_1_rep_0[4]=W_Coef[3]

FitsEPSC_platz_NaN_10Hz_1_rep_0[5]=W_Coef[4]

FitsEPSC_platz_NaN_10Hz_1_rep_0[6]=(W_coef[2]*(-1*W_coef[1])+W_coef[4]*(-
1*W_coef[3]))/((-1*W_coef[1])+(-1*W_coef[3]))


end


function Rep2_10()

Wave platz_NaN_10_rep_0,platz_NaN_10_rep_1,Pplatz_NaN_10_rep_2,W_Coef,
FitsEPSC_platz_NaN_10Hz_1_rep_0,fit_EPSC1st_platz_NaN_10Hz_1_rep_1,
fitmono_EPSC1st_platz_NaN_10Hz_1_rep_1

Variable Beginning, Ending


Beginning=1839

Ending=5753

Duplicate/R= [Beginning,Ending] platz_NaN_10_rep_1,EPSC1st_platz_NaN_10Hz_1_rep_1

Display EPSC1st_platz_NaN_10Hz_1_rep_1

make/o/n=7 FitsEPSC_platz_NaN_10Hz_1_rep_1
```

```
Wavestats EPSC1st_platz_NaN_10Hz_1_rep_1

CurveFit exp_XOffset EPSC1st_platz_NaN_10Hz_1_rep_1 [V_minRowLoc,V_minRowLoc+200] /D

FitsEPSC_platz_NaN_10Hz_1_rep_1[0]=W_Coef[1]

FitsEPSC_platz_NaN_10Hz_1_rep_1[1]=W_Coef[2]

wave fit_EPSC1st_platz_NaN_10Hz_1_rep_1, fitmono_EPSC1st_platz_NaN_10Hz_1_rep_1

Duplicate/O fit_EPSC1st_platz_NaN_10Hz_1_rep_1,
'fitmono_EPSC1st_platz_NaN_10Hz_1_rep_1'


CurveFit dblexp_XOffset EPSC1st_platz_NaN_10Hz_1_rep_1 [V_minRowLoc,V_minRowLoc+200]
/D

FitsEPSC_platz_NaN_10Hz_1_rep_1[2]=W_Coef[1]

FitsEPSC_platz_NaN_10Hz_1_rep_1[3]=W_Coef[2]

FitsEPSC_platz_NaN_10Hz_1_rep_1[4]=W_Coef[3]

FitsEPSC_platz_NaN_10Hz_1_rep_1[5]=W_Coef[4]

FitsEPSC_platz_NaN_10Hz_1_rep_1[6]=(W_coef[2]*(-1*W_coef[1])+W_coef[4]*(-
1*W_coef[3]))/((-1*W_coef[1])+(-1*W_coef[3]))


end


function Rep3_10()

Wave platz_NaN_10_rep_0,platz_NaN_10_rep_1,platz_NaN_10_rep_2,W_Coef,
FitsEPSC_platz_NaN_10Hz_1_rep_2,fit_EPSC1st_platz_NaN_10Hz_1_rep_2,
fitmono_EPSC1st_platz_NaN_10Hz_1_rep_2, FitsEPSC_platz_NaN_10Hz_1_rep_0,
FitsEPSC_platz_NaN_10Hz_1_rep_1

Variable Beginning, Ending


Beginning=1839

Ending=5753

Duplicate/R= [Beginning,Ending] platz_NaN_10_rep_2,EPSC1st_platz_NaN_10Hz_1_rep_2

Display EPSC1st_platz_NaN_10Hz_1_rep_2

make/o/n=7 FitsEPSC_platz_NaN_10Hz_1_rep_2

Wavestats EPSC1st_platz_NaN_10Hz_1_rep_2

CurveFit exp_XOffset EPSC1st_platz_NaN_10Hz_1_rep_2 [V_minRowLoc,V_minRowLoc+200] /D

FitsEPSC_platz_NaN_10Hz_1_rep_2[0]=W_Coef[1]
```

```
FitsEPSC_platz_NaN_10Hz_1_rep_2[1]=W_Coef[2]

wave fit_EPSC1st_platz_NaN_10Hz_1_rep_2, fitmono_EPSC1st_platz_NaN_10Hz_1_rep_2

Duplicate/O fit_EPSC1st_platz_NaN_10Hz_1_rep_2,
'fitmono_EPSC1st_platz_NaN_10Hz_1_rep_2'


CurveFit dblexp_XOffset EPSC1st_platz_NaN_10Hz_1_rep_2 [V_minRowLoc,V_minRowLoc+200]
/D

FitsEPSC_platz_NaN_10Hz_1_rep_2[2]=W_Coef[1]

FitsEPSC_platz_NaN_10Hz_1_rep_2[3]=W_Coef[2]

FitsEPSC_platz_NaN_10Hz_1_rep_2[4]=W_Coef[3]

FitsEPSC_platz_NaN_10Hz_1_rep_2[5]=W_Coef[4]

FitsEPSC_platz_NaN_10Hz_1_rep_2[6]=(W_coef[2]*(-1*W_coef[1])+W_coef[4]*(-
1*W_coef[3]))/((-1*W_coef[1])+(-1*W_coef[3]))


edit FitsEPSC_platz_NaN_10Hz_1_rep_0, FitsEPSC_platz_NaN_10Hz_1_rep_1,
FitsEPSC_platz_NaN_10Hz_1_rep_2

end


/////////-------------------------------------------------------------------------------///////////

Function GettheEPCSs_50Hz()

Rep1_50()

Rep2_50()

Rep3_50()

end


function Rep1_50()

Wave platz_NaN_50_rep_0 ,platz_NaN_50_rep_1 ,platz_NaN_50_rep_2, W_Coef,
FitsEPSC_platz_NaN_50Hz_1_rep_0, fit_EPSC1st_platz_NaN_50Hz_1_rep_0,
fitmono_EPSC1st_platz_NaN_50Hz_1_rep_0, EPSC1st_platz_NaN_50Hz_1_rep_0

Variable Beginning, Ending


Make/O/N=5 W_Coef


Beginning=1849
```

```
Ending=2641

Duplicate/R= [Beginning,Ending] platz_NaN_50_rep_0,EPSC1st_platz_NaN_50Hz_1_rep_0

Display EPSC1st_platz_NaN_50Hz_1_rep_0

make/o/n=7 FitsEPSC_platz_NaN_50Hz_1_rep_0

Wavestats EPSC1st_platz_NaN_50Hz_1_rep_0

CurveFit exp_XOffset EPSC1st_platz_NaN_50Hz_1_rep_0 [V_minRowLoc,V_minRowLoc+200] /D

FitsEPSC_platz_NaN_50Hz_1_rep_0[0]=W_Coef[1]

FitsEPSC_platz_NaN_50Hz_1_rep_0[1]=W_Coef[2]

wave fit_EPSC1st_platz_NaN_50Hz_1_rep_0, fitmono_EPSC1st_platz_NaN_50Hz_1_rep_0

Duplicate/O fit_EPSC1st_platz_NaN_50Hz_1_rep_0,
'fitmono_EPSC1st_platz_NaN_50Hz_1_rep_0'


CurveFit dblexp_XOffset EPSC1st_platz_NaN_50Hz_1_rep_0 [V_minRowLoc,V_minRowLoc+200]
/D

FitsEPSC_platz_NaN_50Hz_1_rep_0[2]=W_Coef[1]

FitsEPSC_platz_NaN_50Hz_1_rep_0[3]=W_Coef[2]

FitsEPSC_platz_NaN_50Hz_1_rep_0[4]=W_Coef[3]

FitsEPSC_platz_NaN_50Hz_1_rep_0[5]=W_Coef[4]

FitsEPSC_platz_NaN_50Hz_1_rep_0[6]=(W_coef[2]*(-1*W_coef[1])+W_coef[4]*(-
1*W_coef[3]))/((-1*W_coef[1])+(-1*W_coef[3]))


end


function Rep2_50()

Wave platz_NaN_50_rep_0,platz_NaN_50_rep_1,platz_NaN_50_rep_2,W_Coef,
FitsEPSC_platz_NaN_50Hz_1_rep_0,fit_EPSC1st_platz_NaN_50Hz_1_rep_1,
fitmono_EPSC1st_platz_NaN_50Hz_1_rep_1

Variable Beginning, Ending

Beginning=1849

Ending=2641

Duplicate/R= [Beginning,Ending] platz_NaN_50_rep_1,EPSC1st_platz_NaN_50Hz_1_rep_1

Display EPSC1st_platz_NaN_50Hz_1_rep_1

make/o/n=7 FitsEPSC_platz_NaN_50Hz_1_rep_1
```

```
Wavestats EPSC1st_platz_NaN_50Hz_1_rep_1

CurveFit exp_XOffset EPSC1st_platz_NaN_50Hz_1_rep_1 [V_minRowLoc,V_minRowLoc+200] /D

FitsEPSC_platz_NaN_50Hz_1_rep_1[0]=W_Coef[1]

FitsEPSC_platz_NaN_50Hz_1_rep_1[1]=W_Coef[2]

wave fit_EPSC1st_platz_NaN_50Hz_1_rep_1, fitmono_EPSC1st_platz_NaN_50Hz_1_rep_1

Duplicate/O fit_EPSC1st_platz_NaN_50Hz_1_rep_1,
'fitmono_EPSC1st_platz_NaN_50Hz_1_rep_1'


CurveFit dblexp_XOffset EPSC1st_platz_NaN_50Hz_1_rep_1 [V_minRowLoc,V_minRowLoc+200]
/D

FitsEPSC_platz_NaN_50Hz_1_rep_1[2]=W_Coef[1]

FitsEPSC_platz_NaN_50Hz_1_rep_1[3]=W_Coef[2]

FitsEPSC_platz_NaN_50Hz_1_rep_1[4]=W_Coef[3]

FitsEPSC_platz_NaN_50Hz_1_rep_1[5]=W_Coef[4]

FitsEPSC_platz_NaN_50Hz_1_rep_1[6]=(W_coef[2]*(-1*W_coef[1])+W_coef[4]*(-
1*W_coef[3]))/((-1*W_coef[1])+(-1*W_coef[3]))


end


function Rep3_50()

Wave platz_NaN_50_rep_0,platz_NaN_50_rep_1,platz_NaN_50_rep_2,W_Coef,
FitsEPSC_platz_NaN_50Hz_1_rep_2,fit_EPSC1st_platz_NaN_50Hz_1_rep_2,
fitmono_EPSC1st_platz_NaN_50Hz_1_rep_2, FitsEPSC_platz_NaN_50Hz_1_rep_0,
FitsEPSC_platz_NaN_50Hz_1_rep_1

Variable Beginning, Ending

Beginning=1849

Ending=2641

Duplicate/R= [Beginning,Ending] platz_NaN_50_rep_2,EPSC1st_platz_NaN_50Hz_1_rep_2

Display EPSC1st_platz_NaN_50Hz_1_rep_2

make/o/n=7 FitsEPSC_platz_NaN_50Hz_1_rep_2

Wavestats EPSC1st_platz_NaN_50Hz_1_rep_2

CurveFit exp_XOffset EPSC1st_platz_NaN_50Hz_1_rep_2 [V_minRowLoc,V_minRowLoc+200] /D

FitsEPSC_platz_NaN_50Hz_1_rep_2[0]=W_Coef[1]

FitsEPSC_platz_NaN_50Hz_1_rep_2[1]=W_Coef[2]
```

```
wave fit_EPSC1st_platz_NaN_50Hz_1_rep_2, fitmono_EPSC1st_platz_NaN_50Hz_1_rep_2

Duplicate/O fit_EPSC1st_platz_NaN_50Hz_1_rep_2,
'fitmono_EPSC1st_platz_NaN_50Hz_1_rep_2'


CurveFit dblexp_XOffset EPSC1st_platz_NaN_50Hz_1_rep_2 [V_minRowLoc,V_minRowLoc+200]
/D

FitsEPSC_platz_NaN_50Hz_1_rep_2[2]=W_Coef[1]

FitsEPSC_platz_NaN_50Hz_1_rep_2[3]=W_Coef[2]

FitsEPSC_platz_NaN_50Hz_1_rep_2[4]=W_Coef[3]

FitsEPSC_platz_NaN_50Hz_1_rep_2[5]=W_Coef[4]

FitsEPSC_platz_NaN_50Hz_1_rep_2[6]=(W_coef[2]*(-1*W_coef[1])+W_coef[4]*(-
1*W_coef[3]))/((-1*W_coef[1])+(-1*W_coef[3]))


edit FitsEPSC_platz_NaN_50Hz_1_rep_0, FitsEPSC_platz_NaN_50Hz_1_rep_1,
FitsEPSC_platz_NaN_50Hz_1_rep_2

end


/////////---------------------------------------------------------------------------------///////////

Function GettheEPCSs_100Hz()

Rep1_100()

Rep2_100()

Rep3_100()

end


function Rep1_100()

Wave platz_NaN_100_rep_0,platz_NaN_100_rep_1,platz_NaN_100_rep_2,W_Coef,
FitsEPSC_platz_NaN_100Hz_1_rep_0,fit_EPSC1st_platz_NaN_100Hz_1_rep_0,
fitmono_EPSC1st_platz_NaN_100Hz_1_rep_0, EPSC1st_platz_NaN_100Hz_1_rep_0

Variable Beginning, Ending


Make/O/N=5 W_Coef


Beginning=1858

Ending=2220
```

```
Duplicate/R= [Beginning,Ending] platz_NaN_100_rep_0,EPSC1st_platz_NaN_100Hz_1_rep_0

Display EPSC1st_platz_NaN_100Hz_1_rep_0

make/o/n=7 FitsEPSC_platz_NaN_100Hz_1_rep_0

Wavestats EPSC1st_platz_NaN_100Hz_1_rep_0

CurveFit exp_XOffset EPSC1st_platz_NaN_100Hz_1_rep_0 [V_minRowLoc,V_minRowLoc+300]
/D

FitsEPSC_platz_NaN_100Hz_1_rep_0[0]=W_Coef[1]

FitsEPSC_platz_NaN_100Hz_1_rep_0[1]=W_Coef[2]

wave fit_EPSC1st_platz_NaN_100Hz_1_rep_0, fitmono_EPSC1st_platz_NaN_100Hz_1_rep_0

Duplicate/O fit_EPSC1st_platz_NaN_100Hz_1_rep_0,
'fitmono_EPSC1st_platz_NaN_100Hz_1_rep_0'


CurveFit dblexp_XOffset EPSC1st_platz_NaN_100Hz_1_rep_0
[V_minRowLoc,V_minRowLoc+300] /D

FitsEPSC_platz_NaN_100Hz_1_rep_0[2]=W_Coef[1]

FitsEPSC_platz_NaN_100Hz_1_rep_0[3]=W_Coef[2]

FitsEPSC_platz_NaN_100Hz_1_rep_0[4]=W_Coef[3]

FitsEPSC_platz_NaN_100Hz_1_rep_0[5]=W_Coef[4]

FitsEPSC_platz_NaN_100Hz_1_rep_0[6]=(W_coef[2]*(-1*W_coef[1])+W_coef[4]*(-
1*W_coef[3]))/((-1*W_coef[1])+(-1*W_coef[3]))


end


function Rep2_100()

Wave platz_NaN_100_rep_0,platz_NaN_100_rep_1,platz_NaN_100_rep_2,W_Coef,
FitsEPSC_platz_NaN_100Hz_1_rep_0,fit_EPSC1st_platz_NaN_100Hz_1_rep_1,
fitmono_EPSC1st_platz_NaN_100Hz_1_rep_1

Variable Beginning, Ending

Beginning=1858

Ending=2220

Duplicate/R= [Beginning,Ending] platz_NaN_100_rep_1,EPSC1st_platz_NaN_100Hz_1_rep_1

Display EPSC1st_platz_NaN_100Hz_1_rep_1

make/o/n=7 FitsEPSC_platz_NaN_100Hz_1_rep_1

Wavestats EPSC1st_platz_NaN_100Hz_1_rep_1
```

```
CurveFit exp_XOffset EPSC1st_platz_NaN_100Hz_1_rep_1 [V_minRowLoc,V_minRowLoc+300] /D

FitsEPSC_platz_NaN_100Hz_1_rep_1[0]=W_Coef[1]

FitsEPSC_platz_NaN_100Hz_1_rep_1[1]=W_Coef[2]

wave fit_EPSC1st_platz_NaN_100Hz_1_rep_1, fitmono_EPSC1st_platz_NaN_100Hz_1_rep_1

Duplicate/O fit_EPSC1st_platz_NaN_100Hz_1_rep_1, 'fitmono_EPSC1st_platz_NaN_100Hz_1_rep_1'


CurveFit dblexp_XOffset EPSC1st_platz_NaN_100Hz_1_rep_1 [V_minRowLoc,V_minRowLoc+300] /D

FitsEPSC_platz_NaN_100Hz_1_rep_1[2]=W_Coef[1]

FitsEPSC_platz_NaN_100Hz_1_rep_1[3]=W_Coef[2]

FitsEPSC_platz_NaN_100Hz_1_rep_1[4]=W_Coef[3]

FitsEPSC_platz_NaN_100Hz_1_rep_1[5]=W_Coef[4]

FitsEPSC_platz_NaN_100Hz_1_rep_1[6]=(W_coef[2]*(-1*W_coef[1])+W_coef[4]*(-1*W_coef[3]))/((-1*W_coef[1])+(-1*W_coef[3]))


end


function Rep3_100()

Wave platz_NaN_100_rep_0,platz_NaN_100_rep_1,platz_NaN_100_rep_2,W_Coef, FitsEPSC_platz_NaN_100Hz_1_rep_2,fit_EPSC1st_platz_NaN_100Hz_1_rep_2, fitmono_EPSC1st_platz_NaN_100Hz_1_rep_2, FitsEPSC_platz_NaN_100Hz_1_rep_1, FitsEPSC_platz_NaN_100Hz_1_rep_0

Variable Beginning, Ending

Beginning=1858

Ending=2220

Duplicate/R= [Beginning,Ending] platz_NaN_100_rep_2,EPSC1st_platz_NaN_100Hz_1_rep_2

Display EPSC1st_platz_NaN_100Hz_1_rep_2

make/o/n=7 FitsEPSC_platz_NaN_100Hz_1_rep_2

Wavestats EPSC1st_platz_NaN_100Hz_1_rep_2

CurveFit exp_XOffset EPSC1st_platz_NaN_100Hz_1_rep_2 [V_minRowLoc,V_minRowLoc+300] /D

FitsEPSC_platz_NaN_100Hz_1_rep_2[0]=W_Coef[1]

FitsEPSC_platz_NaN_100Hz_1_rep_2[1]=W_Coef[2]
```

```
wave fit_EPSC1st_platz_NaN_100Hz_1_rep_2, fitmono_EPSC1st_platz_NaN_100Hz_1_rep_2

Duplicate/O fit_EPSC1st_platz_NaN_100Hz_1_rep_2,
'fitmono_EPSC1st_platz_NaN_100Hz_1_rep_2'


CurveFit dblexp_XOffset EPSC1st_platz_NaN_100Hz_1_rep_2
[V_minRowLoc,V_minRowLoc+300] /D

FitsEPSC_platz_NaN_100Hz_1_rep_2[2]=W_Coef[1]

FitsEPSC_platz_NaN_100Hz_1_rep_2[3]=W_Coef[2]

FitsEPSC_platz_NaN_100Hz_1_rep_2[4]=W_Coef[3]

FitsEPSC_platz_NaN_100Hz_1_rep_2[5]=W_Coef[4]

FitsEPSC_platz_NaN_100Hz_1_rep_2[6]=(W_coef[2]*(-1*W_coef[1])+W_coef[4]*(-
1*W_coef[3]))/((-1*W_coef[1])+(-1*W_coef[3]))


edit FitsEPSC_platz_NaN_100Hz_1_rep_0, FitsEPSC_platz_NaN_100Hz_1_rep_1,
FitsEPSC_platz_NaN_100Hz_1_rep_2

end


/////////---------------------------------------------------------------------------------///////////

Function GettheEPCSs_200Hz()

Rep1_200()

Rep2_200()

Rep3_200()

end


function Rep1_200()

Wave platz_NaN_200_rep_0,platz_NaN_200_rep_1,platz_NaN_200_rep_2,W_Coef,
FitsEPSC_platz_NaN_200Hz_1_rep_0,fit_EPSC1st_platz_NaN_200Hz_1_rep_0,
fitmono_EPSC1st_platz_NaN_200Hz_1_rep_0, EPSC1st_platz_NaN_200Hz_1_rep_0

Variable Beginning, Ending


Make/O/N=5 W_Coef


Beginning=1849

Ending=2037
```

```
Duplicate/R= [Beginning,Ending] platz_NaN_200_rep_0,EPSC1st_platz_NaN_200Hz_1_rep_0

Display EPSC1st_platz_NaN_200Hz_1_rep_0

make/o/n=7 FitsEPSC_platz_NaN_200Hz_1_rep_0

Wavestats EPSC1st_platz_NaN_200Hz_1_rep_0

CurveFit exp_XOffset EPSC1st_platz_NaN_200Hz_1_rep_0 [V_minRowLoc,V_minRowLoc+160]
/D

FitsEPSC_platz_NaN_200Hz_1_rep_0[0]=W_Coef[1]

FitsEPSC_platz_NaN_200Hz_1_rep_0[1]=W_Coef[2]

wave fit_EPSC1st_platz_NaN_200Hz_1_rep_0, fitmono_EPSC1st_platz_NaN_200Hz_1_rep_0

Duplicate/O fit_EPSC1st_platz_NaN_200Hz_1_rep_0,
'fitmono_EPSC1st_platz_NaN_200Hz_1_rep_0'


CurveFit dblexp_XOffset EPSC1st_platz_NaN_200Hz_1_rep_0
[V_minRowLoc,V_minRowLoc+160] /D

FitsEPSC_platz_NaN_200Hz_1_rep_0[2]=W_Coef[1]

FitsEPSC_platz_NaN_200Hz_1_rep_0[3]=W_Coef[2]

FitsEPSC_platz_NaN_200Hz_1_rep_0[4]=W_Coef[3]

FitsEPSC_platz_NaN_200Hz_1_rep_0[5]=W_Coef[4]

FitsEPSC_platz_NaN_200Hz_1_rep_0[6]=(W_coef[2]*(-1*W_coef[1])+W_coef[4]*(-
1*W_coef[3]))/((-1*W_coef[1])+(-1*W_coef[3]))


end


function Rep2_200()

Wave platz_NaN_200_rep_0,platz_NaN_200_rep_1,platz_NaN_200_rep_2,W_Coef,
FitsEPSC_platz_NaN_200Hz_1_rep_0,fit_EPSC1st_platz_NaN_200Hz_1_rep_1,
fitmono_EPSC1st_platz_NaN_200Hz_1_rep_1

Variable Beginning, Ending

Beginning=1849

Ending=2037

Duplicate/R= [Beginning,Ending] platz_NaN_200_rep_1,EPSC1st_platz_NaN_200Hz_1_rep_1

Display EPSC1st_platz_NaN_200Hz_1_rep_1

make/o/n=7 FitsEPSC_platz_NaN_200Hz_1_rep_1

Wavestats EPSC1st_platz_NaN_200Hz_1_rep_1
```

```
CurveFit exp_XOffset EPSC1st_platz_NaN_200Hz_1_rep_1 [V_minRowLoc,V_minRowLoc+160]
/D

FitsEPSC_platz_NaN_200Hz_1_rep_1[0]=W_Coef[1]

FitsEPSC_platz_NaN_200Hz_1_rep_1[1]=W_Coef[2]

wave fit_EPSC1st_platz_NaN_200Hz_1_rep_1, fitmono_EPSC1st_platz_NaN_200Hz_1_rep_1

Duplicate/O fit_EPSC1st_platz_NaN_200Hz_1_rep_1,
'fitmono_EPSC1st_platz_NaN_200Hz_1_rep_1'


CurveFit dblexp_XOffset EPSC1st_platz_NaN_200Hz_1_rep_1
[V_minRowLoc,V_minRowLoc+160] /D

FitsEPSC_platz_NaN_200Hz_1_rep_1[2]=W_Coef[1]

FitsEPSC_platz_NaN_200Hz_1_rep_1[3]=W_Coef[2]

FitsEPSC_platz_NaN_200Hz_1_rep_1[4]=W_Coef[3]

FitsEPSC_platz_NaN_200Hz_1_rep_1[5]=W_Coef[4]

FitsEPSC_platz_NaN_200Hz_1_rep_1[6]=(W_coef[2]*(-1*W_coef[1])+W_coef[4]*(-
1*W_coef[3]))/((-1*W_coef[1])+(-1*W_coef[3]))


end


function Rep3_200()

Wave platz_NaN_200_rep_0,platz_NaN_200_rep_1,platz_NaN_200_rep_2,W_Coef,
FitsEPSC_platz_NaN_200Hz_1_rep_2,fit_EPSC1st_platz_NaN_200Hz_1_rep_2,
fitmono_EPSC1st_platz_NaN_200Hz_1_rep_2, FitsEPSC_platz_NaN_200Hz_1_rep_0,
FitsEPSC_platz_NaN_200Hz_1_rep_1

Variable Beginning, Ending

Beginning=1849

Ending=2037

Duplicate/R= [Beginning,Ending] platz_NaN_200_rep_2,EPSC1st_platz_NaN_200Hz_1_rep_2

Display EPSC1st_platz_NaN_200Hz_1_rep_2

make/o/n=7 FitsEPSC_platz_NaN_200Hz_1_rep_2

Wavestats EPSC1st_platz_NaN_200Hz_1_rep_2

CurveFit exp_XOffset EPSC1st_platz_NaN_200Hz_1_rep_2 [V_minRowLoc,V_minRowLoc+160]
/D

FitsEPSC_platz_NaN_200Hz_1_rep_2[0]=W_Coef[1]

FitsEPSC_platz_NaN_200Hz_1_rep_2[1]=W_Coef[2]
```

```
wave fit_EPSC1st_platz_NaN_200Hz_1_rep_2, fitmono_EPSC1st_platz_NaN_200Hz_1_rep_2

Duplicate/O fit_EPSC1st_platz_NaN_200Hz_1_rep_2,
'fitmono_EPSC1st_platz_NaN_200Hz_1_rep_2'


CurveFit dblexp_XOffset EPSC1st_platz_NaN_200Hz_1_rep_2
[V_minRowLoc,V_minRowLoc+160] /D

FitsEPSC_platz_NaN_200Hz_1_rep_2[2]=W_Coef[1]

FitsEPSC_platz_NaN_200Hz_1_rep_2[3]=W_Coef[2]

FitsEPSC_platz_NaN_200Hz_1_rep_2[4]=W_Coef[3]

FitsEPSC_platz_NaN_200Hz_1_rep_2[5]=W_Coef[4]

FitsEPSC_platz_NaN_200Hz_1_rep_2[6]=(W_coef[2]*(-1*W_coef[1])+W_coef[4]*(-
1*W_coef[3]))/((-1*W_coef[1])+(-1*W_coef[3]))


edit FitsEPSC_platz_NaN_200Hz_1_rep_0, FitsEPSC_platz_NaN_200Hz_1_rep_1,
FitsEPSC_platz_NaN_200Hz_1_rep_2

end


/////////-------------------------------------------------------------------------------///////////

Function GettheEPCSs_300Hz()

Rep1_300()

Rep2_300()

Rep3_300()

end


function Rep1_300()

Wave Platz_NaN_300_rep_0,Platz_NaN_300_rep_1,Platz_NaN_300_rep_2,W_Coef,
FitsEPSC_platz_NaN_300Hz_1_rep_0,fit_EPSC1st_platz_NaN_300Hz_1_rep_0,
fitmono_EPSC1st_platz_NaN_300Hz_1_rep_0, EPSC1st_platz_NaN_300Hz_1_rep_0

Variable Beginning, Ending


Make/O/N=5 W_Coef


Beginning=1849

Ending=1951
```

```
Duplicate/R= [Beginning,Ending] Platz_NaN_300_rep_0,EPSC1st_platz_NaN_300Hz_1_rep_0

Display EPSC1st_platz_NaN_300Hz_1_rep_0

make/o/n=7 FitsEPSC_platz_NaN_300Hz_1_rep_0

Wavestats EPSC1st_platz_NaN_300Hz_1_rep_0

CurveFit exp_XOffset EPSC1st_platz_NaN_300Hz_1_rep_0 [V_minRowLoc,V_minRowLoc+80] /D

FitsEPSC_platz_NaN_300Hz_1_rep_0[0]=W_Coef[1]

FitsEPSC_platz_NaN_300Hz_1_rep_0[1]=W_Coef[2]

wave fit_EPSC1st_platz_NaN_300Hz_1_rep_0, fitmono_EPSC1st_platz_NaN_300Hz_1_rep_0

Duplicate/O fit_EPSC1st_platz_NaN_300Hz_1_rep_0,
'fitmono_EPSC1st_platz_NaN_300Hz_1_rep_0'


CurveFit dblexp_XOffset EPSC1st_platz_NaN_300Hz_1_rep_0 [V_minRowLoc,V_minRowLoc+80]
/D

FitsEPSC_platz_NaN_300Hz_1_rep_0[2]=W_Coef[1]

FitsEPSC_platz_NaN_300Hz_1_rep_0[3]=W_Coef[2]

FitsEPSC_platz_NaN_300Hz_1_rep_0[4]=W_Coef[3]

FitsEPSC_platz_NaN_300Hz_1_rep_0[5]=W_Coef[4]

FitsEPSC_platz_NaN_300Hz_1_rep_0[6]=(W_coef[2]*(-1*W_coef[1])+W_coef[4]*(-
1*W_coef[3]))/((-1*W_coef[1])+(-1*W_coef[3]))


end


function Rep2_300()

Wave Platz_NaN_300_rep_0,Platz_NaN_300_rep_1,Platz_NaN_300_rep_2,W_Coef,
FitsEPSC_platz_NaN_300Hz_1_rep_0,fit_EPSC1st_platz_NaN_300Hz_1_rep_1,
fitmono_EPSC1st_platz_NaN_300Hz_1_rep_1, EPSC1st_platz_NaN_300Hz_1_rep_0

Variable Beginning, Ending

Beginning=1849

Ending=1951

Duplicate/R= [Beginning,Ending] Platz_NaN_300_rep_1,EPSC1st_platz_NaN_300Hz_1_rep_1

Display EPSC1st_platz_NaN_300Hz_1_rep_1

make/o/n=7 FitsEPSC_platz_NaN_300Hz_1_rep_1

Wavestats EPSC1st_platz_NaN_300Hz_1_rep_1
```

```
CurveFit exp_XOffset EPSC1st_platz_NaN_300Hz_1_rep_1 [V_minRowLoc,V_minRowLoc+80] /D

FitsEPSC_platz_NaN_300Hz_1_rep_1[0]=W_Coef[1]

FitsEPSC_platz_NaN_300Hz_1_rep_1[1]=W_Coef[2]

wave fit_EPSC1st_platz_NaN_300Hz_1_rep_1, fitmono_EPSC1st_platz_NaN_300Hz_1_rep_1

Duplicate/O fit_EPSC1st_platz_NaN_300Hz_1_rep_1,
'fitmono_EPSC1st_platz_NaN_300Hz_1_rep_1'


CurveFit dblexp_XOffset EPSC1st_platz_NaN_300Hz_1_rep_1 [V_minRowLoc,V_minRowLoc+80]
/D

FitsEPSC_platz_NaN_300Hz_1_rep_1[2]=W_Coef[1]

FitsEPSC_platz_NaN_300Hz_1_rep_1[3]=W_Coef[2]

FitsEPSC_platz_NaN_300Hz_1_rep_1[4]=W_Coef[3]

FitsEPSC_platz_NaN_300Hz_1_rep_1[5]=W_Coef[4]

FitsEPSC_platz_NaN_300Hz_1_rep_1[6]=(W_coef[2]*(-1*W_coef[1])+W_coef[4]*(-
1*W_coef[3]))/((-1*W_coef[1])+(-1*W_coef[3]))


end


function Rep3_300()

Wave Platz_NaN_300_rep_0,Platz_NaN_300_rep_1,Platz_NaN_300_rep_2,W_Coef,
FitsEPSC_platz_NaN_300Hz_1_rep_2,fit_EPSC1st_platz_NaN_10Hz_1_rep_2,
fitmono_EPSC1st_platz_NaN_300Hz_1_rep_2, FitsEPSC_platz_NaN_300Hz_1_rep_0,
FitsEPSC_platz_NaN_300Hz_1_rep_1

Variable Beginning, Ending

Beginning=1849

Ending=1951

Duplicate/R= [Beginning,Ending] Platz_NaN_300_rep_2,EPSC1st_platz_NaN_300Hz_1_rep_2

Display EPSC1st_platz_NaN_300Hz_1_rep_2

make/o/n=7 FitsEPSC_platz_NaN_300Hz_1_rep_2

Wavestats EPSC1st_platz_NaN_300Hz_1_rep_2

CurveFit exp_XOffset EPSC1st_platz_NaN_300Hz_1_rep_2 [V_minRowLoc,V_minRowLoc+80] /D

FitsEPSC_platz_NaN_300Hz_1_rep_2[0]=W_Coef[1]

FitsEPSC_platz_NaN_300Hz_1_rep_2[1]=W_Coef[2]

wave fit_EPSC1st_platz_NaN_300Hz_1_rep_2, fitmono_EPSC1st_platz_NaN_300Hz_1_rep_2
```

```
Duplicate/O fit_EPSC1st_platz_NaN_300Hz_1_rep_2,
'fitmono_EPSC1st_platz_NaN_300Hz_1_rep_2'


CurveFit dblexp_XOffset EPSC1st_platz_NaN_300Hz_1_rep_2 [V_minRowLoc,V_minRowLoc+80]
/D

FitsEPSC_platz_NaN_300Hz_1_rep_2[2]=W_Coef[1]

FitsEPSC_platz_NaN_300Hz_1_rep_2[3]=W_Coef[2]

FitsEPSC_platz_NaN_300Hz_1_rep_2[4]=W_Coef[3]

FitsEPSC_platz_NaN_300Hz_1_rep_2[5]=W_Coef[4]

FitsEPSC_platz_NaN_300Hz_1_rep_2[6]=(W_coef[2]*(-1*W_coef[1])+W_coef[4]*(-
1*W_coef[3]))/((-1*W_coef[1])+(-1*W_coef[3]))


edit FitsEPSC_platz_NaN_300Hz_1_rep_0, FitsEPSC_platz_NaN_300Hz_1_rep_1,
FitsEPSC_platz_NaN_300Hz_1_rep_2

end


/////////------------------------------------------------------------------------------///////////

Function GettheEPCSs_400Hz()

Rep1_400()

Rep2_400()

Rep3_400()

end


function Rep1_400()

Wave platz_NaN_400_rep_0,platz_NaN_400_rep_1,platz_NaN_400_rep_2,W_Coef,
FitsEPSC_platz_NaN_400Hz_1_rep_0,fit_EPSC1st_platz_NaN_400Hz_1_rep_0,
fitmono_EPSC1st_platz_NaN_400Hz_1_rep_0, EPSC1st_platz_NaN_400Hz_1_rep_0

Variable Beginning, Ending


Make/O/N=5 W_Coef


Beginning=1849

Ending=1906

Duplicate/R= [Beginning,Ending] platz_NaN_400_rep_0,EPSC1st_platz_NaN_400Hz_1_rep_0
```

```
Display EPSC1st_platz_NaN_400Hz_1_rep_0

make/o/n=7 FitsEPSC_platz_NaN_400Hz_1_rep_0

Wavestats EPSC1st_platz_NaN_400Hz_1_rep_0

CurveFit exp_XOffset EPSC1st_platz_NaN_400Hz_1_rep_0 [V_minRowLoc,V_minRowLoc+40] /D

FitsEPSC_platz_NaN_400Hz_1_rep_0[0]=W_Coef[1]

FitsEPSC_platz_NaN_400Hz_1_rep_0[1]=W_Coef[2]

wave fit_EPSC1st_platz_NaN_400Hz_1_rep_0, fitmono_EPSC1st_platz_NaN_400Hz_1_rep_0

Duplicate/O fit_EPSC1st_platz_NaN_400Hz_1_rep_0,
'fitmono_EPSC1st_platz_NaN_400Hz_1_rep_0'


CurveFit dblexp_XOffset EPSC1st_platz_NaN_400Hz_1_rep_0 [V_minRowLoc,V_minRowLoc+40]
/D

FitsEPSC_platz_NaN_400Hz_1_rep_0[2]=W_Coef[1]

FitsEPSC_platz_NaN_400Hz_1_rep_0[3]=W_Coef[2]

FitsEPSC_platz_NaN_400Hz_1_rep_0[4]=W_Coef[3]

FitsEPSC_platz_NaN_400Hz_1_rep_0[5]=W_Coef[4]

FitsEPSC_platz_NaN_400Hz_1_rep_0[6]=(W_coef[2]*(-1*W_coef[1])+W_coef[4]*(-
1*W_coef[3]))/((-1*W_coef[1])+(-1*W_coef[3]))



end


function Rep2_400()

Wave platz_NaN_400_rep_0,platz_NaN_400_rep_1,platz_NaN_400_1_rep_2,W_Coef,
FitsEPSC_platz_NaN_400Hz_1_rep_0,fit_EPSC1st_platz_NaN_400Hz_1_rep_1,
fitmono_EPSC1st_platz_NaN_400Hz_1_rep_1

Variable Beginning, Ending

Beginning=1849

Ending=1906

Duplicate/R= [Beginning,Ending] platz_NaN_400_rep_1,EPSC1st_platz_NaN_400Hz_1_rep_1

Display EPSC1st_platz_NaN_400Hz_1_rep_1

make/o/n=7 FitsEPSC_platz_NaN_400Hz_1_rep_1

Wavestats EPSC1st_platz_NaN_400Hz_1_rep_1
```

CurveFit exp_XOffset EPSC1st_platz_NaN_400Hz_1_rep_1 [V_minRowLoc,V_minRowLoc+40] /D

FitsEPSC_platz_NaN_400Hz_1_rep_1[0]=W_Coef[1]

FitsEPSC_platz_NaN_400Hz_1_rep_1[1]=W_Coef[2]

wave fit_EPSC1st_platz_NaN_400Hz_1_rep_1, fitmono_EPSC1st_platz_NaN_400Hz_1_rep_1

Duplicate/O fit_EPSC1st_platz_NaN_400Hz_1_rep_1, 'fitmono_EPSC1st_platz_NaN_400Hz_1_rep_1'

CurveFit dblexp_XOffset EPSC1st_platz_NaN_400Hz_1_rep_1 [V_minRowLoc,V_minRowLoc+40] /D

FitsEPSC_platz_NaN_400Hz_1_rep_1[2]=W_Coef[1]

FitsEPSC_platz_NaN_400Hz_1_rep_1[3]=W_Coef[2]

FitsEPSC_platz_NaN_400Hz_1_rep_1[4]=W_Coef[3]

FitsEPSC_platz_NaN_400Hz_1_rep_1[5]=W_Coef[4]

FitsEPSC_platz_NaN_400Hz_1_rep_1[6]=(W_coef[2]*(-1*W_coef[1])+W_coef[4]*(-1*W_coef[3]))/((-1*W_coef[1])+(-1*W_coef[3]))

end

function Rep3_400()

Wave platz_NaN_400_rep_0,platz_NaN_400_rep_1,platz_NaN_400_rep_2,W_Coef, FitsEPSC_platz_NaN_400Hz_1_rep_2,fit_EPSC1st_platz_NaN_400Hz_1_rep_2, fitmono_EPSC1st_platz_NaN_400Hz_1_rep_2, FitsEPSC_platz_NaN_400Hz_1_rep_0, FitsEPSC_platz_NaN_400Hz_1_rep_1

Variable Beginning, Ending

Beginning=1849

Ending=1906

Duplicate/R= [Beginning,Ending] platz_NaN_400_rep_2,EPSC1st_platz_NaN_400Hz_1_rep_2

Display EPSC1st_platz_NaN_400Hz_1_rep_2

make/o/n=7 FitsEPSC_platz_NaN_400Hz_1_rep_2

Wavestats EPSC1st_platz_NaN_400Hz_1_rep_2

CurveFit exp_XOffset EPSC1st_platz_NaN_400Hz_1_rep_2 [V_minRowLoc,V_minRowLoc+40] /D

FitsEPSC_platz_NaN_400Hz_1_rep_2[0]=W_Coef[1]

FitsEPSC_platz_NaN_400Hz_1_rep_2[1]=W_Coef[2]

```
wave fit_EPSC1st_platz_NaN_400Hz_1_rep_2, fitmono_EPSC1st_platz_NaN_400Hz_1_rep_2

Duplicate/O fit_EPSC1st_platz_NaN_400Hz_1_rep_2,
'fitmono_EPSC1st_platz_NaN_400Hz_1_rep_2'


CurveFit dblexp_XOffset EPSC1st_platz_NaN_400Hz_1_rep_2 [V_minRowLoc,V_minRowLoc+40]
/D

FitsEPSC_platz_NaN_400Hz_1_rep_2[2]=W_Coef[1]

FitsEPSC_platz_NaN_400Hz_1_rep_2[3]=W_Coef[2]

FitsEPSC_platz_NaN_400Hz_1_rep_2[4]=W_Coef[3]

FitsEPSC_platz_NaN_400Hz_1_rep_2[5]=W_Coef[4]

FitsEPSC_platz_NaN_400Hz_1_rep_2[6]=(W_coef[2]*(-1*W_coef[1])+W_coef[4]*(-
1*W_coef[3]))/((-1*W_coef[1])+(-1*W_coef[3]))


edit FitsEPSC_platz_NaN_400Hz_1_rep_0, FitsEPSC_platz_NaN_400Hz_1_rep_1,
FitsEPSC_platz_NaN_400Hz_1_rep_2

end
```
