# Supplementary material for: Non-calyceal inputs gate the timing of calyx of Held evoked MNTB output
Source: Commun Biol. 2026 May 22;9:697. doi: 10.1038/s42003-026-10321-w (PMC13197447; doi:10.1038/s42003-026-10321-w)
Supplement: Supplementary file 6 — Supplementary code [file 42003_2026_10321_MOESM6_ESM.pdf]

```
#pragma TextEncoding = "UTF-8"
#pragma rtGlobals=3				// Use modern global access method and strict wave access
#pragma DefaultTab={3,20,4}		// Set default tab width in Igor Pro 9 and later

Menu "Macros"
  SubMenu "Fit the first EPSC"
    "100Hz", /Q, GettheEPCSs_100Hz()
    "200Hz", /Q, GettheEPCSs_200Hz()
    "300Hz", /Q, GettheEPCSs_300Hz()
    "400Hz", /Q, GettheEPCSs_400Hz()

  End
End
/////////--------------------------------------------------------------------------------///////////
Function GettheEPCSs_100Hz()
Rep1_100()
//Rep2_100()
//Rep3_100()
Wave FitsEPSC_copy0
edit FitsEPSC_copy0
//edit FitsEPSC_copy1
//edit FitsEPSC_copy2
end

function Rep1_100()
Wave avg_0_100_env,Copy_1_100,Copy_2_100,W_Coef, FitsEPSC_copy0,fit_EPSC1st_copy0, fitmono_EPSC1st_copy0, EPSC1st_copy0
Variable Beginning, Ending

Make/O/N=5 W_Coef
```

```
Beginning=2100235

Ending=2101181

Duplicate/R= [Beginning,Ending] avg_0_100_env,EPSC1st_copy0

Display EPSC1st_copy0

make/o/n=7 FitsEPSC_copy0

Wavestats EPSC1st_copy0

CurveFit exp_XOffset EPSC1st_copy0 [V_minRowLoc,V_minRowLoc+600] /D

FitsEPSC_copy0[0]=W_Coef[1]

FitsEPSC_copy0[1]=W_Coef[2]

wave fit_EPSC1st_copy0, fitmono_EPSC1st_copy0

Duplicate/O fit_EPSC1st_copy0, 'fitmono_EPSC1st_copy0'


CurveFit dblexp_XOffset EPSC1st_copy0 [V_minRowLoc,V_minRowLoc+600] /D

FitsEPSC_copy0[2]=W_Coef[1]

FitsEPSC_copy0[3]=W_Coef[2]

FitsEPSC_copy0[4]=W_Coef[3]

FitsEPSC_copy0[5]=W_Coef[4]

FitsEPSC_copy0[6]=(W_coef[2]*(-1*W_coef[1])+W_coef[4]*(-1*W_coef[3]))/((-1*W_coef[1])+(-
1*W_coef[3]))


end


function Rep2_100()

Wave Copy_0_100,Copy_1_100,Copy_2_100,W_Coef, FitsEPSC_copy0,fit_EPSC1st_copy1,
fitmono_EPSC1st_copy1

Variable Beginning, Ending

Beginning=100394

Ending=101163

Duplicate/R= [Beginning,Ending] Copy_1_100,EPSC1st_copy1

Display EPSC1st_copy1

make/o/n=7 FitsEPSC_copy1

Wavestats EPSC1st_copy1
```

```
CurveFit exp_XOffset EPSC1st_copy1 [V_minRowLoc,V_minRowLoc+600] /D
FitsEPSC_copy1[0]=W_Coef[1]
FitsEPSC_copy1[1]=W_Coef[2]
wave fit_EPSC1st_copy1, fitmono_EPSC1st_copy1
Duplicate/O fit_EPSC1st_copy1, 'fitmono_EPSC1st_copy1'

CurveFit dblexp_XOffset EPSC1st_copy1 [V_minRowLoc,V_minRowLoc+600] /D
FitsEPSC_copy1[2]=W_Coef[1]
FitsEPSC_copy1[3]=W_Coef[2]
FitsEPSC_copy1[4]=W_Coef[3]
FitsEPSC_copy1[5]=W_Coef[4]
FitsEPSC_copy1[6]=(W_coef[2]*(-1*W_coef[1])+W_coef[4]*(-1*W_coef[3]))/((-1*W_coef[1])+(-
1*W_coef[3]))

end

function Rep3_100()
Wave Copy_0_100,Copy_1_100,Copy_2_100,W_Coef, FitsEPSC_copy2,fit_EPSC1st_copy2,
fitmono_EPSC1st_copy2, FitsEPSC_copy1, FitsEPSC_copy0
Variable Beginning, Ending
Beginning=100394
Ending=101163
Duplicate/R= [Beginning,Ending] Copy_2_100,EPSC1st_copy2
Display EPSC1st_copy2
make/o/n=7 FitsEPSC_copy2
Wavestats EPSC1st_copy2
CurveFit exp_XOffset EPSC1st_copy2 [V_minRowLoc,V_minRowLoc+600] /D
FitsEPSC_copy2[0]=W_Coef[1]
FitsEPSC_copy2[1]=W_Coef[2]
wave fit_EPSC1st_copy2, fitmono_EPSC1st_copy2
Duplicate/O fit_EPSC1st_copy2, 'fitmono_EPSC1st_copy2'
```

```
CurveFit dblexp_XOffset EPSC1st_copy2 [V_minRowLoc,V_minRowLoc+600] /D
FitsEPSC_copy2[2]=W_Coef[1]
FitsEPSC_copy2[3]=W_Coef[2]
FitsEPSC_copy2[4]=W_Coef[3]
FitsEPSC_copy2[5]=W_Coef[4]
FitsEPSC_copy2[6]=(W_coef[2]*(-1*W_coef[1])+W_coef[4]*(-1*W_coef[3]))/((-1*W_coef[1])+(-1*W_coef[3]))

edit FitsEPSC_copy0, FitsEPSC_copy1, FitsEPSC_copy2
end

/////////---------------------------------------------------------------------------------///////////
Function GettheEPCSs_200Hz()
Rep1_200()
//Rep2_100()
//Rep3_100()
Wave FitsEPSC_copy0
edit FitsEPSC_copy0
//edit FitsEPSC_copy1
//edit FitsEPSC_copy2
end

function Rep1_200()
Wave avg_0_200_env,Copy_1_200,Copy_2_200,W_Coef, FitsEPSC_copy0,fit_EPSC1st_copy0, fitmono_EPSC1st_copy0, EPSC1st_copy0
Variable Beginning, Ending

Make/O/N=5 W_Coef

Beginning=2100163
Ending=2100734
Duplicate/R= [Beginning,Ending] avg_0_200_env,EPSC1st_copy0
```

```
Display EPSC1st_copy0

make/o/n=7 FitsEPSC_copy0

Wavestats EPSC1st_copy0

CurveFit exp_XOffset EPSC1st_copy0 [V_minRowLoc,V_minRowLoc+200] /D

FitsEPSC_copy0[0]=W_Coef[1]

FitsEPSC_copy0[1]=W_Coef[2]

wave fit_EPSC1st_copy0, fitmono_EPSC1st_copy0

Duplicate/O fit_EPSC1st_copy0, 'fitmono_EPSC1st_copy0'


CurveFit dblexp_XOffset EPSC1st_copy0 [V_minRowLoc,V_minRowLoc+200] /D

FitsEPSC_copy0[2]=W_Coef[1]

FitsEPSC_copy0[3]=W_Coef[2]

FitsEPSC_copy0[4]=W_Coef[3]

FitsEPSC_copy0[5]=W_Coef[4]

FitsEPSC_copy0[6]=(W_coef[2]*(-1*W_coef[1])+W_coef[4]*(-1*W_coef[3]))/((-1*W_coef[1])+(-
1*W_coef[3]))


end


function Rep2_200()

Wave  Copy_0_200,Copy_1_200,Copy_2_200,W_Coef, FitsEPSC_copy0,fit_EPSC1st_copy1,
fitmono_EPSC1st_copy1

Variable Beginning, Ending

Beginning=100370

Ending=100673

Duplicate/R= [Beginning,Ending] Copy_1_200,EPSC1st_copy1

Display EPSC1st_copy1

make/o/n=7 FitsEPSC_copy1

Wavestats EPSC1st_copy1

CurveFit exp_XOffset EPSC1st_copy1 [V_minRowLoc,V_minRowLoc+200] /D

FitsEPSC_copy1[0]=W_Coef[1]

FitsEPSC_copy1[1]=W_Coef[2]
```

```
wave fit_EPSC1st_copy1, fitmono_EPSC1st_copy1

Duplicate/O fit_EPSC1st_copy1, 'fitmono_EPSC1st_copy1'


CurveFit dblexp_XOffset EPSC1st_copy1 [V_minRowLoc,V_minRowLoc+200] /D

FitsEPSC_copy1[2]=W_Coef[1]

FitsEPSC_copy1[3]=W_Coef[2]

FitsEPSC_copy1[4]=W_Coef[3]

FitsEPSC_copy1[5]=W_Coef[4]

FitsEPSC_copy1[6]=(W_coef[2]*(-1*W_coef[1])+W_coef[4]*(-1*W_coef[3]))/((-1*W_coef[1])+(-1*W_coef[3]))


end


function Rep3_200()

Wave Copy_0_200,Copy_1_200,Copy_2_200,W_Coef, FitsEPSC_copy2,fit_EPSC1st_copy2, fitmono_EPSC1st_copy2, FitsEPSC_copy0, FitsEPSC_copy1

Variable Beginning, Ending

Beginning=100370

Ending=100673

Duplicate/R= [Beginning,Ending] Copy_2_200,EPSC1st_copy2

Display EPSC1st_copy2

make/o/n=7 FitsEPSC_copy2

Wavestats EPSC1st_copy2

CurveFit exp_XOffset EPSC1st_copy2 [V_minRowLoc,V_minRowLoc+160] /D

FitsEPSC_copy2[0]=W_Coef[1]

FitsEPSC_copy2[1]=W_Coef[2]

wave fit_EPSC1st_copy2, fitmono_EPSC1st_copy2

Duplicate/O fit_EPSC1st_copy2, 'fitmono_EPSC1st_copy2'


CurveFit dblexp_XOffset EPSC1st_copy2 [V_minRowLoc,V_minRowLoc+200] /D

FitsEPSC_copy2[2]=W_Coef[1]

FitsEPSC_copy2[3]=W_Coef[2]
```

```
FitsEPSC_copy2[4]=W_Coef[3]

FitsEPSC_copy2[5]=W_Coef[4]

FitsEPSC_copy2[6]=(W_coef[2]*(-1*W_coef[1])+W_coef[4]*(-1*W_coef[3]))/((-1*W_coef[1])+(-1*W_coef[3]))


edit FitsEPSC_copy0, FitsEPSC_copy1, FitsEPSC_copy2

end


/////////-------------------------------------------------------------------------------///////////

Function GettheEPCSs_300Hz()

Rep1_300()

//Rep2_100()

//Rep3_100()

Wave FitsEPSC_copy0

edit FitsEPSC_copy0

//edit FitsEPSC_copy1

//edit FitsEPSC_copy2

end


function Rep1_300()

Wave avg_0_300_env,Copy_1_300,Copy_2_300,W_Coef, FitsEPSC_copy0,fit_EPSC1st_copy0, fitmono_EPSC1st_copy0, EPSC1st_copy0

Variable Beginning, Ending


Make/O/N=5 W_Coef


Beginning=2100210

Ending=2100572

Duplicate/R= [Beginning,Ending] avg_0_300_env,EPSC1st_copy0

Display EPSC1st_copy0

make/o/n=7 FitsEPSC_copy0

Wavestats EPSC1st_copy0
```

```
CurveFit exp_XOffset EPSC1st_copy0 [V_minRowLoc,V_minRowLoc+80] /D
FitsEPSC_copy0[0]=W_Coef[1]
FitsEPSC_copy0[1]=W_Coef[2]
wave fit_EPSC1st_copy0, fitmono_EPSC1st_copy0
Duplicate/O fit_EPSC1st_copy0, 'fitmono_EPSC1st_copy0'

CurveFit dblexp_XOffset EPSC1st_copy0 [V_minRowLoc,V_minRowLoc+80] /D
FitsEPSC_copy0[2]=W_Coef[1]
FitsEPSC_copy0[3]=W_Coef[2]
FitsEPSC_copy0[4]=W_Coef[3]
FitsEPSC_copy0[5]=W_Coef[4]
FitsEPSC_copy0[6]=(W_coef[2]*(-1*W_coef[1])+W_coef[4]*(-1*W_coef[3]))/((-1*W_coef[1])+(-
1*W_coef[3]))

end

function Rep2_300()
Wave Copy_0_300,Copy_1_300,Copy_2_300,W_Coef, FitsEPSC_copy0,fit_EPSC1st_copy1,
fitmono_EPSC1st_copy1, EPSC1st_copy0
Variable Beginning, Ending
Beginning=100308
Ending=1005247
Duplicate/R= [Beginning,Ending] Copy_1_300,EPSC1st_copy1
Display EPSC1st_copy1
make/o/n=7 FitsEPSC_copy1
Wavestats EPSC1st_copy1
CurveFit exp_XOffset EPSC1st_copy1 [V_minRowLoc,V_minRowLoc+80] /D
FitsEPSC_copy1[0]=W_Coef[1]
FitsEPSC_copy1[1]=W_Coef[2]
wave fit_EPSC1st_copy1, fitmono_EPSC1st_copy1
Duplicate/O fit_EPSC1st_copy1, 'fitmono_EPSC1st_copy1'
```

```
CurveFit dblexp_XOffset EPSC1st_copy1 [V_minRowLoc,V_minRowLoc+80] /D

FitsEPSC_copy1[2]=W_Coef[1]

FitsEPSC_copy1[3]=W_Coef[2]

FitsEPSC_copy1[4]=W_Coef[3]

FitsEPSC_copy1[5]=W_Coef[4]

FitsEPSC_copy1[6]=(W_coef[2]*(-1*W_coef[1])+W_coef[4]*(-1*W_coef[3]))/((-1*W_coef[1])+(-
1*W_coef[3]))


end


function Rep3_300()

Wave Copy_0_300,Copy_1_300,Copy_2_300,W_Coef, FitsEPSC_copy2,fit_EPSC1st_copy2,
fitmono_EPSC1st_copy2, FitsEPSC_copy0, FitsEPSC_copy1

Variable Beginning, Ending

Beginning=100308

Ending=100524

Duplicate/R= [Beginning,Ending] Copy_2_300,EPSC1st_copy2

Display EPSC1st_copy2

make/o/n=7 FitsEPSC_copy2

Wavestats EPSC1st_copy2

CurveFit exp_XOffset EPSC1st_copy2 [V_minRowLoc,V_minRowLoc+80] /D

FitsEPSC_copy2[0]=W_Coef[1]

FitsEPSC_copy2[1]=W_Coef[2]

wave fit_EPSC1st_copy2, fitmono_EPSC1st_copy2

Duplicate/O fit_EPSC1st_copy2, 'fitmono_EPSC1st_copy2'


CurveFit dblexp_XOffset EPSC1st_copy2 [V_minRowLoc,V_minRowLoc+80] /D

FitsEPSC_copy2[2]=W_Coef[1]

FitsEPSC_copy2[3]=W_Coef[2]

FitsEPSC_copy2[4]=W_Coef[3]

FitsEPSC_copy2[5]=W_Coef[4]

FitsEPSC_copy2[6]=(W_coef[2]*(-1*W_coef[1])+W_coef[4]*(-1*W_coef[3]))/((-1*W_coef[1])+(-
1*W_coef[3]))
```

```
edit FitsEPSC_copy0, FitsEPSC_copy1, FitsEPSC_copy2
end

/////////-------------------------------------------------------------------------------///////////
Function GettheEPCSs_400Hz()
Rep1_400()
//Rep2_100()
//Rep3_100()
Wave FitsEPSC_copy0
edit FitsEPSC_copy0
//edit FitsEPSC_copy1
//edit FitsEPSC_copy2

end

function Rep1_400()
Wave avg_0_400_env,Copy_1_400,Copy_2_400,W_Coef, FitsEPSC_copy0,fit_EPSC1st_copy0,
fitmono_EPSC1st_copy0, EPSC1st_copy0
Variable Beginning, Ending

Make/O/N=5 W_Coef

Beginning=2100242
Ending=2100483
Duplicate/R= [Beginning,Ending] avg_0_400_env,EPSC1st_copy0
Display EPSC1st_copy0
make/o/n=7 FitsEPSC_copy0
Wavestats EPSC1st_copy0
CurveFit exp_XOffset EPSC1st_copy0 [V_minRowLoc,V_minRowLoc+40] /D
FitsEPSC_copy0[0]=W_Coef[1]
FitsEPSC_copy0[1]=W_Coef[2]
```

```
wave fit_EPSC1st_copy0, fitmono_EPSC1st_copy0

Duplicate/O fit_EPSC1st_copy0, 'fitmono_EPSC1st_copy0'


CurveFit dblexp_XOffset EPSC1st_copy0 [V_minRowLoc,V_minRowLoc+40] /D

FitsEPSC_copy0[2]=W_Coef[1]

FitsEPSC_copy0[3]=W_Coef[2]

FitsEPSC_copy0[4]=W_Coef[3]

FitsEPSC_copy0[5]=W_Coef[4]

FitsEPSC_copy0[6]=(W_coef[2]*(-1*W_coef[1])+W_coef[4]*(-1*W_coef[3]))/((-1*W_coef[1])+(-
1*W_coef[3]))



end


function Rep2_400()

Wave Copy_0_400,Copy_1_400,Copy_2_400,W_Coef, FitsEPSC_copy0,fit_EPSC1st_copy1,
fitmono_EPSC1st_copy1

Variable Beginning, Ending

Beginning=100312

Ending=100445

Duplicate/R= [Beginning,Ending] Copy_1_400,EPSC1st_copy1

Display EPSC1st_copy1

make/o/n=7 FitsEPSC_copy1

Wavestats EPSC1st_copy1

CurveFit exp_XOffset EPSC1st_copy1 [V_minRowLoc,V_minRowLoc+40] /D

FitsEPSC_copy1[0]=W_Coef[1]

FitsEPSC_copy1[1]=W_Coef[2]

wave fit_EPSC1st_copy1, fitmono_EPSC1st_copy1

Duplicate/O fit_EPSC1st_copy1, 'fitmono_EPSC1st_copy1'


CurveFit dblexp_XOffset EPSC1st_copy1 [V_minRowLoc,V_minRowLoc+40] /D

FitsEPSC_copy1[2]=W_Coef[1]
```

```
FitsEPSC_copy1[3]=W_Coef[2]

FitsEPSC_copy1[4]=W_Coef[3]

FitsEPSC_copy1[5]=W_Coef[4]

FitsEPSC_copy1[6]=(W_coef[2]*(-1*W_coef[1])+W_coef[4]*(-1*W_coef[3]))/((-1*W_coef[1])+(-
1*W_coef[3]))



end


function Rep3_400()

Wave Copy_0_400,Copy_1_400,Copy_2_400,W_Coef, FitsEPSC_copy2,fit_EPSC1st_copy2,
fitmono_EPSC1st_copy2, FitsEPSC_copy0, FitsEPSC_copy1

Variable Beginning, Ending

Beginning=100312

Ending=100445

Duplicate/R= [Beginning,Ending] Copy_2_400,EPSC1st_copy2

Display EPSC1st_copy2

make/o/n=7 FitsEPSC_copy2

Wavestats EPSC1st_copy2

CurveFit exp_XOffset EPSC1st_copy2 [V_minRowLoc,V_minRowLoc+40] /D

FitsEPSC_copy2[0]=W_Coef[1]

FitsEPSC_copy2[1]=W_Coef[2]

wave fit_EPSC1st_copy2, fitmono_EPSC1st_copy2

Duplicate/O fit_EPSC1st_copy2, 'fitmono_EPSC1st_copy2'


CurveFit dblexp_XOffset EPSC1st_copy2 [V_minRowLoc,V_minRowLoc+40] /D

FitsEPSC_copy2[2]=W_Coef[1]

FitsEPSC_copy2[3]=W_Coef[2]

FitsEPSC_copy2[4]=W_Coef[3]

FitsEPSC_copy2[5]=W_Coef[4]

FitsEPSC_copy2[6]=(W_coef[2]*(-1*W_coef[1])+W_coef[4]*(-1*W_coef[3]))/((-1*W_coef[1])+(-
1*W_coef[3]))
```

```
edit FitsEPSC_copy0, FitsEPSC_copy1, FitsEPSC_copy2
end
```
