# Supplementary material for: Non-calyceal inputs gate the timing of calyx of Held evoked MNTB output
Source: Commun Biol. 2026 May 22;9:697. doi: 10.1038/s42003-026-10321-w (PMC13197447; doi:10.1038/s42003-026-10321-w)
Supplement: Supplementary file 7 — Supplementary code [file 42003_2026_10321_MOESM7_ESM.pdf]

```
#pragma TextEncoding = "UTF-8"
#pragma rtGlobals=3				// Use modern global access method and strict
wave access
#pragma DefaultTab={3,20,4}		// Set default tab width in Igor Pro 9 and later

Function tr_10(Graphs,m)

String Graphs
String m

String Original_Waves
Original_Waves=WaveList("*",";",("WIN:"))

Variable n,i
Variable art_start=5295, art_end=5334

Make/O/N=30 I_min, I_min_norm
Make/O/N=(itemsinlist(Original_Waves)) zeroed

NewDataFolder $m

for(n=0;n<itemsinlist(Original_Waves);n+=1)
	Duplicate/O $StringFromList(n,Original_Waves), $"Copy"+num2str(n)
	Wave New_Wave=$"Copy"+num2str(n)

	WaveStats/Q/R=[3816,5295] New_Wave
	zeroed[n]=v_avg
	New_Wave-=zeroed[n]

	for(i=0;i<30;i+=1)
		New_Wave[art_start+i*5000,art_end+i*5000]=NaN
```

```
            WaveStats/Q/R=[art_end+i*5000,i*5000+10256] New_Wave
            I_min[i] = v_min
            I_min_norm[i] = I_min[i]/I_min[0]

        endfor

        Duplicate/O I_min, $"I_min"+num2str(n)
        Duplicate/O I_min_norm, $"I_min_norm"+num2str(n)
        Duplicate/O zeroed, $"zeroed"+num2str(n)
endfor



//Display
//for(n=0;n<itemsinlist(Original_Waves);n+=1)
        //AppendtoGraph $"I_min"+num2str(n)
//endfor
//ModifyGraph mode=4,marker=19
//ModifyGraph fSize=11,axThick=1.2,btLen=3

//Display
//for(n=0;n<itemsinlist(Original_Waves);n+=1)
        //AppendtoGraph $"I_min_norm"+num2str(n)
//endfor
//ModifyGraph mode=4,marker=19
//SetAxis left 0,*;DelayUpdate
//ModifyGraph fSize=11,axThick=1.2,btLen=3

for(n=0;n<itemsinlist(Original_Waves);n+=1)
        MoveWave $"Copy"+num2str(n), :$(m):
        MoveWave $"I_min"+num2str(n), :$(m):
        MoveWave $"I_min_norm"+num2str(n), :$(m):
        MoveWave $"zeroed"+num2str(n), :$(m):
```

```
endfor


end
```

---

```
Function tr_50(Graphs,m)

String Graphs
String m

String Original_Waves
Original_Waves=WaveList("*",";",("WIN:"))

Variable n,i
Variable art_start=5295, art_end=5342

Make/O/N=30 I_min, I_min_norm
Make/O/N=(itemsinlist(Original_Waves)) zeroed

NewDataFolder $m

for(n=0;n<itemsinlist(Original_Waves);n+=1)
	Duplicate/O $StringFromList(n,Original_Waves), $"Copy"+num2str(n)
	Wave New_Wave=$"Copy"+num2str(n)

	WaveStats/Q/R=[3816,5295] New_Wave
	zeroed[n]=v_avg
	New_Wave-=zeroed[n]
```

```
	for(i=0;i<30;i+=1)
		New_Wave[art_start+i*1000,art_end+i*1000]=NaN

		WaveStats/Q/R=[art_end+i*1000,i*1000+6236] New_Wave
		I_min[i] = v_min
		I_min_norm[i] = I_min[i]/I_min[0]

	endfor

	Duplicate/O I_min, $"I_min"+num2str(n)
	Duplicate/O I_min_norm, $"I_min_norm"+num2str(n)
	Duplicate/O zeroed, $"zeroed"+num2str(n)

	DeletePoints 38046,1000000, New_Wave
	DeletePoints 0,5041, New_Wave

endfor



//Display
//for(n=0;n<itemsinlist(Original_Waves);n+=1)
//				AppendtoGraph $"I_min"+num2str(n)
//endfor
//ModifyGraph mode=4,marker=19
//ModifyGraph fSize=11,axThick=1.2,btLen=3

//Display
//for(n=0;n<itemsinlist(Original_Waves);n+=1)
	//AppendtoGraph $"I_min_norm"+num2str(n)
//endfor
//ModifyGraph mode=4,marker=19
//SetAxis left 0,*;DelayUpdate
```

```
//ModifyGraph fSize=11,axThick=1.2,btLen=3

for(n=0;n<itemsinlist(Original_Waves);n+=1)
	MoveWave $"Copy"+num2str(n), :$(m):
	MoveWave $"I_min"+num2str(n), :$(m):
	MoveWave $"I_min_norm"+num2str(n), :$(m):
	MoveWave $"zeroed"+num2str(n), :$(m):

endfor


end
```

---

```
Function tr_100(Graphs,m)

String Graphs
String m

String Original_Waves
Original_Waves=WaveList("*",";",("WIN:"))

Variable n,i
Variable art_start=5295, art_end=5341

Make/O/N=30 I_min, I_min_norm
Make/O/N=(itemsinlist(Original_Waves)) zeroed

NewDataFolder $m
```

```
for(n=0;n<itemsinlist(Original_Waves);n+=1)
	Duplicate/O $StringFromList(n,Original_Waves), $"Copy"+num2str(n)
	Wave New_Wave=$"Copy"+num2str(n)

	WaveStats/Q/R=[3816,5295] New_Wave
	zeroed[n]=v_avg
	New_Wave-=zeroed[n]

	for(i=0;i<30;i+=1)
		New_Wave[art_start+i*500,art_end+i*500]=NaN

		WaveStats/Q/R=[art_end+i*500,i*500+5743] New_Wave
		I_min[i] = v_min
		I_min_norm[i] = I_min[i]/I_min[0]

	endfor

	Duplicate/O I_min, $"I_min"+num2str(n)
	Duplicate/O I_min_norm, $"I_min_norm"+num2str(n)
	Duplicate/O zeroed, $"zeroed"+num2str(n)

	DeletePoints 25095,1000000, New_Wave
	DeletePoints 0,5041, New_Wave
endfor


//Display
//for(n=0;n<itemsinlist(Original_Waves);n+=1)
	AppendtoGraph $"I_min"+num2str(n)
//endfor
//ModifyGraph mode=4,marker=19
//ModifyGraph fSize=11,axThick=1.2,btLen=3
```

```
//Display
//for(n=0;n<itemsinlist(Original_Waves);n+=1)
	AppendtoGraph $"I_min_norm"+num2str(n)
//endfor
//ModifyGraph mode=4,marker=19
//SetAxis left 0,*;DelayUpdate
//ModifyGraph fSize=11,axThick=1.2,btLen=3

for(n=0;n<itemsinlist(Original_Waves);n+=1)
	MoveWave $"Copy"+num2str(n), :$(m):
	MoveWave $"I_min"+num2str(n), :$(m):
	MoveWave $"I_min_norm"+num2str(n), :$(m):
	MoveWave $"zeroed"+num2str(n), :$(m):

endfor


end
```

---

```
Function tr_200(Graphs,m)

String Graphs
String m

String Original_Waves
Original_Waves=WaveList("*",";",("WIN:"))
```

```
Variable n,i
Variable art_start=5295, art_end=5343

Make/O/N=30 I_min, I_min_norm
Make/O/N=(itemsinlist(Original_Waves)) zeroed

NewDataFolder $m

for(n=0;n<itemsinlist(Original_Waves);n+=1)
	Duplicate/O $StringFromList(n,Original_Waves), $"Copy"+num2str(n)
	Wave New_Wave=$"Copy"+num2str(n)

	WaveStats/Q/R=[3816,5295] New_Wave
	zeroed[n]=v_avg
	New_Wave-=zeroed[n]

	for(i=0;i<30;i+=1)
		New_Wave[art_start+i*250,art_end+i*250]=NaN

		WaveStats/Q/R=[art_end+i*250,i*250+5544] New_Wave
		I_min[i] = v_min
		I_min_norm[i] = I_min[i]/I_min[0]

	endfor

	Duplicate/O I_min, $"I_min"+num2str(n)
	Duplicate/O I_min_norm, $"I_min_norm"+num2str(n)
	Duplicate/O zeroed, $"zeroed"+num2str(n)

	DeletePoints 16761,1000000, New_Wave
	DeletePoints 0,5041, New_Wave
endfor
```

```
//Display
//for(n=0;n<itemsinlist(Original_Waves);n+=1)
	//AppendtoGraph $"I_min"+num2str(n)
//endfor
//ModifyGraph mode=4,marker=19
//ModifyGraph fSize=11,axThick=1.2,btLen=3

//Display
//for(n=0;n<itemsinlist(Original_Waves);n+=1)
	//AppendtoGraph $"I_min_norm"+num2str(n)
//endfor
//ModifyGraph mode=4,marker=19
//SetAxis left 0,*;DelayUpdate
//ModifyGraph fSize=11,axThick=1.2,btLen=3

for(n=0;n<itemsinlist(Original_Waves);n+=1)
	MoveWave $"Copy"+num2str(n), :$(m):
	MoveWave $"I_min"+num2str(n), :$(m):
	MoveWave $"I_min_norm"+num2str(n), :$(m):
	MoveWave $"zeroed"+num2str(n), :$(m):

endfor



end
```

```
Function tr_300(Graphs,m)

String Graphs
String m

String Original_Waves
Original_Waves=WaveList("*",";",("WIN:"))

Variable n,i
	Variable art_start=5295, art_end=5338



Make/O/N=30 I_min, I_min_norm
Make/O/N=(itemsinlist(Original_Waves)) zeroed

NewDataFolder $m

for(n=0;n<itemsinlist(Original_Waves);n+=1)
	Duplicate/O $StringFromList(n,Original_Waves), $"Copy"+num2str(n)
	Wave New_Wave=$"Copy"+num2str(n)

	WaveStats/Q/R=[3816,5295] New_Wave
	zeroed[n]=v_avg
	New_Wave-=zeroed[n]

	for(i=0;i<30;i+=1)
		New_Wave[art_start+i*165,art_end+i*165]=NaN

		WaveStats/Q/R=[art_end+i*165,i*165+5457] New_Wave
		I_min[i] = v_min
		I_min_norm[i] = I_min[i]/I_min[0]
```

```
	endfor

	Duplicate/O I_min, $"I_min"+num2str(n)
	Duplicate/O I_min_norm, $"I_min_norm"+num2str(n)
	Duplicate/O zeroed, $"zeroed"+num2str(n)

	DeletePoints 13166,1000000, New_Wave
	DeletePoints 0,5112, New_Wave
endfor


//Display
//for(n=0;n<itemsinlist(Original_Waves);n+=1)
//	AppendtoGraph $"I_min"+num2str(n)
//endfor
//ModifyGraph mode=4,marker=19
//ModifyGraph fSize=11,axThick=1.2,btLen=3

//Display
//for(n=0;n<itemsinlist(Original_Waves);n+=1)
	AppendtoGraph $"I_min_norm"+num2str(n)
//endfor
//ModifyGraph mode=4,marker=19
//SetAxis left 0,*;DelayUpdate
//ModifyGraph fSize=11,axThick=1.2,btLen=3

for(n=0;n<itemsinlist(Original_Waves);n+=1)
	MoveWave $"Copy"+num2str(n), :$(m):
	MoveWave $"I_min"+num2str(n), :$(m):
	MoveWave $"I_min_norm"+num2str(n), :$(m):
	MoveWave $"zeroed"+num2str(n), :$(m):
```

```
endfor

end
```

_____

```
Function tr_400(Graphs,m)

String Graphs
String m

String Original_Waves
Original_Waves=WaveList("*",";",("WIN:"))

Variable n,i
Variable art_start=5296, art_end=5341

Make/O/N=30 I_min, I_min_norm
Make/O/N=(itemsinlist(Original_Waves)) zeroed

NewDataFolder $m

for(n=0;n<itemsinlist(Original_Waves);n+=1)
	Duplicate/O $StringFromList(n,Original_Waves), $"Copy"+num2str(n)
	Wave New_Wave=$"Copy"+num2str(n)

	WaveStats/Q/R=[3816,5295] New_Wave
	zeroed[n]=v_avg
```

```
	New_Wave-=zeroed[n]

	for(i=0;i<30;i+=1)
		New_Wave[art_start+i*125,art_end+i*125]=NaN

		WaveStats/Q/R=[art_end+i*125,i*125+5417] New_Wave
		I_min[i] = v_min
		I_min_norm[i] = I_min[i]/I_min[0]

	endfor

	Duplicate/O I_min, $"I_min"+num2str(n)
	Duplicate/O I_min_norm, $"I_min_norm"+num2str(n)
	Duplicate/O zeroed, $"zeroed"+num2str(n)

	DeletePoints 14000,1000000, New_Wave
	DeletePoints 0,4876, New_Wave
endfor


//Display
//for(n=0;n<itemsinlist(Original_Waves);n+=1)
//	AppendtoGraph $"I_min"+num2str(n)
//endfor
//ModifyGraph mode=4,marker=19
//ModifyGraph fSize=11,axThick=1.2,btLen=3

//Display
//for(n=0;n<itemsinlist(Original_Waves);n+=1)
//	AppendtoGraph $"I_min_norm"+num2str(n)
//endfor
//ModifyGraph mode=4,marker=19
```

```
//SetAxis left 0,*;DelayUpdate
//ModifyGraph fSize=11,axThick=1.2,btLen=3

for(n=0;n<itemsinlist(Original_Waves);n+=1)
	MoveWave $"Copy"+num2str(n), :$(m):
	MoveWave $"I_min"+num2str(n), :$(m):
	MoveWave $"I_min_norm"+num2str(n), :$(m):
	MoveWave $"zeroed"+num2str(n), :$(m):

endfor


end
```

---

```
Function VC_step()

Variable k, ind

String Original_Waves
Original_Waves=WaveList("*",";",("WIN:"))
String wave_top=StringFromList(k,Original_Waves)
Wave wave_top_name=$wave_top

Variable start_VC
WaveStats/Q/R=[3800,4000] wave_top_name
start_VC=V_avg
wave_top_name-=start_VC // set wave to 0 just before start of stimulus
```

```
ModifyGraph rgb=(0,0,0)

Make/O/N=3 Ceff
Make/O/N=3 passive
Make/O/T/N=3 descrip
Make/O/T/N=3 pass_desc
descrip={"C_weighted","C_fast","C_slow"}
pass_desc={"Ceff","Rin","tau"}

Make/O/N=3 tau
Make/O/T/N=3 desc_tau
desc_tau={"tau_weighted","t_fast","t_slow"}


Make/O/N=5 W_Coef

//FIND MIN
Variable min_pnt
WaveStats/Q/R=[4000,4100] wave_top_name
min_pnt=V_minloc/5e-6 // now in pnts
Variable start_decay=min_pnt+3 //START OF DECAY, plus 3 points after
Variable W_fitConstants

CurveFit dblexp_XOffset wave_top_name[start_decay,7000] /D
tau[0]=(W_coef[2]*(-1*W_coef[1])+W_coef[4]*(-1*W_coef[3]))/((-1*W_coef[1])+(-1*W_coef[3]))
passive[2]=(W_coef[2]*(-1*W_coef[1])+W_coef[4]*(-1*W_coef[3]))/((-1*W_coef[1])+(-
1*W_coef[3]))
tau[1]=W_coef[2]
tau[2]=W_coef[4]

Make/O/N=3 W_coef_fast, W_coef_slow
W_coef_fast[0]=W_coef[0]
```

```
W_coef_fast[1]=W_coef[1]
W_coef_fast[2]=W_coef[2]

W_coef_slow[0]=W_coef[0]
W_coef_slow[1]=W_coef[3]
W_coef_slow[2]=W_coef[4]

//fit_W_VC01_AV= W_coef[0]+W_coef[1]*exp(-(x-W_fitConstants[0])/W_coef[2])

//CREATE NEW GRAPH WITH FAST
Duplicate/O wave_top_name, new_exp_trace_fast
new_exp_trace_fast[start_decay,7000]=W_coef_fast[0]+W_coef_fast[1]*exp(-(x-(min_pnt*5e-6+3*5e-6))/W_coef_fast[2])
AppendtoGraph new_exp_trace_fast
ModifyGraph rgb(new_exp_trace_fast)=(1,16019,65535)
new_exp_trace_fast[start_decay,11999]+=W_coef[3]
new_exp_trace_fast[4004,11999]-=W_coef[3]
for(ind=0;ind<8000;ind+=1)
	if(new_exp_trace_fast[ind]>0)
		new_exp_trace_fast[ind]=0
	endif
endfor

//CREATE NEW GRAPH WITH SLOW
Duplicate/O wave_top_name, new_exp_trace_slow
new_exp_trace_slow[start_decay,7000]=W_coef_slow[0]+W_coef_slow[1]*exp(-(x-(min_pnt*5e-6+3*5e-6))/W_coef_slow[2])
AppendtoGraph new_exp_trace_slow
ModifyGraph rgb(new_exp_trace_slow)=(3,52428,1)
new_exp_trace_slow[start_decay,11999]+=W_coef[1]
new_exp_trace_slow[4004,11999]-=W_coef[1]
for(ind=0;ind<8000;ind+=1)
```

```
	if(new_exp_trace_slow[ind]>0)
		new_exp_trace_slow[ind]=0
	endif
endfor



//Edit desc_tau,tau
//Edit W_coef_mono, W_coef
Variable tau_weighted_3=tau[0]*3
Variable finish_weighted=0.02+tau_weighted_3

Variable tau_fast_3=tau[1]*3
Variable finish_fast=0.02+tau_fast_3

Variable tau_slow_3=tau[2]*3
Variable finish_slow=0.02+tau_slow_3

Make/O/N=3 tau_times_3
tau_times_3[0]=finish_weighted
tau_times_3[1]=finish_fast
tau_times_3[2]=finish_slow



Integrate wave_top_name/D=wave_top_name_INT;DelayUpdate
//Display wave_top_name_INT
Ceff[0]=(-wave_top_name_INT(finish_weighted)*1e12+wave_top_name_INT[4000]*1e12)/0.005
passive[0]=(-
wave_top_name_INT(finish_weighted)*1e12+wave_top_name_INT[4000]*1e12)/0.005
Integrate new_exp_trace_fast/D=new_exp_trace_fast_INT;DelayUpdate
//Display wave_top_name_INT
```

```
Ceff[1]=(-
new_exp_trace_fast_INT(finish_fast)*1e12+new_exp_trace_fast_INT[4000]*1e12)/0.005


Integrate new_exp_trace_slow/D=new_exp_trace_slow_INT;DelayUpdate

//Display wave_top_name_INT

Ceff[2]=(-
new_exp_trace_slow_INT(finish_slow)*1e12+new_exp_trace_slow_INT[4000]*1e12)/0.005


//Display wave_top_name_INT

//AppendtoGraph new_exp_trace_fast_INT

//AppendtoGraph new_exp_trace_slow_INT

//AppendToGraph/R wave_top_name_INT

//AppendToGraph/R new_exp_trace_fast_INT

//AppendToGraph/R new_exp_trace_slow_INT



//Calculate Rin

Variable before, after, diff

WaveStats/Q/R=[3800,4000] wave_top_name

before=V_avg


WaveStats/Q/R=[7500,8000] wave_top_name

after=V_avg


diff=before-after

passive[1]=5/diff/1000000000


Edit pass_desc,passive


end
```

```
/////// Macro and funciton to extract charge from stimulation trains

Function charge(Graphs,m,freq) //charge("W","freq","freq")

String Graphs
String m
String freq

String Graph_folder=m+"_Graphs"
NewDataFolder $m
NewDataFolder $Graph_folder

String Trains
Trains=WaveList("*",";",("WIN:"))

Variable art_start=5290, art_end=5348 //Set start and end of artifact

Variable pnt_diff
if(str2num(freq)==10)
	pnt_diff=5000
elseif(str2num(freq)==50)
	pnt_diff=1000
elseif(str2num(freq)==100)
	pnt_diff=500
elseif(str2num(freq)==200)
```

```
		pnt_diff=250
	elseif(str2num(freq)==300)
		pnt_diff=165
	elseif(str2num(freq)==400)
		pnt_diff=125
	endif

Variable n,k

for(k=0;k<itemsinlist(Trains);k+=1)

Make/O/N=(itemsinlist(Trains)) sum_INT_orig,sum_INT_envelope,sum_INT_null
Make/O/N=30 last_pnt_30, max_EPSC, max_EPSC_ratio

	Duplicate/O $stringfromlist(k,Trains), platz
	WaveStats/Q/R=[3500,4000] platz
	platz-=V_avg

	Duplicate/O platz, platz_original
	Duplicate/O platz, platz_envelope
	Duplicate/O platz, platz_NaN
	Duplicate/O platz, platz_null

	for(n=0;n<30;n+=1)
		last_pnt_30[n]=platz[art_start+n*pnt_diff-1]
		platz_envelope[art_start+n*pnt_diff,art_end+n*pnt_diff]=last_pnt_30[n]
		// Here we find EPSC maxima
		WaveStats/Q/R=[art_end+n*pnt_diff,art_start+(n+1)*pnt_diff] platz_envelope
		max_EPSC[n]=V_min
		max_EPSC_ratio[n]=max_EPSC[n]/max_EPSC[0]

		platz_NaN[art_start+n*pnt_diff,art_end+n*pnt_diff]=NaN
```

```
			platz_null[art_start+n*pnt_diff,art_end+n*pnt_diff]=0
		endfor
//endfor

//Integrations

Make/O/N=30 int_original, int_envelope, int_null

Integrate platz_original/D=orig_INT
//Wave orig_INT=orig_INT_platz
Integrate platz_envelope/D=env_INT
//Wave env_INT=env_INT_platz
Integrate platz_null/D=null_INT
//Wave null_INT=null_INT_platz

for(n=0;n<30;n+=1)
		int_original[n]=orig_INT[art_start+(n+1)*pnt_diff]-orig_INT[art_start+n*pnt_diff]
		int_envelope[n]=env_INT[art_start+(n+1)*pnt_diff]-env_INT[art_start+n*pnt_diff]
		int_null[n]=null_INT[art_start+(n+1)*pnt_diff]-null_INT[art_start+n*pnt_diff]
endfor

DeletePoints 0,3500, platz_envelope, platz_NaN, platz_null, platz_original
DeletePoints 0,3500, orig_INT, env_INT, null_INT

//Copy and move EPSC maxima
Rename max_EPSC, $"max_EPSC_"+freq+"_rep_"+num2str(k)
MoveWave $"max_EPSC_"+freq+"_rep_"+num2str(k), :$(m):
Rename max_EPSC_ratio, $"max_EPSC_ratio_"+freq+"_rep_"+num2str(k)
MoveWave $"max_EPSC_ratio_"+freq+"_rep_"+num2str(k), :$(m):

//Copy and move Graphs
Duplicate/O platz_original, $"platz_original_"+freq+"_rep_"+num2str(k)
```

```
MoveWave $"platz_original_"+freq+"_rep_"+num2str(k), :$(Graph_folder):
Duplicate/O platz_envelope, $"platz_envelope_"+freq+"_rep_"+num2str(k)
MoveWave $"platz_envelope_"+freq+"_rep_"+num2str(k), :$(Graph_folder):
Duplicate/O platz_NaN, $"platz_NaN_"+freq+"_rep_"+num2str(k)
MoveWave $"platz_NaN_"+freq+"_rep_"+num2str(k), :$(Graph_folder):
Duplicate/O platz_null, $"platz_null_"+freq+"_rep_"+num2str(k)
MoveWave $"platz_null_"+freq+"_rep_"+num2str(k), :$(Graph_folder):

//Copy and move Integration Waves
Duplicate/O orig_INT, $"orig_INT_"+freq+"_rep_"+num2str(k)
MoveWave $"orig_INT_"+freq+"_rep_"+num2str(k), :$(Graph_folder):
Duplicate/O env_INT, $"env_INT_"+freq+"_rep_"+num2str(k)
MoveWave $"env_INT_"+freq+"_rep_"+num2str(k), :$(Graph_folder):
Duplicate/O null_INT, $"null_INT_"+freq+"_rep_"+num2str(k)
MoveWave $"null_INT_"+freq+"_rep_"+num2str(k), :$(Graph_folder):

//Copy and move Integrations
Rename int_original, $"int_original_"+freq+"_rep_"+num2str(k)
MoveWave $"int_original_"+freq+"_rep_"+num2str(k), :$(m):
Rename int_envelope, $"int_envelope_"+freq+"_rep_"+num2str(k)
MoveWave $"int_envelope_"+freq+"_rep_"+num2str(k), :$(m):
Rename int_null, $"int_null_"+freq+"_rep_"+num2str(k)
MoveWave $"int_null_"+freq+"_rep_"+num2str(k), :$(m):

endfor

//Copy and move Integration Sums
Rename sum_INT_orig, $"sum_INT_orig_"+freq+"_rep_"+num2str(k)
MoveWave $"sum_INT_orig_"+freq+"_rep_"+num2str(k), :$(m):
Rename sum_INT_envelope, $"sum_INT_envelope_"+freq+"_rep_"+num2str(k)
MoveWave $"sum_INT_envelope_"+freq+"_rep_"+num2str(k), :$(m):
Rename sum_INT_null, $"sum_INT_null_"+freq+"_rep_"+num2str(k)
```

```
MoveWave $"sum_INT_null_"+freq+"_rep_"+num2str(k), :$(m):



Movewave env_INT, :$(m):
Movewave last_pnt_30, :$(m):
Movewave null_INT, :$(m):
Movewave orig_INT, :$(m):
Movewave platz, :$(m):
Movewave platz_envelope, :$(m):
Movewave platz_NaN, :$(m):
Movewave platz_null, :$(m):
Movewave platz_original, :$(m):
end
```
